# Supplementary material for: Overlapping speckle correlation algorithm for high-resolution imaging and tracking of objects in unknown scattering media
Source: Nat Commun. 2023 Nov 25;14:7742. doi: 10.1038/s41467-023-43674-5 (PMC10676403; doi:10.1038/s41467-023-43674-5)
Supplement: Supplementary file 1 — Supplementary Information [file 41467_2023_43674_MOESM1_ESM.pdf]

Supplementary Information

Overlapping speckle correlation algorithm for high-resolution imaging and tracking of objects in unknown scattering media

Authors

Yaoyao Shi[1,2,3]*, Wei Sheng[1], Yangyang Fu[1]* & Youwen Liu[1]*

Affiliations

[1]College of Physics, Nanjing University of Aeronautics and Astronautics, Nanjing 210016, China.

[2]College of Astronautics, Nanjing University of Aeronautics and Astronautics, Nanjing 210016, China.

[3]Key Laboratory of Radar Imaging and Microwave Photonics, Ministry of Education, Nanjing University of Aeronautics and Astronautics, Nanjing 210016, China.

*e-mail: syy411@nuaa.edu.cn; yyfu@nuaa.edu.cn; ywliu@nuaa.edu.cn

Contents

Supplementary Note 1: Experimental setup

The photograph of the experimental setup is shown.

Supplementary Note 2: Overlapping speckle correlation algorithm

The algorithm flow chart of the overlapping speckle correlation is shown.

Supplementary Note 3: Autocorrelation construction with a simplified trajectory

Two examples of autocorrelation construction with trajectories of U and T shapes are shown.

Supplementary Note 4: Recovered trajectories

The trajectories of the moving objects in the experiments are partially shown.

Supplementary Note 5: Assumption of uniform $I_S(x, y)$

The feasibility of the assumption that the intensity distribution $I_S(x, y)$ is nearly uniform within a small region containing the object is experimentally confirmed.

Supplementary Note 6: The imaging depth limit

The experimental results for analysis of imaging depth limit are shown.

Supplementary Note 7: Imaging resolution

The experimental results for analysis of imaging resolution are shown.

Supplementary Note 8: Imaging of stationary object in scattering media

Imaging of a stationary object in a movable scattering medium is experimentally demonstrated.

Supplementary Note 9: Imaging of moving objects around corners

NLOS imaging of a moving object around a corner is experimentally demonstrated.

Supplementary Note 10: Scattering properties of the scattering samples

The scattering coefficient and the anisotropy coefficient of the parafilm, the chicken breast and the polyethylene foams are measured.

Supplementary Note 11: Generation of envelope and speckle

The generating process of the envelope and the speckle from a speckle image is shown.

Supplementary Note 12: The dispersion of white light in direct imaging experiments

The reason of the dispersion of white light in experiments of direct imaging is described.

Supplementary Note 13: The phase-retrieval algorithm

The iterative Fienup-type phase-retrieval algorithm we used is described.

Supplementary Note 1: Experimental setup

The photograph of the experimental setup is shown in Supplementary Fig. 1. The imaging system consists of an incoherent light source, an achromatic lens and a monochrome camera. The light sources in the experiments were a single-colour light-emitting diode (LED), a research arc lamp source, and a continuous laser for excitation of fluorescent beads. The scattering samples had gaps in their middle sections that were approximately equal to the thickness of the object and were held by a plate holder. The object was embedded in the scattering samples and controlled via a two-axis motorized precision translation stage.

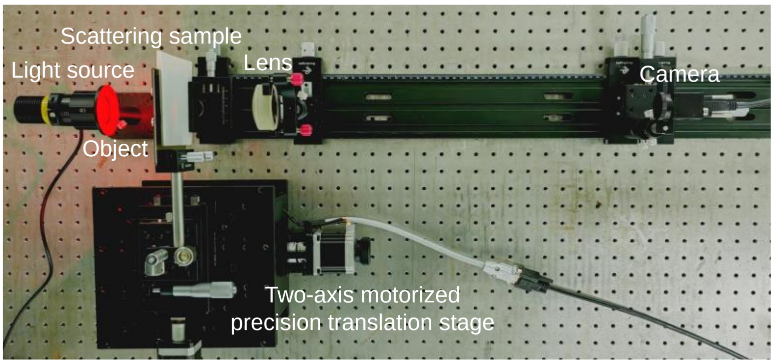

Supplementary Fig. 1 Experimental setup. The imaging system consists of an incoherent light source, a lens and a camera. The transmissive object on a negative resolution test target is hidden in the scattering sample and is moved via a two-axis motorized precision translation stage.

Supplementary Note 2: Overlapping speckle correlation algorithm

The algorithm flow chart of the overlapping speckle correlations, which is mentioned in the Methods of the manuscript, is shown in Supplementary Fig. 2.

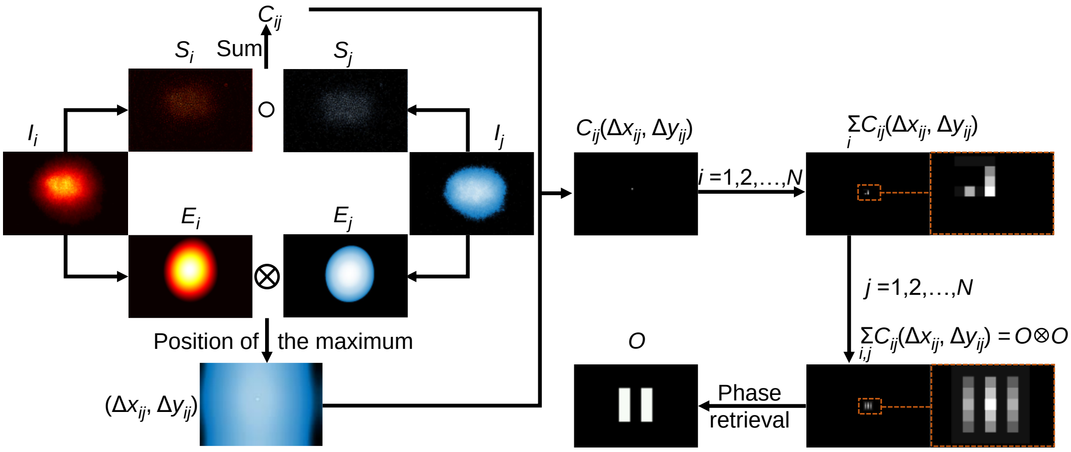

Supplementary Fig. 2 Algorithm flow chart of the overlapping speckle correlations. Two of the detected N speckle images I_i and I_j are numerically separated into envelopes E_i and E_j and speckles S_i and S_j. The Hadamard product $\circ$ is performed on S_i and S_j to obtain the overlapping speckle, and all pixel values of the overlapping speckle are summed to obtain a value C_{ij} of the object's autocorrelation. The cross-correlation $\otimes$ is performed on E_i and E_j to obtain the position (Δx_{ij}, Δy_{ij}) of value C_{ij} on the object's autocorrelation. Combining the calculated value and its position information, a pixel $C_{ij}(\Delta x_{ij}, \Delta y_{ij})$ of the object's autocorrelation is constructed. The object's autocorrelation $\Sigma C_{ij}(\Delta x_{ij}, \Delta y_{ij})$ is completely constructed by successively changing i and j from 1 to N. The object image O is recovered from the autocorrelation through a phase-retrieval algorithm.

Supplementary Note 3: Autocorrelation construction with a simplified trajectory

Two examples of autocorrelation construction with trajectories of U shape and T shape are shown in Supplementary Fig. 3. The construction method is described in the Methods of the manuscript.

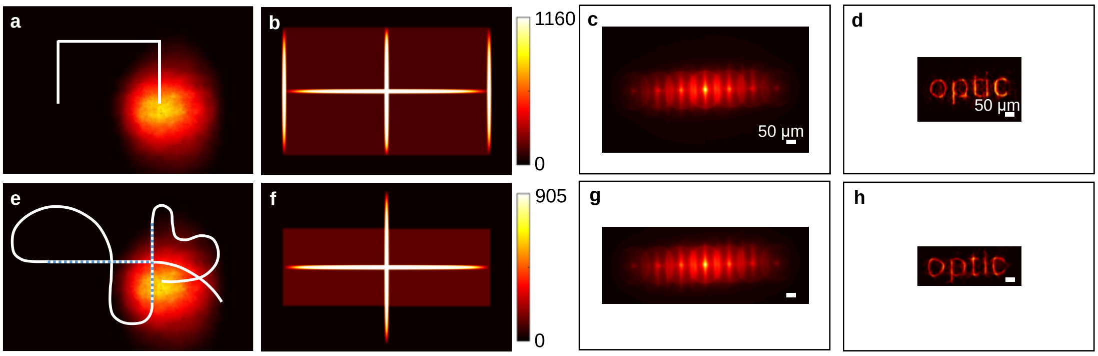

Supplementary Fig. 3 Autocorrelation construction with a simple trajectory. The object image can be reconstructed as long as the autocorrelation of the trajectory is large enough to cover the object's autocorrelation with sufficient resolution. **a** Speckle image from a hidden object. The trajectory extracted from the cross-correlations between every two envelopes is in a U shape. The overall size of the trajectory is slightly larger than the speckle pattern. **b** Autocorrelation of the U-shaped trajectory in (**a**). **c** The constructed autocorrelation of the object with a sampling area determined by (**b**). **d** The object image recovered from (**c**). **e** Speckle image from a spontaneously moving object. The trajectory is recovered from the cross-correlations between every two envelopes. **f** Autocorrelation of the partially selected trajectories shown as a blue dotted T shape in (**e**). **g** The object's autocorrelation with a sampling area determined by (**f**). **h** The object image recovered from (**g**). Scale bars: 50 μm.

Supplementary Note 4: Recovered trajectories

The trajectories of the moving objects in the experiments are recovered and partially shown in Supplementary Fig. 4. The related description is in the Methods of the manuscript. Since each position on the trajectory is only a camera pixel size, it is too small to see compared with the trajectory of a large overall size. Therefore, pixels around each position are coloured as a yellow square to highlight the positions. Besides, the real trajectories are shown as the red solid lines in Supplementary Fig. 4. The width of each red line is set approximately equal to the pixel width of the constructed autocorrelation, which is determined by the average interval between adjacent positions.

Supplementary Figures 4a-4c show the recovered trajectories of the 10 μm width object in 22 layers of parafilm, the 5 μm width object in 14 layers of parafilm and the 1 μm width object in 6 layers of parafilm. Supplementary Figures 4d and 4e are the recovered trajectories of a number-shaped 5 and five fluorescent beads. The results are in good agreement with the real trajectories in the experiments (red solid lines in Supplementary Fig. 4). In the experiments, as the thickness of the scattering medium increases and the object size decreases, the envelopes of the speckle images become slightly flatter. As a result, there is a slight drop in the accuracy of the recovered trajectories. Nevertheless, the autocorrelation construction is usually not affected since the inaccuracy is smaller than the pixel size of the autocorrelation.

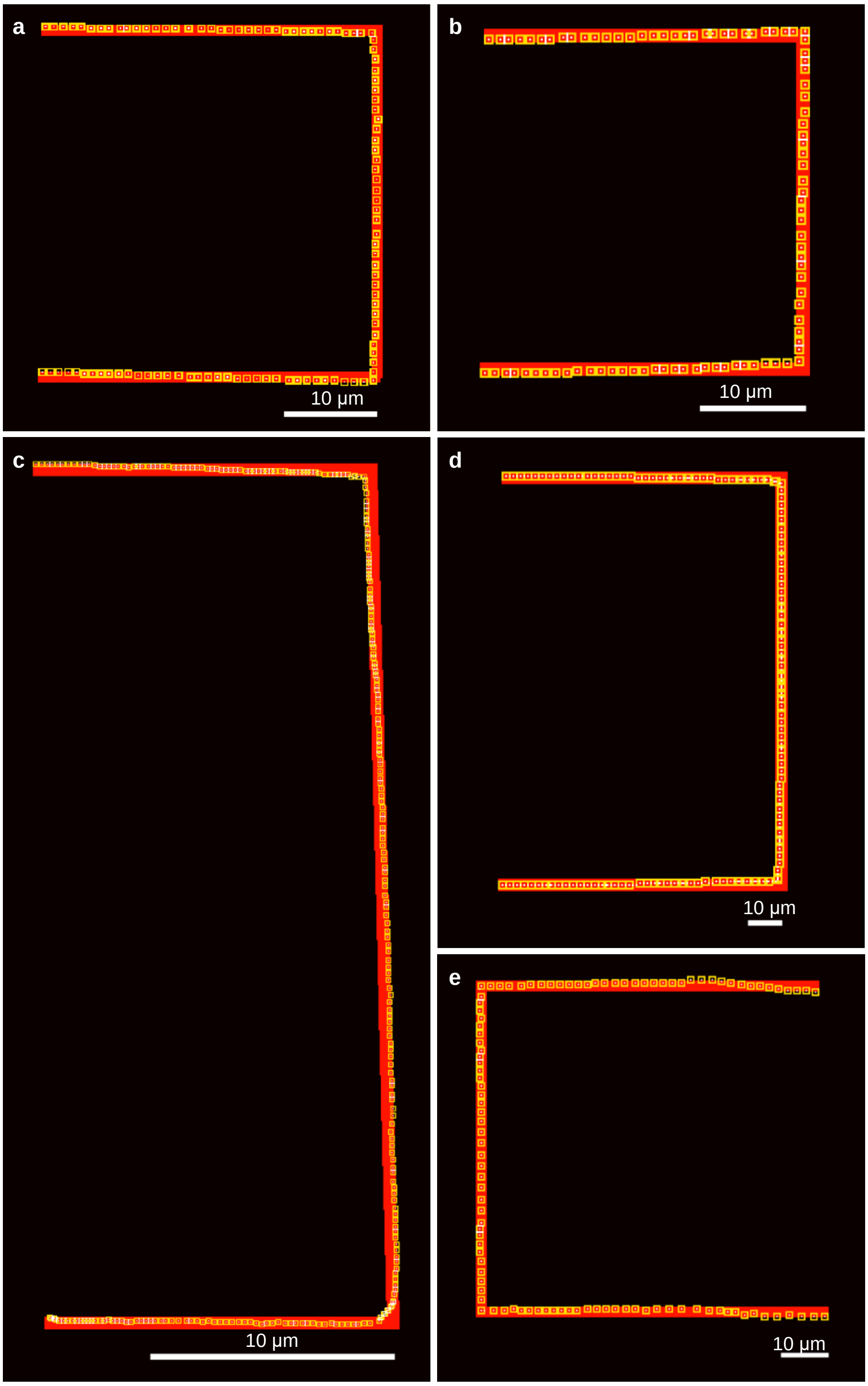

Supplementary Fig. 4 Recovered trajectories in the experiments. The relative positions are the white spots in the centre of the yellow squares. The red solid lines represent the real trajectories. The width of the red line is set approximately equal to the pixel width of the constructed autocorrelations. **a** The recovered trajectory of the object consisting of two 10 μm width lines and moved in 22 layers of parafilm. **b** The recovered trajectory of the object consisting of three 5 μm width lines and moved in 14 layers of parafilm. **c** The recovered trajectory of the object consisting of six 1 μm width lines and moved in 6 layers of parafilm. **d** The recovered trajectory of a number-shaped 5 and moved in 14 layers of parafilm. **e** The recovered trajectory of the fluorescent beads. Scale bars: 10 μm.

Supplementary Note 5: Assumption of uniform $I_S(x, y)$

To confirm the feasibility of the assumption above Eq. (5) that the intensity distribution $I_S(x, y)$ is nearly uniform within a small region containing the object, we detected the intensity distributions on the object plane by removing the latter half of the parafilm. As shown in Supplementary Figs. 5a-5c, a transmissive object consisting of three lines was illuminated by a narrowband halo with intensity distribution $I_S(x, y)$ emitted from parafilm of 3, 7 and 11 layers, respectively. Supplementary Figures 5d-5f show the number-shaped object illuminated by a broadband halo with intensity distribution $I_S(x, y)$ emitted from parafilm of 3, 7 and 11 layers. Although the intensity distributions shown in Supplementary Fig. 5 are not completely uniform, the intensity fluctuation is minimal relative to the overall intensity. Thus, the intensity distribution $I_S(x, y)$ within a small region containing the object can be assumed to be nearly uniform.

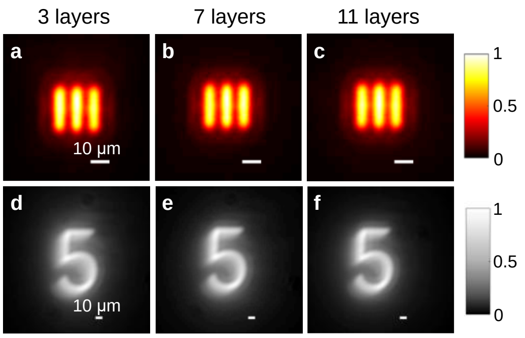

Supplementary Fig. 5 Intensity distribution on the object plane. a-c Intensity distributions of an object consisting of three 5 μm width lines that are illuminated by a narrowband halo with intensity distribution $I_S(x, y)$ emitted from parafilm of 3 (**a**), 7 (**b**) and 11 (**c**) layers. **d-f** Intensity distributions of the number-shaped object illuminated via a broadband halo with intensity distribution $I_S(x, y)$ emitted from parafilm of 3 (**d**), 7 (**e**) and 11 (**f**) layers. Scale bars: 10 μm.

Supplementary Note 6: The imaging depth limit

To explore the imaging depth limit of speckle kinetography, imaging of an object consisting of three 25 μm width lines moving inside parafilm of 2 to 30 layers is experimentally implemented. The structural similarity index measurement (SSIM) of the constructed autocorrelation decreases as the sample thickness L increases (Supplementary Fig. 6a). When the sample thickness reaches 28 layers, the object's autocorrelation becomes too blurry to image. Therefore, the maximum imaging depth here is about 26 layers of parafilm, corresponding to $19.9l_s$ and $6.2l_t$. To determine the main limiting factors, the envelope expansion, the relative displacement error and the speckle contrast versus the sample thickness are analyzed. The full width at half-maximum W of the envelope broadens as the sample thickness L increases (Supplementary Fig. 6b). But the entire envelope can still be measured when the parafilm is 28 layers, so it does not have obvious impact on imaging. The relative displacement error E fluctuates slightly as L increases (Supplementary Fig. 6c). But the fluctuation keeps within ±4 camera pixels, which is smaller than the pixel size of the object's autocorrelation, corresponding to 16 camera pixels in these experiments. Thus, it does not cause misplacement to autocorrelation pixels. The speckle contrast decreases as L increases and it quickly drops below 0.1 from 26 to 30 layers (Supplementary Fig. 6d). The low contrast causes the speckles to overlap with each other even when the corresponding objects do not overlap. In this case, the small values of the object's autocorrelation become large, which is consistent with the blurred autocorrelation constructed at 28 layers (Supplementary Fig. 6a). It causes the imaging to fail. In conclusion, although the above three factors all limit the imaging depth, the speckle contrast dominates.

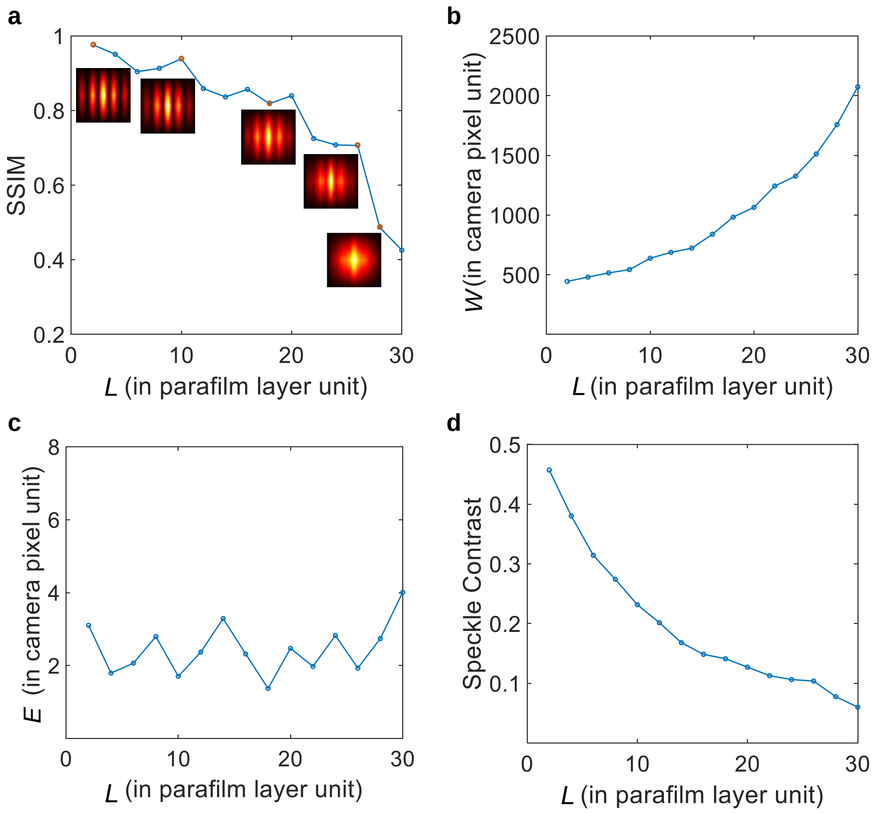

Supplementary Fig. 6 The influence of medium thickness on four factors. Imaging of an object consisting of three 25 μm width lines moved in parafilm of 2 to 30 layers is experimentally performed. **a** The SSIM of the constructed autocorrelation versus the sample thickness L. The insets are the constructed autocorrelations from 2 to 26 layers with an interval of 8 layers. Their corresponding SSIMs are respectively marked with red dots. The autocorrelation (the last inset) becomes too blurry to image when L reaches 28 layers. The corresponding SSIM is obviously smaller than other SSIMs and is marked with a red dot. **b** The FWHM W of the envelopes versus L. **c** The relative displacement accuracy E versus L. **d** The speckle contrast versus L. The speckle contrast is below 0.1 when L reaches 28 layers.

To eliminate the anisotropic effects of the scattering sample, imaging of the above-mentioned 25 μm widths object embedded in homogeneous static isotropic polyethylene foams (Supplementary Figs. 7a and 7b) is experimentally performed. When the polyethylene foam is 15 mm thick, the speckle images with entire envelope and sufficient speckle contrast are recorded under narrowband illumination (single-colour LED of 625 nm nominal wavelength and 17 nm bandwidth, 920 mW) and broadband illumination (research arc lamp source of 260 to 2500 nm wavelength, 150 W), as shown in Supplementary Figs. 7c and 7d. The constructed autocorrelations and recovered object images are shown in Supplementary Figs. 7e-7h. However, when the polyethylene foam is 20 mm thick, the contrast of the speckle images is too low to image. Therefore, the maximum imaging depth here is between 15 mm to 20 mm

thicknesses of polyethylene foams, corresponding to 5.1~6.8l_s and 5.0~6.7l_t. Notably, the maximum imaging depths in polyethylene foam (5.0~6.7l_t) and parafilm (6.2l_t) are consistent after eliminating the anisotropic effects. Therefore, we conclude that the imaging depth limit of speckle kinetography is about 6l_t. The scattering properties of the scattering samples are described in Supplementary Note 10.

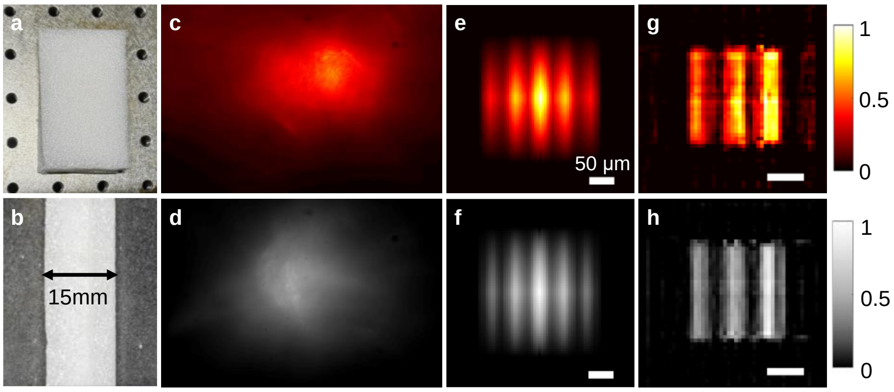

Supplementary Fig. 7 Imaging of an object embedded in 15 mm thick polyethylene foams. **a** and **b** Front (**a**) and side (**b**) photographs of the polyethylene foams with 15 mm thickness. **c** One of the recorded speckle images under narrowband illumination. **d** One of the recorded speckle images under broadband illumination. **e** The object's autocorrelation constructed from the recorded speckle images in (**c**). **f** The object's autocorrelation constructed from the recorded speckle images in (**d**). **g** The object image recovered from (**e**). **h** The object image recovered from (**f**). Scale bars: 50 μm.

Supplementary Note 7: Imaging resolution

In scattering imaging, the resolution limit R_{IS} of the imaging system determines the minimum detectable speckle grain size. Since the speckle grain size D increases with the object's line width LW (Supplementary Fig. 8a), the minimum detectable speckle grain size determines the resolution limit R_{obj} on the object plane. Therefore, R_{IS} determines R_{obj}. In addition, the speckle grain size D decreases as the medium thickness L increases (Supplementary Fig. 8b), that is, the same sized speckle scattered from the thicker sample should be generated from objects with the larger line width. Therefore, the resolution limit R_{obj} on the object plane becomes larger when the medium thickness L increases. Therefore, L also affects R_{obj}. In conclusion, R_{obj} is determined by R_{IS} and L.

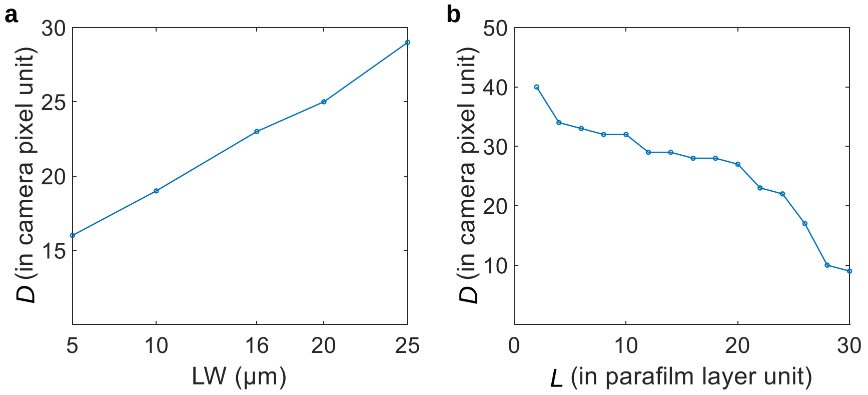

Supplementary Fig. 8 The speckle grain size versus the object size and the medium thickness. a The average speckle grain size D increases with the object's line width LW. The scattering sample is parafilm of 14 layers. The objects consist of three lines with 5, 10, 16, 20 and 25 μm widths, respectively. **b** The average speckle grain size D decreases as the medium thickness L increases. The scattering samples are parafilm of 2 to 30 layers, in steps of 2 layers. A transmissive object consisting of three 25 μm width lines is embedded in the middle of the parafilm.

Supplementary Note 8: Imaging of stationary object in scattering media

We experimentally performed imaging of a stationary object in a movable scattering medium. The experimental setup is shown in Supplementary Fig. 9a. A stationary object consisting of 2 transmissive lines (10 μm widths, 10 μm interval, 30 μm heights) on the negative resolution test targets was embedded in the middle of a parafilm with 14 layers. The parafilm was moved in a U-shaped trajectory in the plane perpendicular to the optical axis with a displacement of 1 μm between adjacent detections. According to the detected 105 speckle images, the object's autocorrelation and image were reconstructed through speckle kinetography (Supplementary Figs. 9b and 9c).

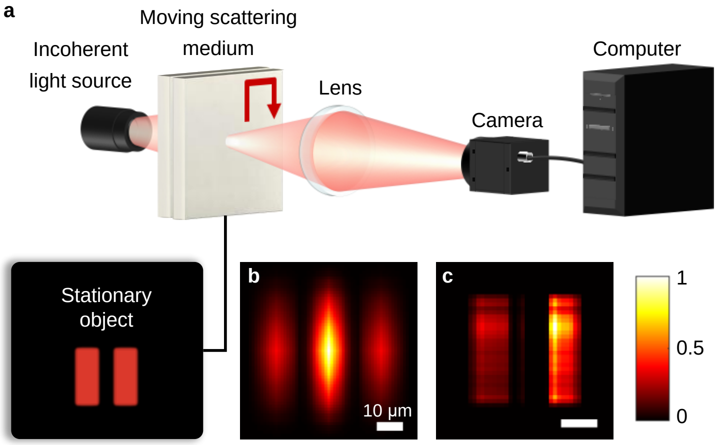

Supplementary Fig. 9 Imaging of a stationary object hidden in a moving scattering medium. **a** Experimental setup. The hidden object consists of 2 transmissive lines (10 μm widths, 10 μm interval, 30 μm heights) on the negative resolution test targets. The parafilm is moved in a U-shaped trajectory in the plane perpendicular to the optical axis. **b** The constructed object's autocorrelation. **c** The reconstructed object image. Scale bars: 10 μm.

Supplementary Note 9: Imaging of moving objects around corners

NLOS imaging allows observation of hidden objects around a corner using diffuse reflection of visible surfaces and has many potential applications. Here, we experimentally demonstrate that imaging of a moving object around a corner can be achieved even if a scattering medium is located between the object and the visible surface. As shown in Supplementary Fig. 10a, a transmissive object (three lines with 25 μm widths, 25 μm intervals and 125 μm heights) was illuminated via a single-colour LED and controlled by a two-axis motorized precision translation stage. The transmitted object light was scattered by a ground glass diffuser (DG20-220) and then diffusely reflected by a visible surface (white polystyrene slab). The imaging system consisting of a lens and a camera recorded a series of magnified speckle images from the visible surface during the object's motion. Through the overlapping speckle correlation algorithm, the object's autocorrelation is constructed from the noninvasively recorded speckle images (Supplementary Fig. 10b). Then, the image of the moving object hidden behind the occluder was reconstructed through a phase-retrieval algorithm (Supplementary Fig. 10c). As demonstrated, speckle kinetography can provide novel concepts for NLOS imaging in scattering environments. In addition, this experiment indirectly demonstrates that speckle kinetography is also effective for imaging through multilayer scattering media.

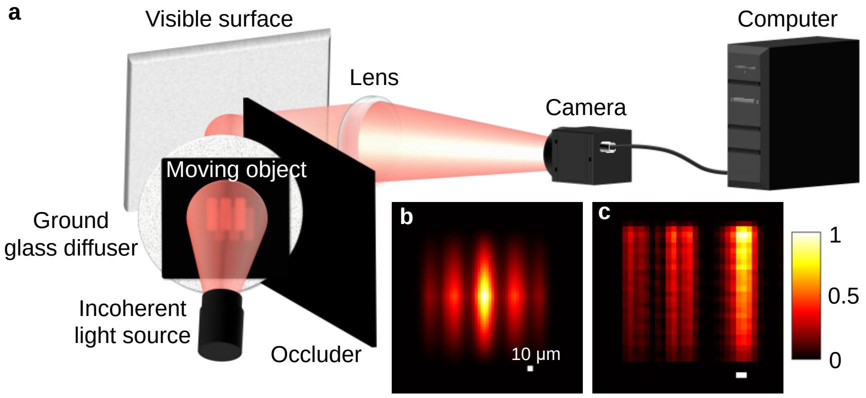

Supplementary Fig. 10 NLOS imaging of a moving object around corners. a Experimental setup. A moving object, which consists of 3 transmissive lines (25 μm widths, 25 μm intervals, 125 μm heights) on the negative resolution test target, is illuminated via an incoherent light source and hidden behind an occluder. The transmitted object light is scattered by a ground glass diffuser and then diffusely reflected by a visible surface. The speckles on the visible surface are recorded by using an imaging system consisting of a lens and a camera. **b** The constructed object's autocorrelation. **c** The reconstructed object image. Scale bars: 10 μm.

Supplementary Note 10: Scattering properties of the scattering samples

In the first two experiments, the scattering samples were parafilm of 6, 14 and 22 layers with a thickness of approximately 125 μm per layer. The parafilm is elastic and deformable with scattering properties similar to biological tissues. These samples are shown in Supplementary Fig. 11a and are split in half to show the depth of the object inside them. In the third experiment, the scattering sample was a chicken breast with a thickness of ~700 μm. As the scattering mean free path, l_s, varies with wavelength, we need to separately measure l_s of the parafilm and chicken breasts under single-colour LED (625 ± 8.5 nm), white light (260 nm to 2500 nm) and fluorescence (585 ± 20 nm) illumination.

First, we used the experimental system[1,2] shown in Supplementary Fig. 11b to measure the l_s of parafilm at a wavelength of 625 ± 8.5 nm. A light beam from the LED was collimated by a lens and transmitted through two irises with diameters of 8 mm. The incident intensity I_i was measured by a power metre 60 cm away from iris 2. Then, a parafilm was placed between these two irises, and the intensity of the ballistic light I_b was measured via a power metre. Since the exponential decay of ballistic photons follows the Beer–Lambert law, the transmittance T of a scattering sample follows the relation as shown below[1-4]:

$$T = \frac{I_b}{I_i} = \exp\left(-\mu_s L\right) \tag{1}$$

where L is the sample thickness and $\mu_s = 1/l_s$ is the scattering coefficient. A series of T values were calculated from the experimentally measured intensities by successively changing the thickness L of the parafilm from 1 to 18 layers (blue small circles in Supplementary Fig. 11c). By fitting the experimental data according to Eq. (1), shown as the blue curve in Supplementary Figs. 11c, we obtained that $\mu_s \approx 6.13$ mm^{-1} and thus $l_s \approx 163.1$ μm. Therefore, the parafilm of 6, 14 and 22 layers corresponded to $4.6l_s$, $10.7l_s$ and $16.9l_s$, respectively, at a wavelength of 625 ± 8.5 nm.

Second, we used a research arc lamp source as the light source to measure l_s of the parafilm under white light illumination. Since the power metre was only suitable for narrowband light, l_s could not be measured directly with white light. Therefore, we placed four bandpass filters (460 nm, 530 nm, 633 nm and 670 nm with 10 nm bandwidths) before iris 1 in turn. Then, we obtained four corresponding μ_s values in the

same way as above (blue small circles in Supplementary Fig. 11d). Since the wavelength ranges of the camera response and the white light source's flat irradiance were both 350 nm to 800 nm, we selected to calculate μ_s values at wavelengths within this range. By fitting the calculated experimental data μ_s according to the following[2]:

$$\mu_s = a\lambda^b \tag{2}$$

where a and b are constants; we obtained μ_s values at wavelengths from 350 nm to 800 nm (blue curve in Supplementary Fig. 11d). Then, the final scattering coefficient of parafilm under white light was determined to be $\mu_s \approx 6.15$ mm^{-1} by calculating the average of all the obtained μ_s values. Thus, $l_s \approx 162.6$ μm. Therefore, the parafilm of 6, 14 and 22 layers corresponded to $4.6l_s$, $10.8l_s$ and $16.9l_s$, respectively, under broadband illumination.

Third, we placed the bandpass filter (585 ± 20 nm) before iris 1 under white light illumination to measure the l_s of the chicken breast at a wavelength of 585 ± 20 nm. Similarly, a series of T values were calculated by changing the thickness L of the chicken breast from 0.70 mm to 4.92 mm (blue small circles in Supplementary Fig. 11e). By fitting the experimental data according to Eq. (1), we determined that $\mu_s \approx 5.1$ mm^{-1} and $l_s \approx 196.1$ μm. Thus, the chicken breast with 700 μm thickness corresponded to approximately $3.6l_s$ at this wavelength.

The scattering coefficient of polyethylene foams (Supplementary Fig. 11f) was also measured with the experiment system shown in Supplementary Fig. 11b. The polyethylene foam was a homogeneous static isotropic scattering sample. As the thickness of the polyethylene foam was fixed, a group of scattering coefficients were measured under various incident light intensities. Then, the scattering coefficient was calculated as $\mu_s \approx 0.34$ mm^{-1} by averaging. Therefore, the polyethylene foams with 15 mm and 20 mm thicknesses corresponded to approximately $5.1l_s$ and $6.8l_s$, respectively.

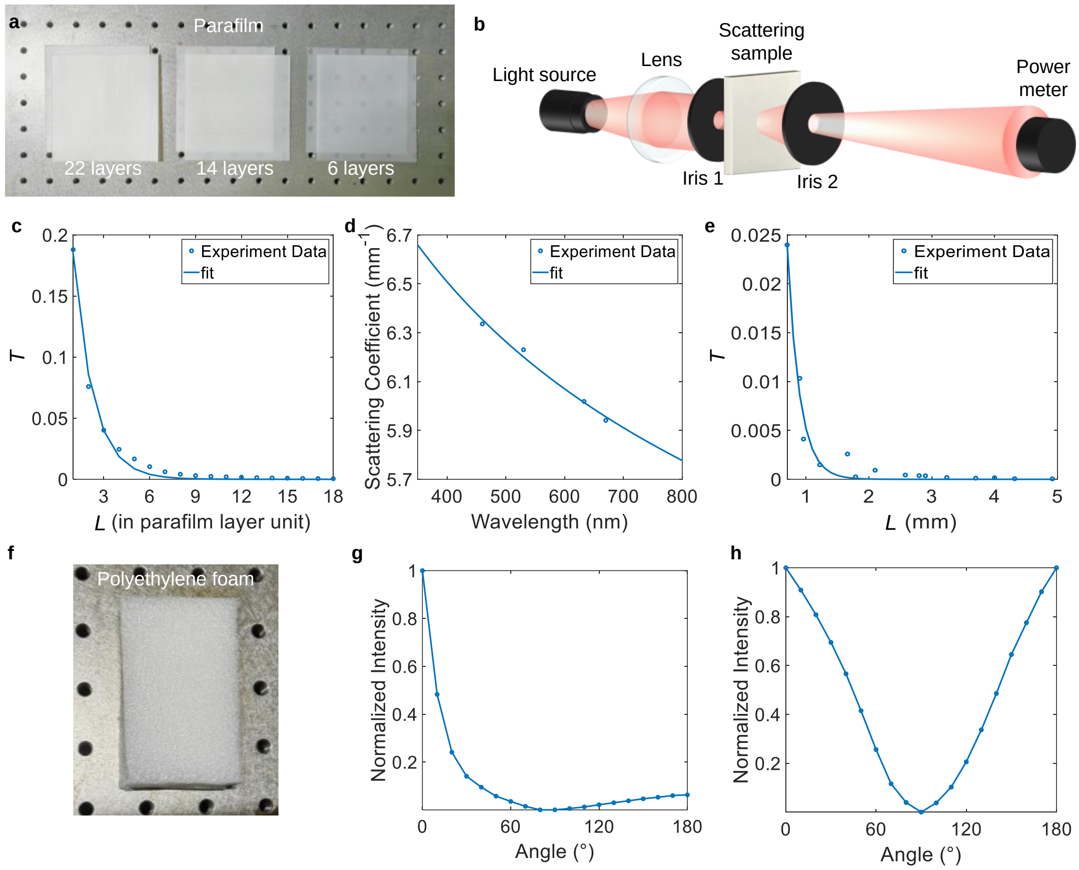

Supplementary Fig. 11 Scattering properties of the scattering samples. a The parafilm of 22, 14 and 6 layers. **b** The experimental setup to measure the scattering mean free path. **c** Measurement of the scattering mean free path of parafilm at 625 ± 8.5 nm. **d** Measurements of the scattering coefficients of parafilm at wavelengths from 350 nm to 800 nm. **e** Measurement of the scattering mean free path of chicken breasts at 585 ± 20 nm. **f** The photograph of the 15 mm thick polyethylene foams. **g** The measured angular distributions of the scattered light from the parafilm. **h** The measured angular distributions of the scattered light from the polyethylene foams.

The anisotropy coefficient g of the parafilm and the polyethylene foams were determined by measuring the angular distributions of scattered light[5,6]. The measured angular distributions of the scattered light of the parafilm and the polyethylene foams at a wavelength of 625 ± 8.5 nm are shown in Supplementary Figs. 11g and 11h, respectively. On this basis, the anisotropy coefficient of the parafilm and the polyethylene foams were calculated as $g \approx 0.69$ and $g \approx 0.02$, respectively. By eliminating the anisotropic effects, the parafilm of 6, 14, 22 and 26 layers corresponded to $1.4l_t$, $3.3l_t$, $5.2l_t$ and $6.2l_t$, respectively, at a wavelength of 625 ± 8.5 nm. And the polyethylene foams with 15 mm and 20 mm thicknesses corresponded to approximately $5.0l_s$ and $6.7l_s$, respectively.

Supplementary Note 11: Generation of envelope and speckle

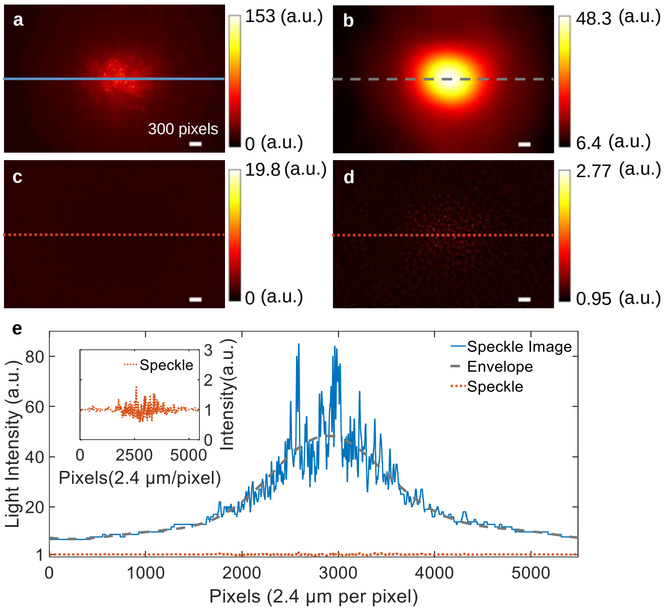

Supplementary Fig. 12 Generation of envelope and speckle from a speckle image. a A speckle image. **b** The low-pass filtered envelope. **c** The spatially normalized speckle *S*. **d** The spatially normalized speckle *S* shown under a colour bar of a small range. **e** The values along the middle row of images in blue (**a**), grey (**b**), and red (**c** and **d**). The solid blue curve represents the distribution of the speckle image (**a**). The grey dashed curve represents the distribution of the envelope (**b**). The red dotted curve represents the distribution of the spatially normalized speckle *S* (**c** and **d**). The inset is a closer look at the speckle *S* in (**e**).

The generating process of the envelope and the speckle from a speckle image is shown in Supplementary Fig. 12. As shown in Supplementary Fig. 12a, the values of a speckle image are in the range of 0 to 153. The values along the middle row (solid blue line in Supplementary Fig. 12a) are also shown as the solid blue curve in Supplementary Fig. 12e. The low-pass filtered envelope is shown in Supplementary Fig. 12b. The values along the middle row (grey dashed line in Supplementary Fig. 12b) are also shown as the grey dashed curve in Supplementary Fig. 12e. It can be seen that the distribution of the envelope follows the general distribution of the speckle image, but the high-frequency details have been filtered. The values at the edges of the speckle image and the envelope are approximately equal and positive. Therefore, by dividing the speckle image with its envelope, the spatially normalized speckle *S* (Supplementary Fig. 12c) fluctuates around 1, which can be more directly observed in Supplementary Fig. 12e (red dotted curves in the figure and the inset). In order to show the normalized speckle *S* in Supplementary Fig. 12c more clearly, the colour bar range is changed from 0~19.8 to 0.95~2.77 (Supplementary Fig. 12d).

Supplementary Note 12: The dispersion of white light in direct imaging experiments

The lens used in all experiments is an achromatic lens with anti-reflective coating at 400-750 nm. Its focal length specification wavelengths are 486.1 nm, 587.6 nm and 656.3 nm. However, the spectral range of the white light source (Xenon Arc Lamp, Newport, 66907-150XF-R1, Model: 6255) is about 260-2500 nm, and the response range of the camera (CMOS USB3.0 Rolling Shutter Camera, HuaTeng Vision, Mono Model: HT-SUA2000M-T) is about 350-1000 nm. The spectral irradiance of the white light source is available in Fig. 4 at Page 23 of its product manual[7], and the response of the camera is available at Page 1 of its product manual, which can be downloaded from the product webpage[8] (click 'Download Now' at the bottom of the webpage). These wider wavelength ranges cause the direct imaging results to suffer from broadening problem. Nevertheless, although using the same lens, light source and camera as direct imaging, the scattering imaging results of speckle kinetography are not broadened by dispersion. These experimental results of imaging with and without scattering media can be seen as a strong proof that the speckle kinetography can avoid the imaging broadening problem.

Supplementary Note 13: The phase-retrieval algorithm

The iterative Fienup-type phase-retrieval algorithm we used follows the work of Katz et al.[9]. The algorithm flow chart is given in Supplementary Box 1. This modified Gerchberg-Saxton algorithm starts with an initial guess for the object pattern $o_1(x, y)$. This initial guess, chosen as a random pattern, is entered to the algorithm that performs the following 4 steps at its ith iteration:

$$\begin{aligned} O_i\left(k_x,k_y\right) &= \mathrm{FT}\left\{o_i\left(x,y\right)\right\} \\ \theta_i\left(k_x,k_y\right) &= \arg\left\{O_i\left(k_x,k_y\right)\right\} \\ O_i^{'}\left(k_x,k_y\right) &= \sqrt{\left|\mathrm{FT}\left\{C\left(x,y\right)\right\}\right|}\,e^{j\theta_i\left(k_x,k_y\right)} \\ o_i^{'}\left(x,y\right) &= \mathrm{FT}^{-1}\left\{O_i^{'}\left(k_x,k_y\right)\right\} \end{aligned} \tag{3}$$

where the $C(x, y)$ in the 3rd step is the measured object's autocorrelation.

The input for the next (i+1) iteration, $o_{i+1}(x, y)$, is obtained from the output of the ith iteration, $o_i^{'}(x, y)$, by imposing physical constraints on the object image, which is real and non-negative in our implementation. Two types of implementations of these constraints are used in the algorithm[9-11], termed the Hybrid Input-Output (HIO) algorithm:

$$o_{i+1}\left(x,y\right)=\begin{cases} o_i^{'}\left(x,y\right) & \text{for}\left(x,y\right)\notin\Gamma \\ o_i\left(x,y\right)-\beta o_i^{'}\left(x,y\right) & \text{for}\left(x,y\right)\in\Gamma \end{cases} \tag{4}$$

and the Error reduction algorithm:

$$o_{i+1}\left(x,y\right)=\begin{cases} o_i^{'}\left(x,y\right) & \text{for}\left(x,y\right)\notin\Gamma \\ 0 & \text{for}\left(x,y\right)\in\Gamma \end{cases} \tag{5}$$

where Γ is the set of all points (x, y) on $o_i^{'}(x, y)$ that violate the physical constraints, and β is a feedback parameter that control the convergence properties of the algorithm.

Following Katz et al.[9], a few thousand iterations of the HIO algorithm were ran with a decreasing beta factor from β = 2 to β = 0, in steps of 0.04. For each β value, 40 iterations of the algorithm were performed. The result of the HIO algorithm was fed as an input to additional 40 iterations of the Error reduction algorithm to obtain final result.

To assure faithful reconstruction of each image, several different runs of the algorithm (from 10 up to 60, typically 20) were performed with different random initial conditions, and the reconstruction having the closest Fourier spectrum to the measured autocorrelation Fourier transform (lowest mean-square-error) was chosen as the final reconstructed result.

Supplementary Box 1 ❙ The iterative phase-retrieval algorithm

1. Initialize:
Choose a random pattern as an initial guess for the object pattern $o_1(x, y)$.

2. Feedforward: For every entered object pattern $o_i(x, y)$, perform the following steps:
2a. Calculate the 2D Fourier Transform of $o_i(x, y)$ to obtain $O_i(k_x, k_y)$.
2b. Calculate the argument of $O_i(k_x, k_y)$ to obtain $\theta_i(k_x, k_y)$.
2c. Combine $\theta_i(k_x, k_y)$ with the measured object's autocorrelation $C(x, y)$ to obtain

$$O_i^{'}\left(k_x, k_y\right) = \sqrt{\left|FT\left\{C\left(x, y\right)\right\}\right|}e^{j\theta_i\left(k_x, k_y\right)}.$$

2d. Calculate the inverse 2D Fourier transform of $O_i^{'}(k_x, k_y)$ to obtain $o_i^{'}(x, y)$.

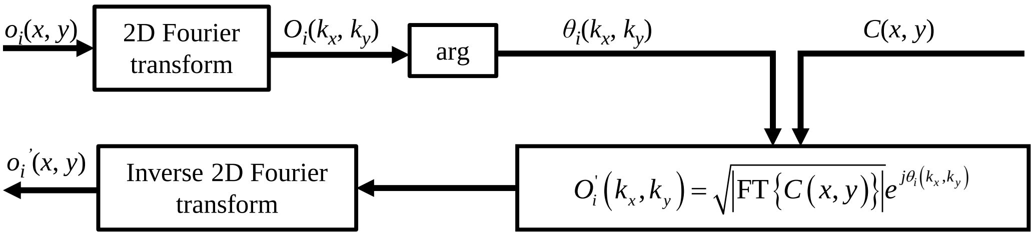

3. Constraints: Impose physical constraints on the object image $o_i^{'}(x, y)$:
3a. Perform the Hybrid Input-Output (HIO) algorithm on $o_i^{'}(x, y)$ with a decreasing beta factor from $\beta = 2$ to $\beta = 0$, in steps of 0.04, and 40 iterations for each β value.
3b. Perform the Error reduction algorithm on the result of step 3a. with 40 iterations.
3c. Obtain $o_{i+1}(x, y)$ from step 3b. to use as the starting point for the $(i+1)^{\text{th}}$ iteration.

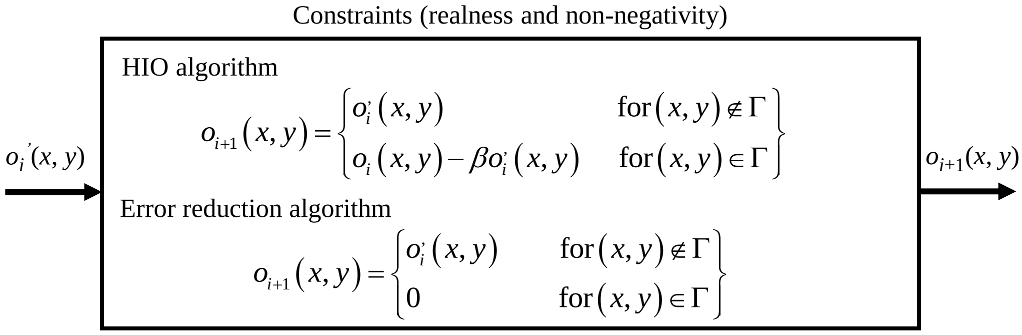

4. Repeat:
4a. Repeat step 2. and 3. with 20 iterations.
4b. Choose the reconstruction having the closest Fourier spectrum to the measured autocorrelation Fourier transform (lowest mean-square-error) as the final reconstructed result.

Supplementary References

1. Lyu, M., Wang, H., Li, G., Zheng, S. & Situ, G. Learning-based lensless imaging through optically thick scattering media. *Adv. Photonics* **1**, 036002 (2019).
2. Michels, R., Foschum, F. & Kienle, A. Optical properties of fat emulsions. *Opt. Express* **16**, 5907-5925 (2008).
3. Skarsoulis, K., Kakkava, E. & Psaltis, D. Predicting optical transmission through complex scattering media from reflection patterns with deep neural networks. *Opt. Commun.* **492**, 126968 (2021).
4. Boniface, A., Blochet, B., Dong, J. & Gigan, S. Noninvasive light focusing in scattering media using speckle variance optimization. *Optica* **6**, 1381-1385 (2019).
5. Fukutomi, D., Ishii, K. & Awazu, K. Determination of the scattering coefficient of biological tissue considering the wavelength and absorption dependence of the anisotropy factor. *Opt. Rev.* **23**, 291-298 (2016).
6. Fernandez-Oliveras, A., Rubiño, M. & Perez, M. M. Scattering anisotropy measurements in dental tissues and biomaterials. *J. Europ. Opt. Soc. Rap. Public.* **7**, 12016 (2012).
7. Newport Corporation. www.newport.com.cn/medias/sys_master/images/images/hfb/hdf/8797196451870/Light-Sources.pdf.
8. Shenzhen HuaTeng Vision Technology Co., Ltd. www.huatengvision.com/PrevNewsContentPage/4502578.
9. Katz, O., Heidmann, P., Fink, M. & Gigan, S. Non-invasive single-shot imaging through scattering layers and around corners via speckle correlations. *Nat. Photonics* **8**, 784-790 (2014).
10. Bertolotti, J. et al. Non-invasive imaging through opaque scattering layers. *Nature* **491**, 232-234 (2012).
11. Fienup, J. R. Phase retrieval algorithms: a comparison. *Appl. Opt.* **21**, 2758-2769 (1982).
