# Supplementary material for: Overlapping speckle correlation algorithm for high-resolution imaging and tracking of objects in unknown scattering media
Source: Nat Commun. 2023 Nov 25;14:7742. doi: 10.1038/s41467-023-43674-5 (PMC10676403; doi:10.1038/s41467-023-43674-5)
Supplement: Supplementary file 3 — Description of Additional Supplementary Files [file 41467_2023_43674_MOESM3_ESM.pdf]

Description of Additional Supplementary Files

Supplementary Movie 1: The reconstructed video of a moving object hidden in a scattering sample under single-colour LED illumination. The object consisting of two 10 μm width lines is moved in a U shape during detection. The scattering sample is parafilm of 22 layers.

Supplementary Movie 2: The reconstructed video of a moving object hidden in a scattering sample under white light illumination. The object consisting of three 5 μm width lines is moved in a U shape during detection. The scattering sample is parafilm of 22 layers.

Supplementary Code 1: The code for the overlapping speckle correlation algorithm. This code can extract the object's overlapping and position information from the provided speckle images for autocorrelation construction. The phase retrieval algorithm is also contained in this code for imaging. The code has passed the test under Python3.8.
