# Supplementary figures and images for: Overlapping speckle correlation algorithm for high-resolution imaging and tracking of objects in unknown scattering media
Source: Nat Commun. 2023 Nov 25;14:7742. doi: 10.1038/s41467-023-43674-5 (PMC10676403; doi:10.1038/s41467-023-43674-5)

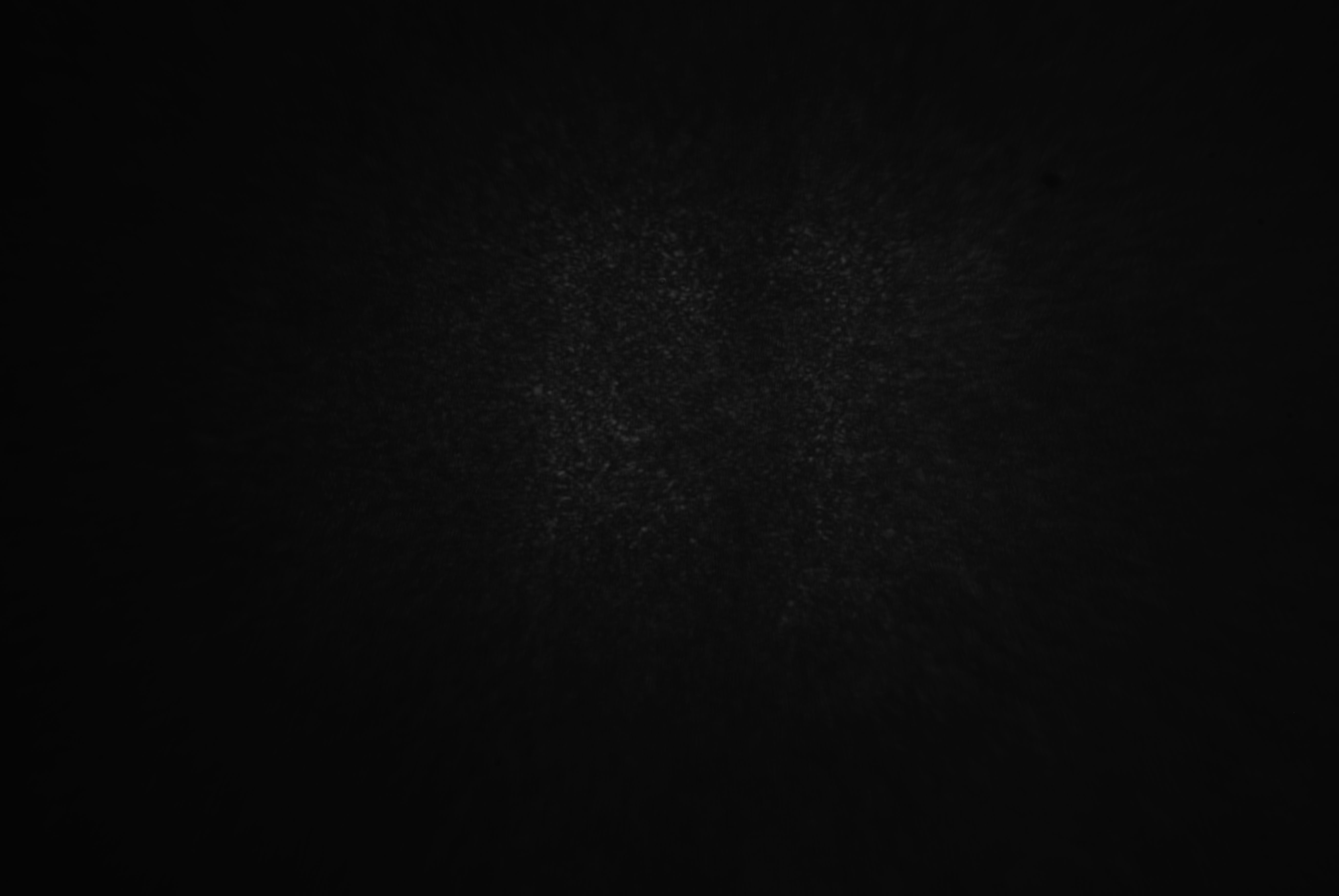

Supplement: Supplementary file 7 — Source Data [file 41467_2023_43674_MOESM7_ESM.zip › Source Data/Data 3/xx (30).JPG]

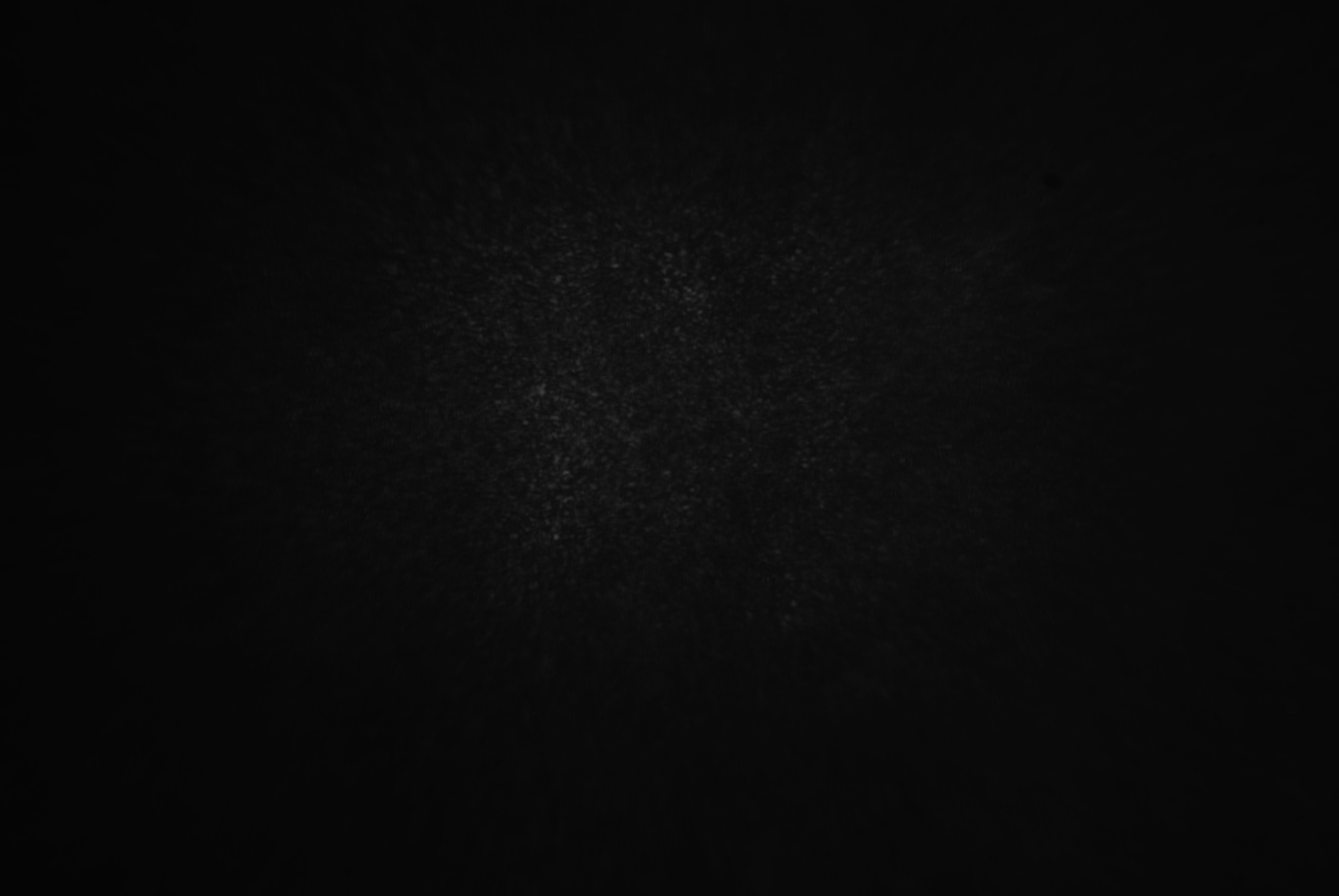

Supplement: Supplementary file 7 — Source Data [file 41467_2023_43674_MOESM7_ESM.zip › Source Data/Data 3/xx (1).JPG]

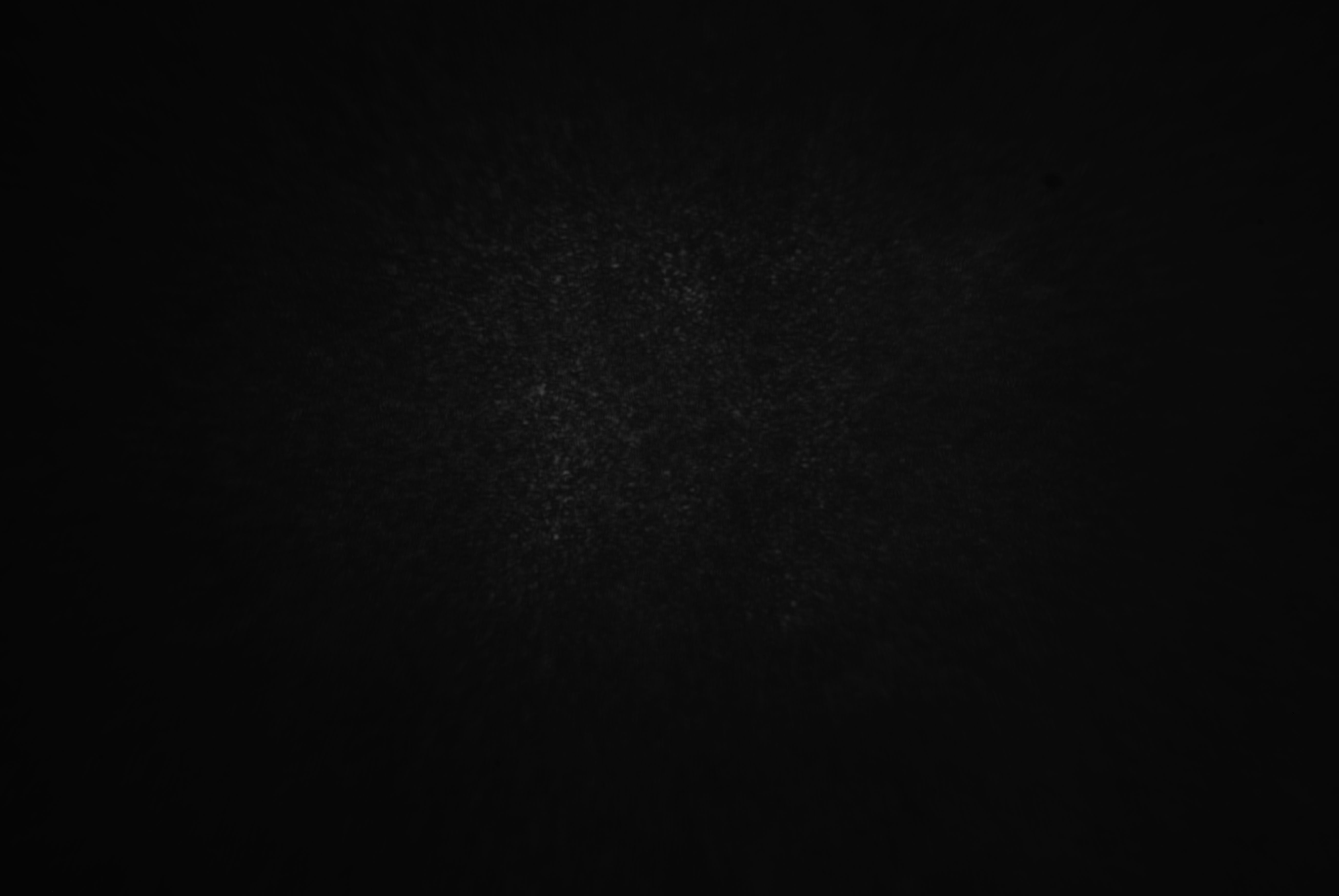

Supplement: Supplementary file 7 — Source Data [file 41467_2023_43674_MOESM7_ESM.zip › Source Data/Data 3/xx (2).JPG]

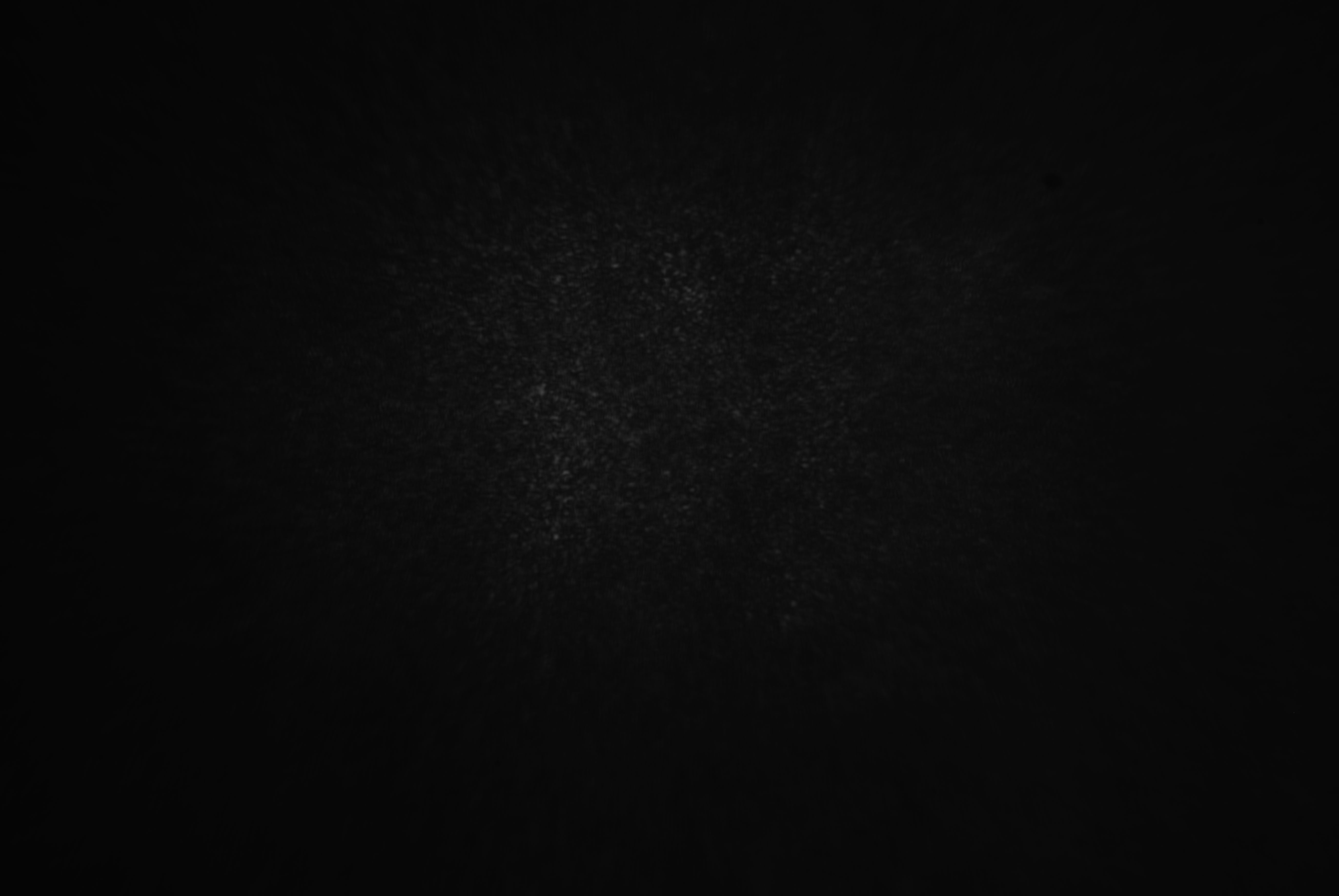

Supplement: Supplementary file 7 — Source Data [file 41467_2023_43674_MOESM7_ESM.zip › Source Data/Data 3/xx (3).JPG]

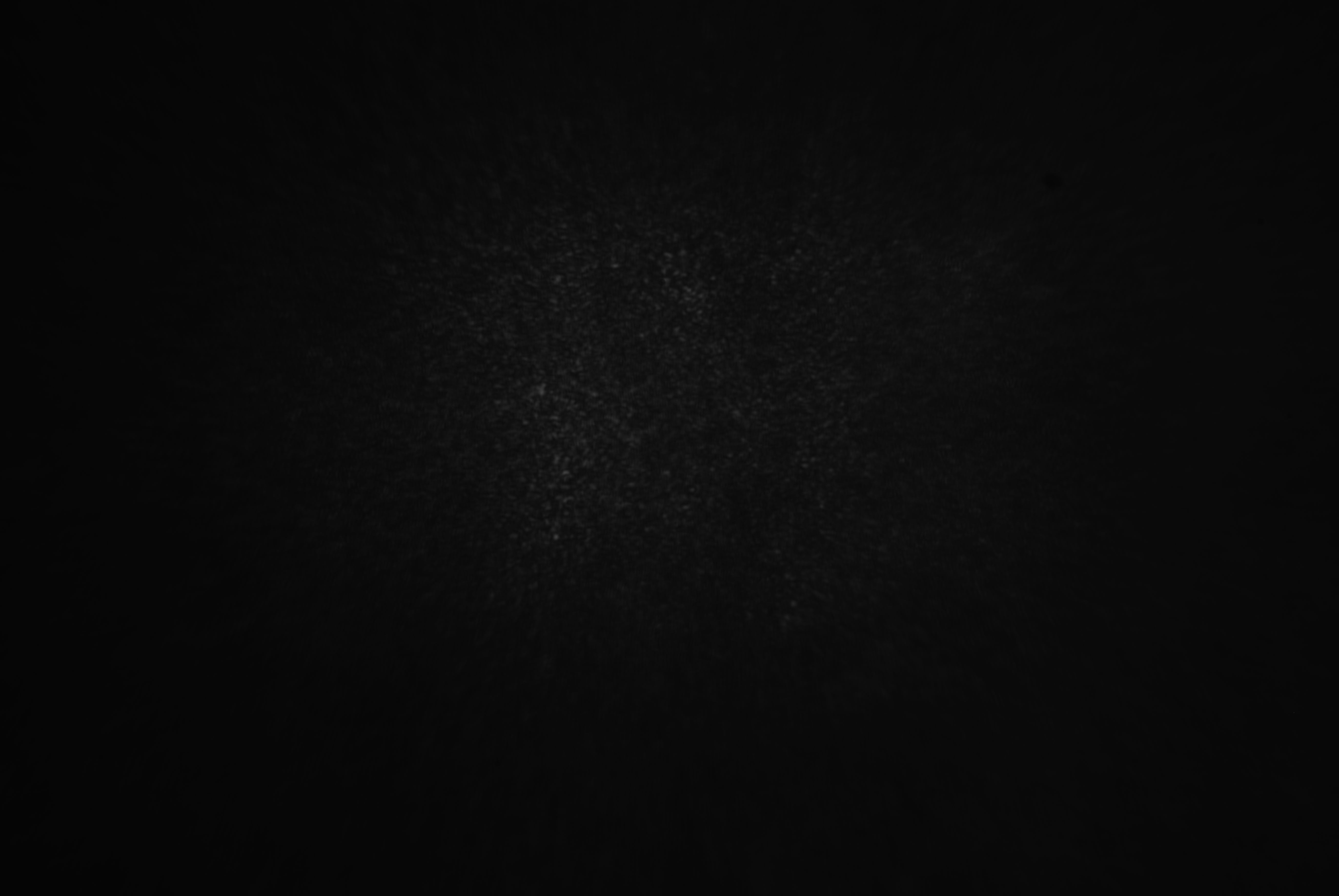

Supplement: Supplementary file 7 — Source Data [file 41467_2023_43674_MOESM7_ESM.zip › Source Data/Data 3/xx (4).JPG]

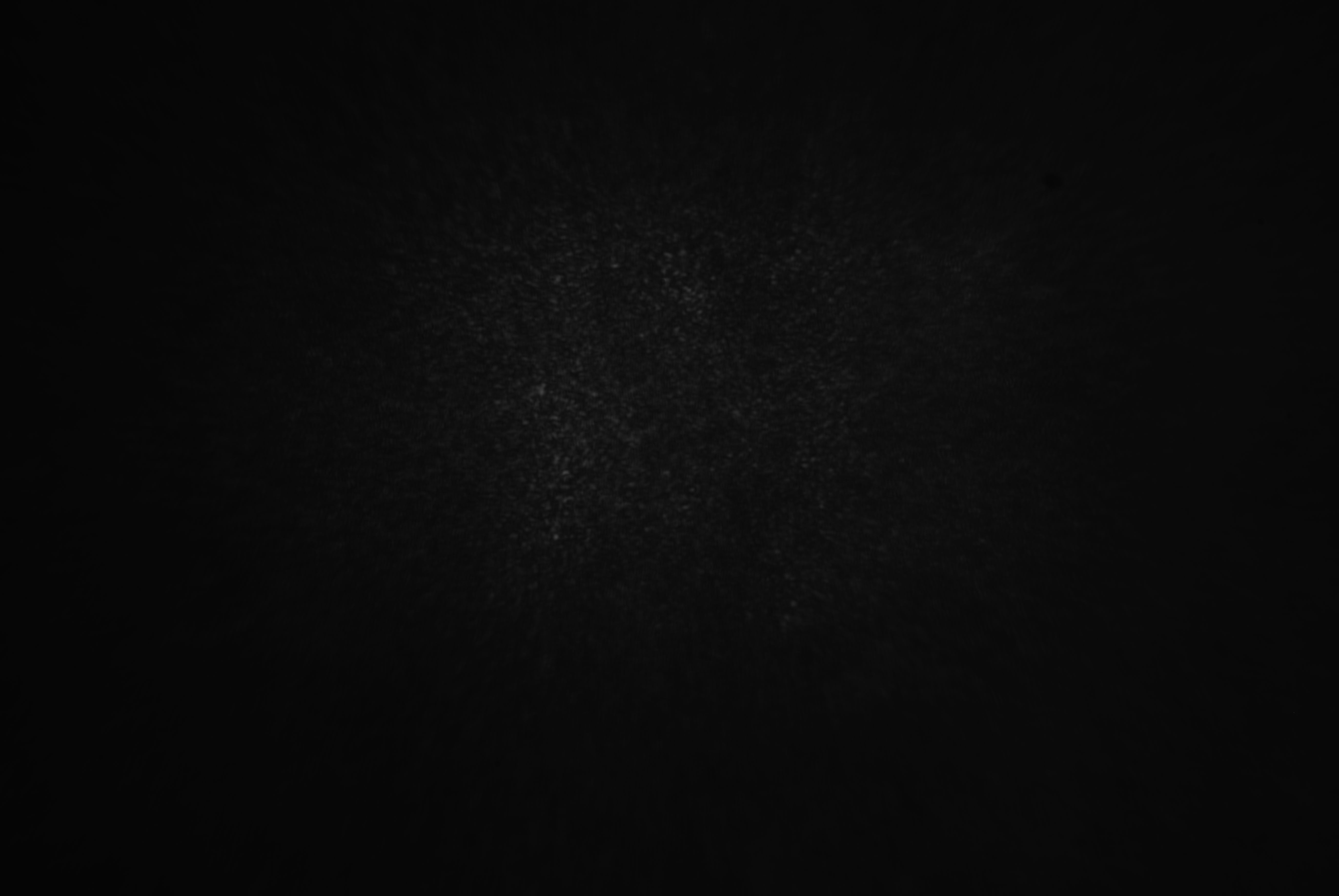

Supplement: Supplementary file 7 — Source Data [file 41467_2023_43674_MOESM7_ESM.zip › Source Data/Data 3/xx (5).JPG]

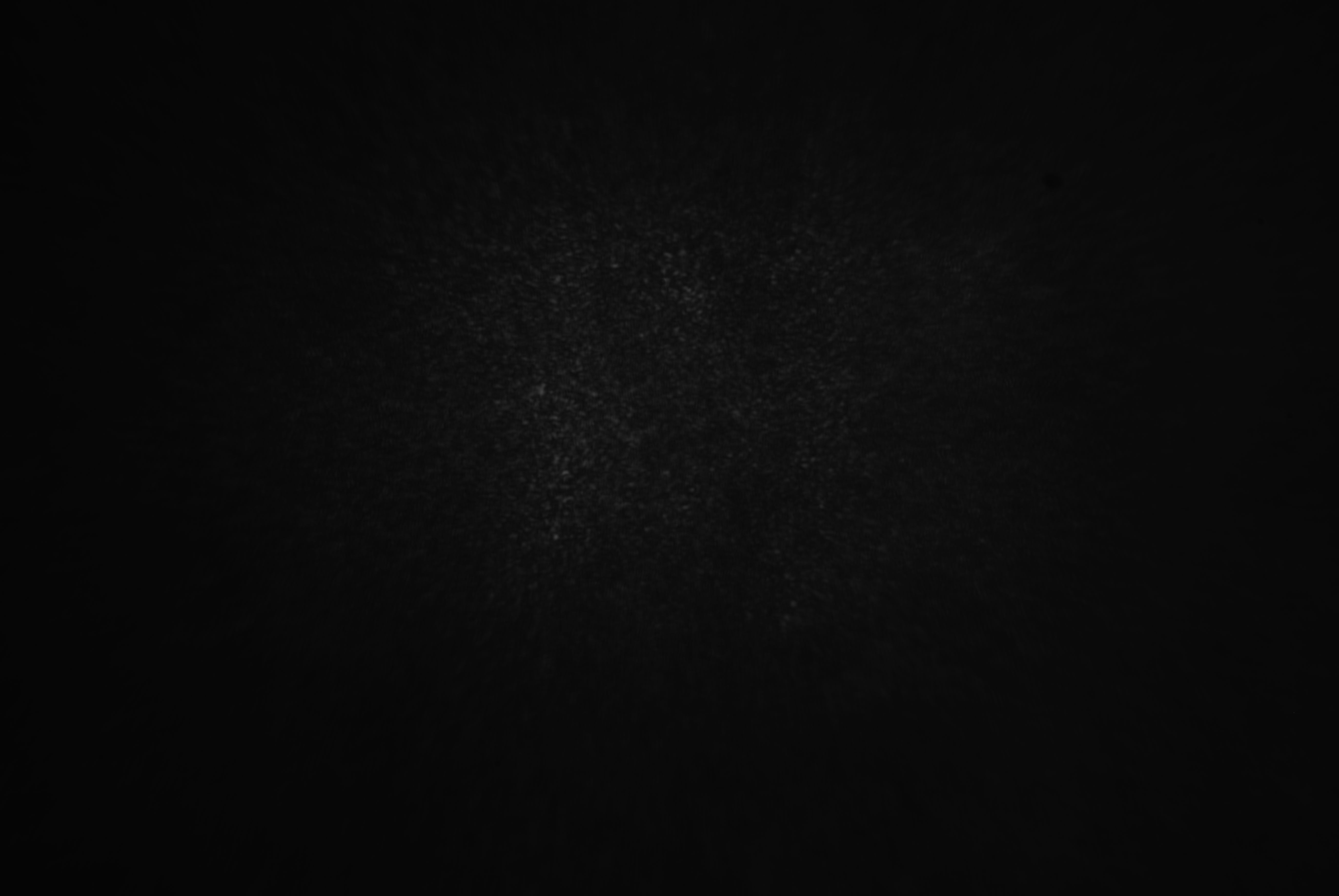

Supplement: Supplementary file 7 — Source Data [file 41467_2023_43674_MOESM7_ESM.zip › Source Data/Data 3/xx (6).JPG]

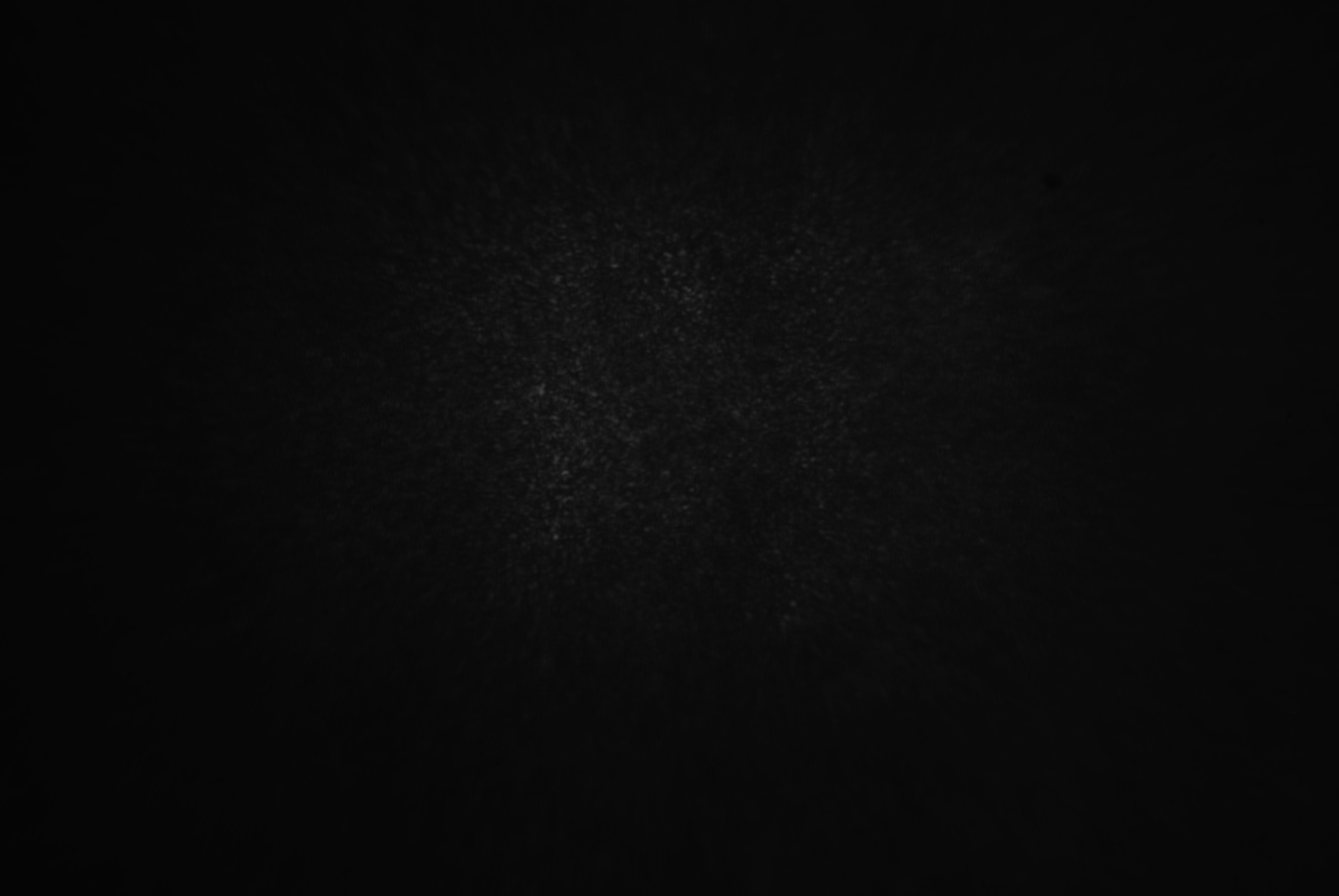

Supplement: Supplementary file 7 — Source Data [file 41467_2023_43674_MOESM7_ESM.zip › Source Data/Data 3/xx (7).JPG]

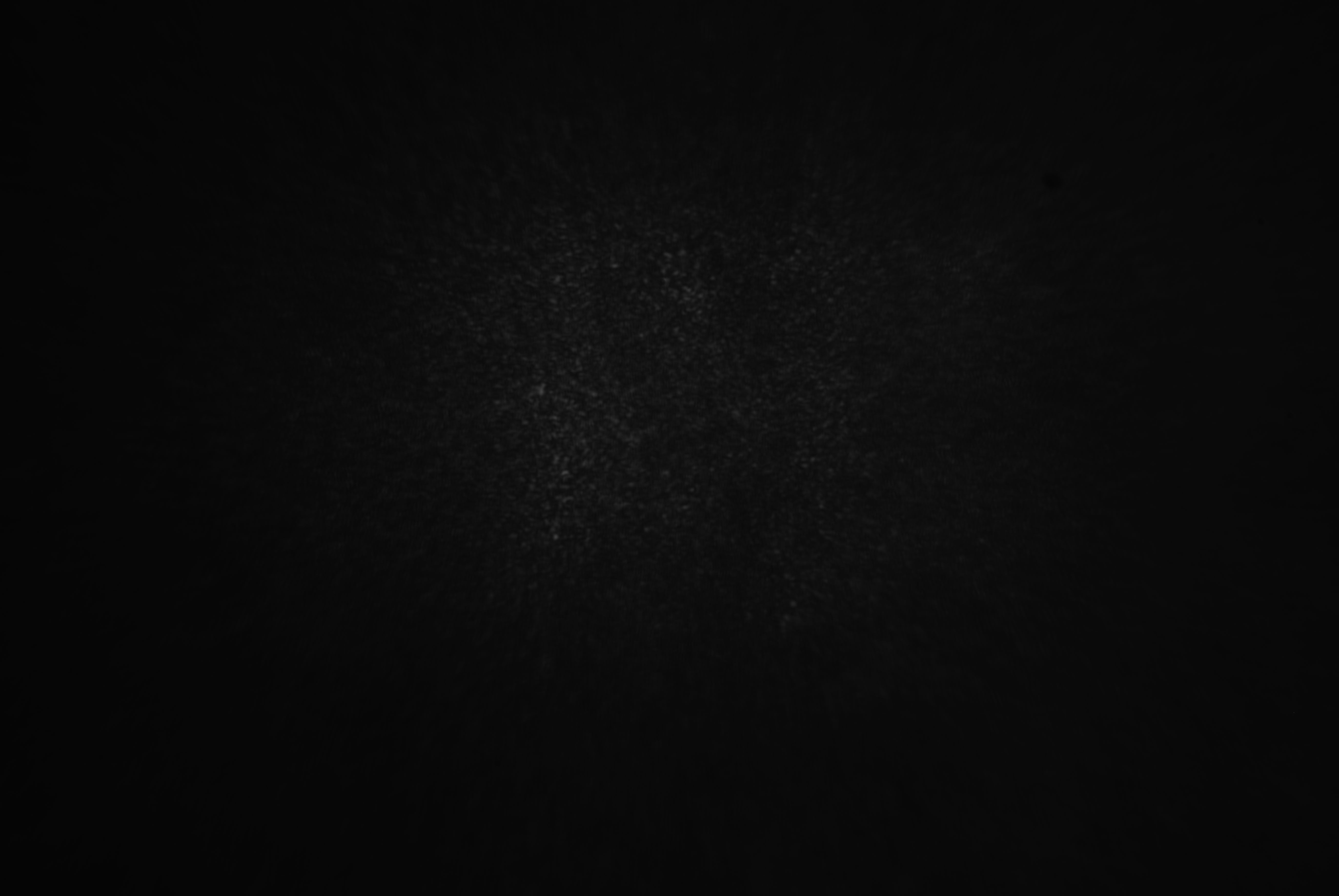

Supplement: Supplementary file 7 — Source Data [file 41467_2023_43674_MOESM7_ESM.zip › Source Data/Data 3/xx (8).JPG]

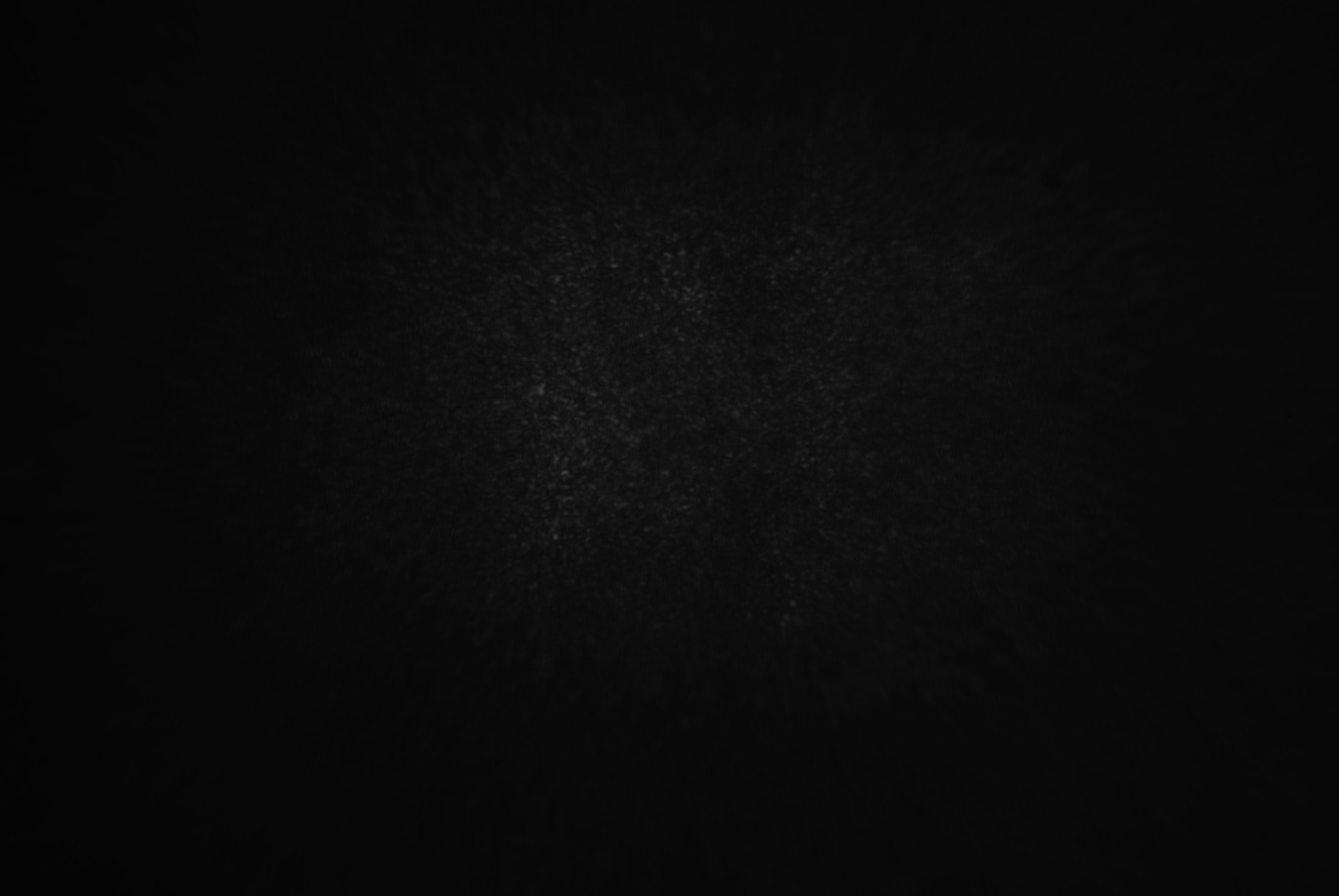

Supplement: Supplementary file 7 — Source Data [file 41467_2023_43674_MOESM7_ESM.zip › Source Data/Data 3/xx (9).JPG]

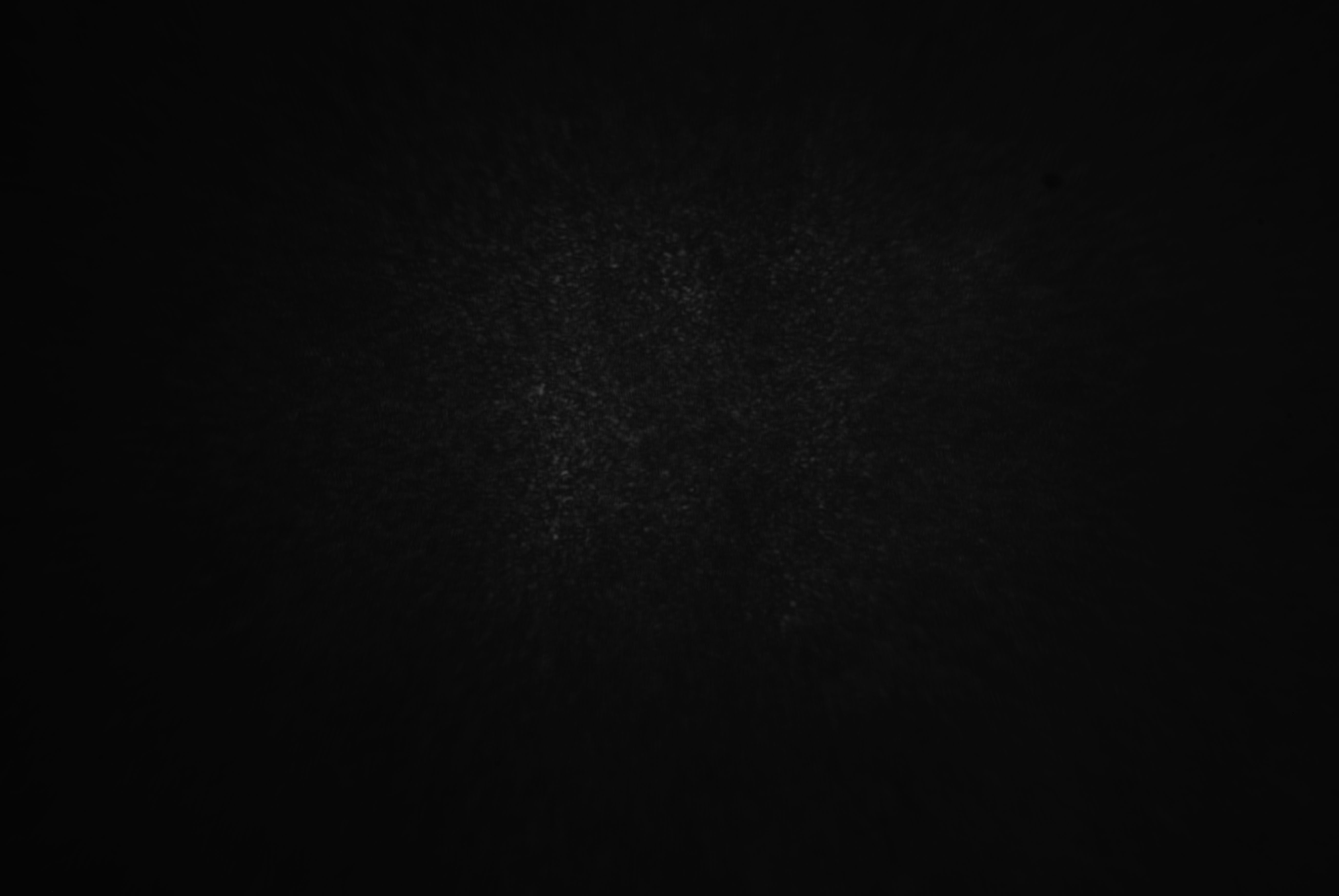

Supplement: Supplementary file 7 — Source Data [file 41467_2023_43674_MOESM7_ESM.zip › Source Data/Data 3/xx (10).JPG]

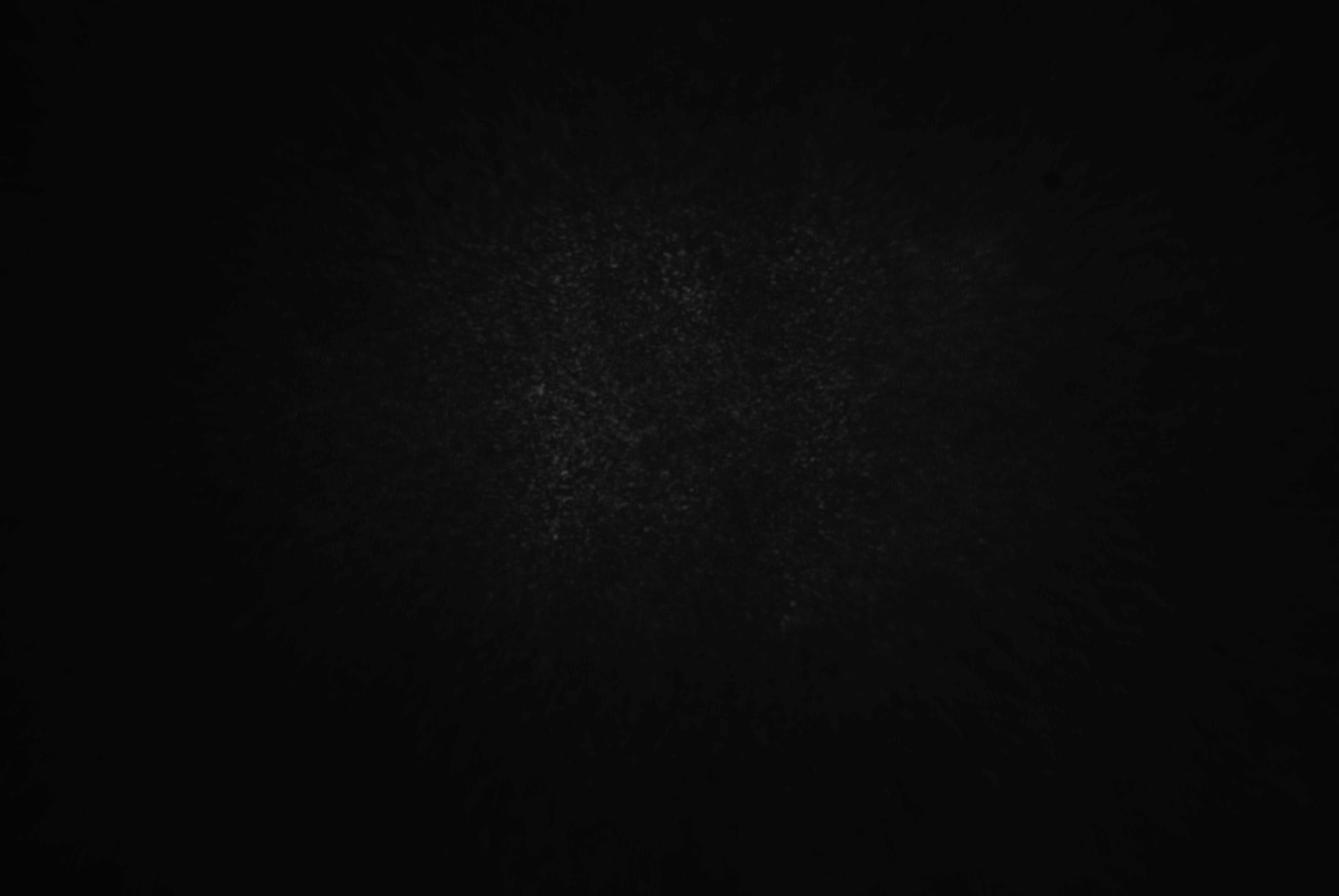

Supplement: Supplementary file 7 — Source Data [file 41467_2023_43674_MOESM7_ESM.zip › Source Data/Data 3/xx (11).JPG]

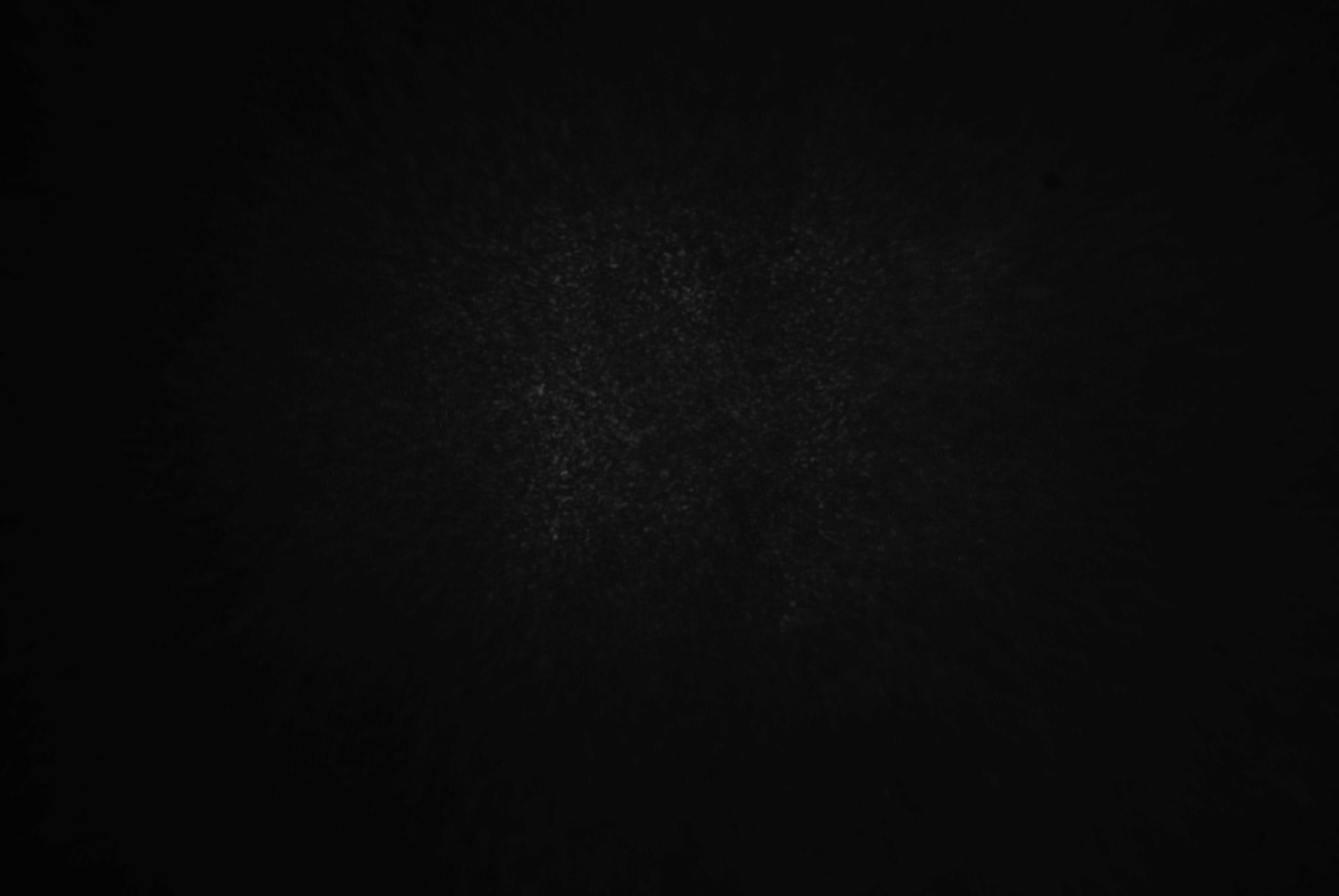

Supplement: Supplementary file 7 — Source Data [file 41467_2023_43674_MOESM7_ESM.zip › Source Data/Data 3/xx (12).JPG]

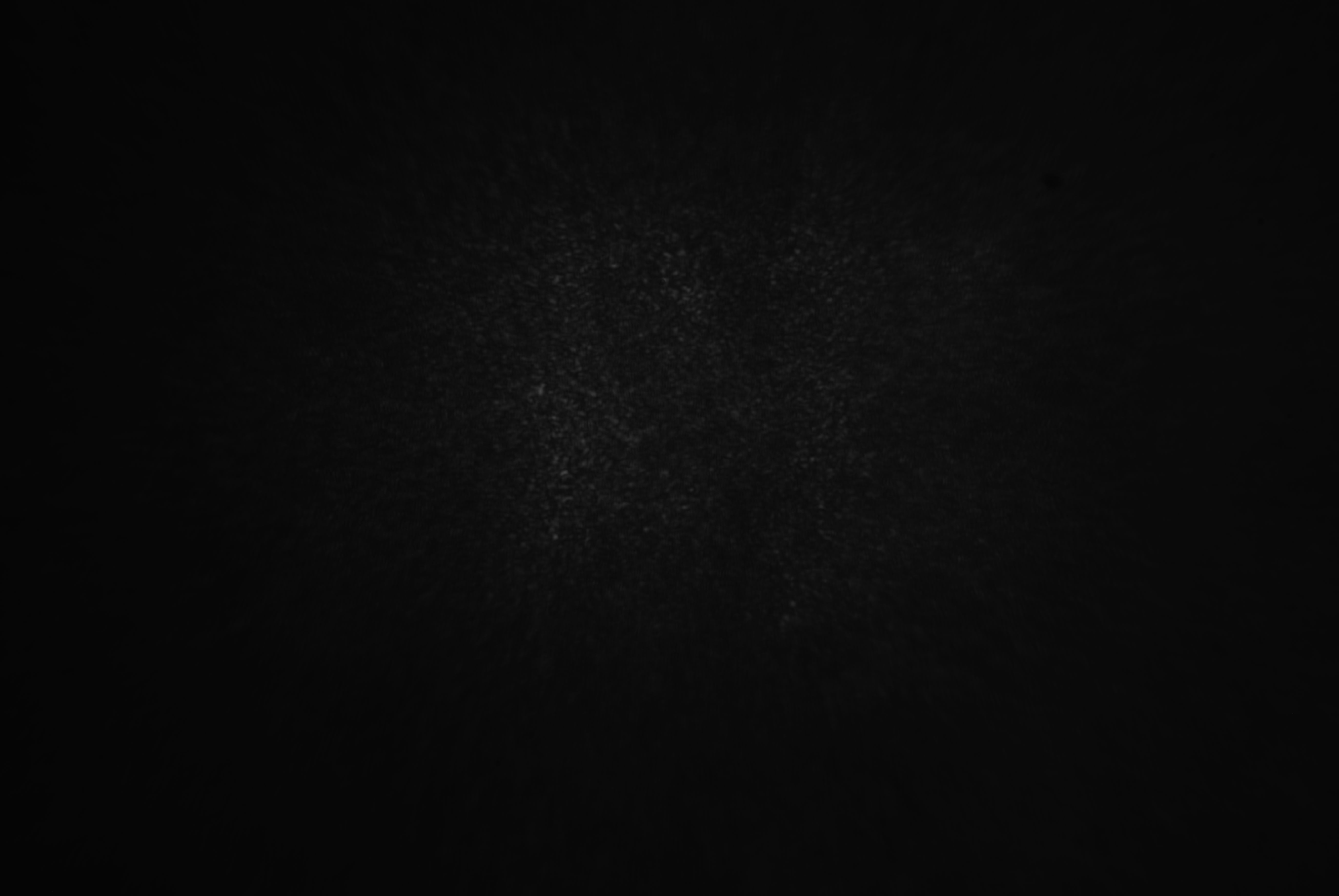

Supplement: Supplementary file 7 — Source Data [file 41467_2023_43674_MOESM7_ESM.zip › Source Data/Data 3/xx (13).JPG]

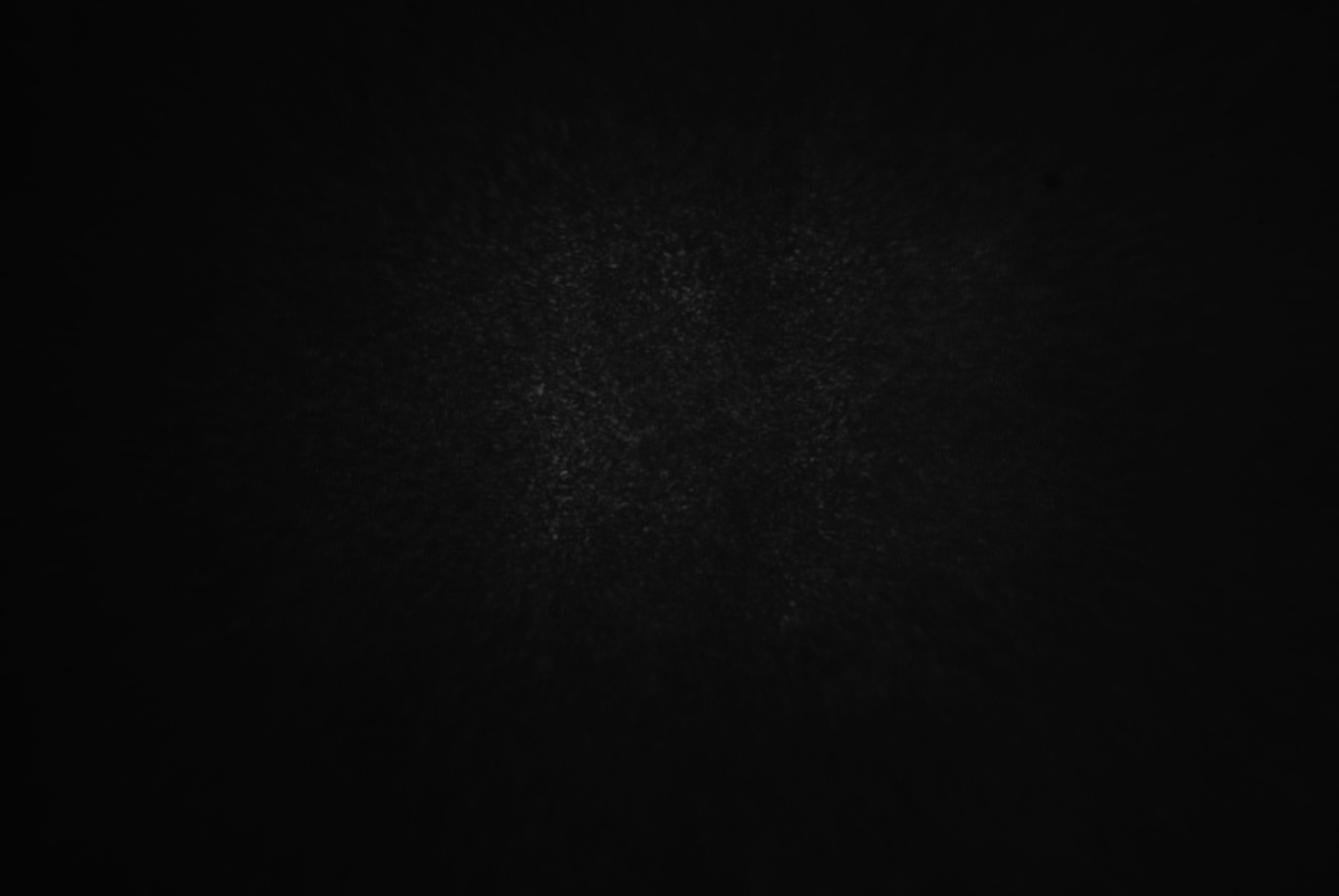

Supplement: Supplementary file 7 — Source Data [file 41467_2023_43674_MOESM7_ESM.zip › Source Data/Data 3/xx (14).JPG]

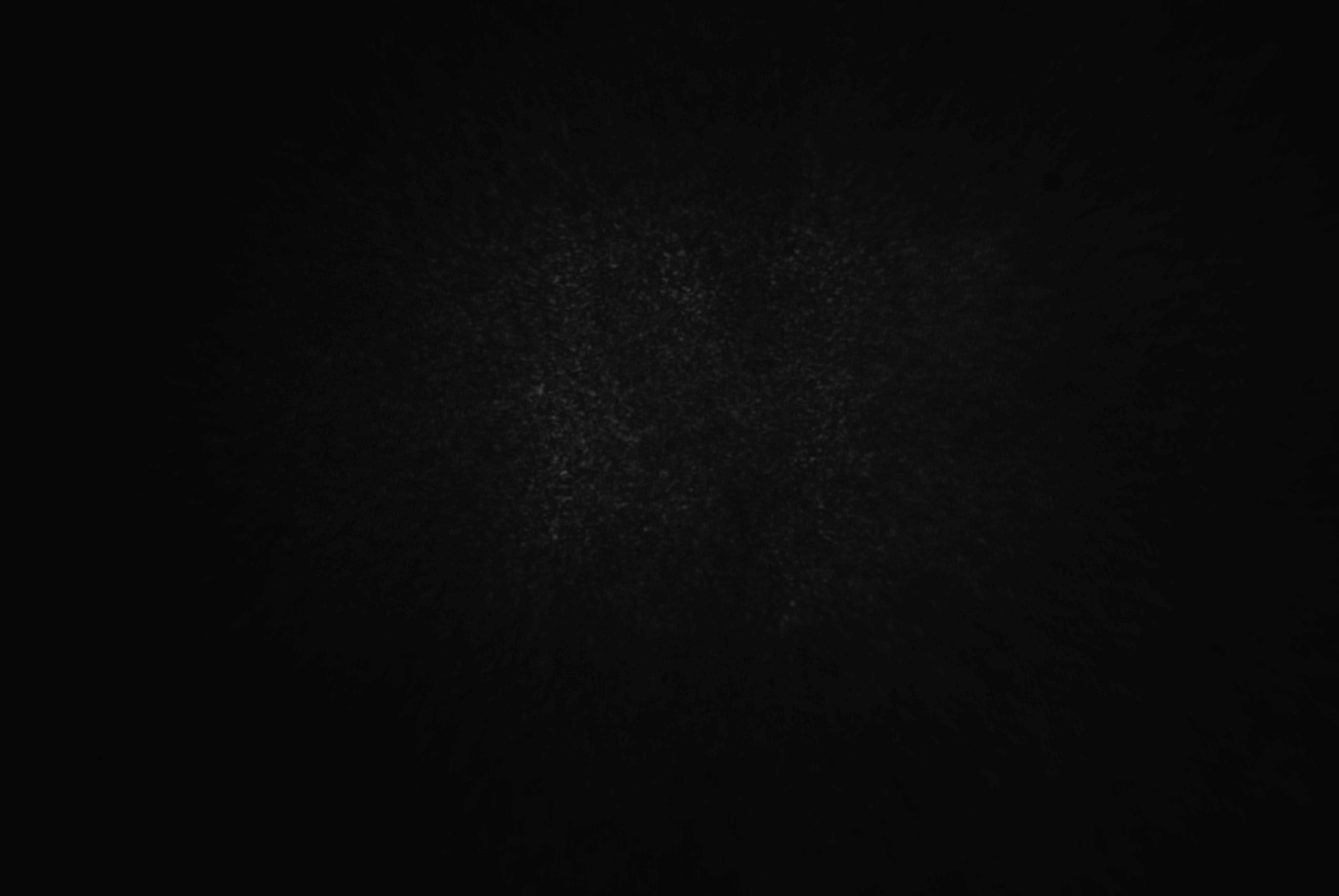

Supplement: Supplementary file 7 — Source Data [file 41467_2023_43674_MOESM7_ESM.zip › Source Data/Data 3/xx (15).JPG]

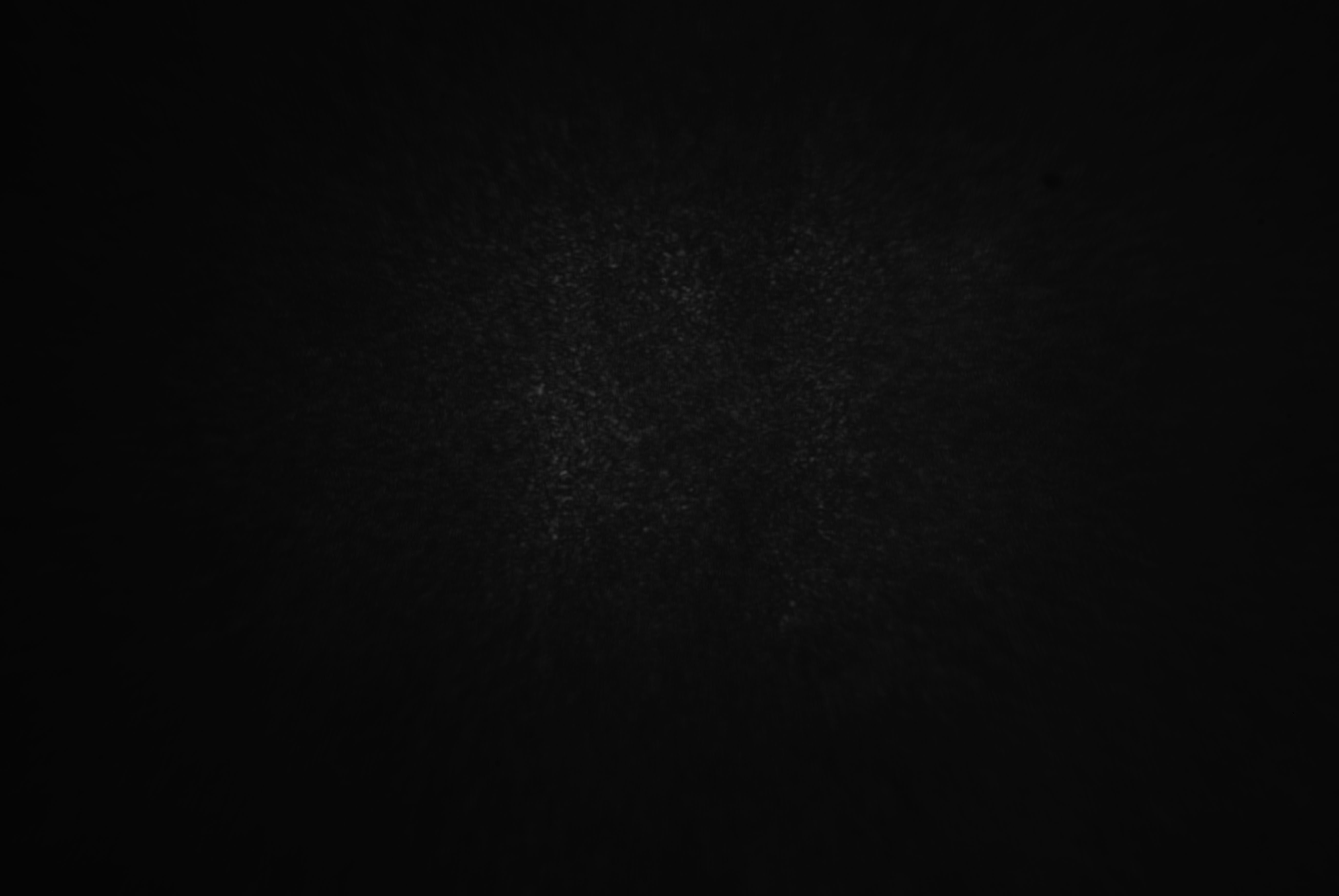

Supplement: Supplementary file 7 — Source Data [file 41467_2023_43674_MOESM7_ESM.zip › Source Data/Data 3/xx (16).JPG]

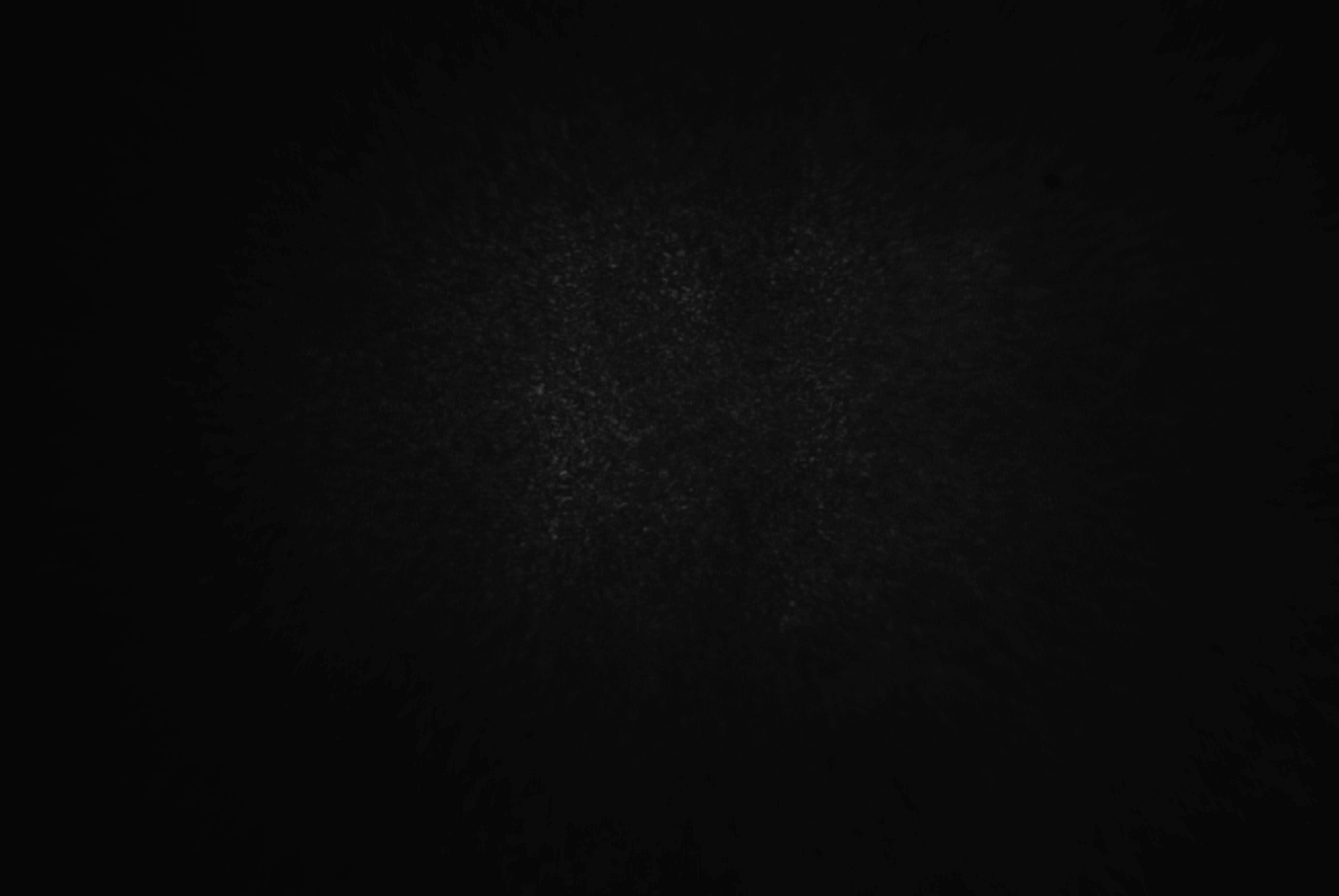

Supplement: Supplementary file 7 — Source Data [file 41467_2023_43674_MOESM7_ESM.zip › Source Data/Data 3/xx (17).JPG]

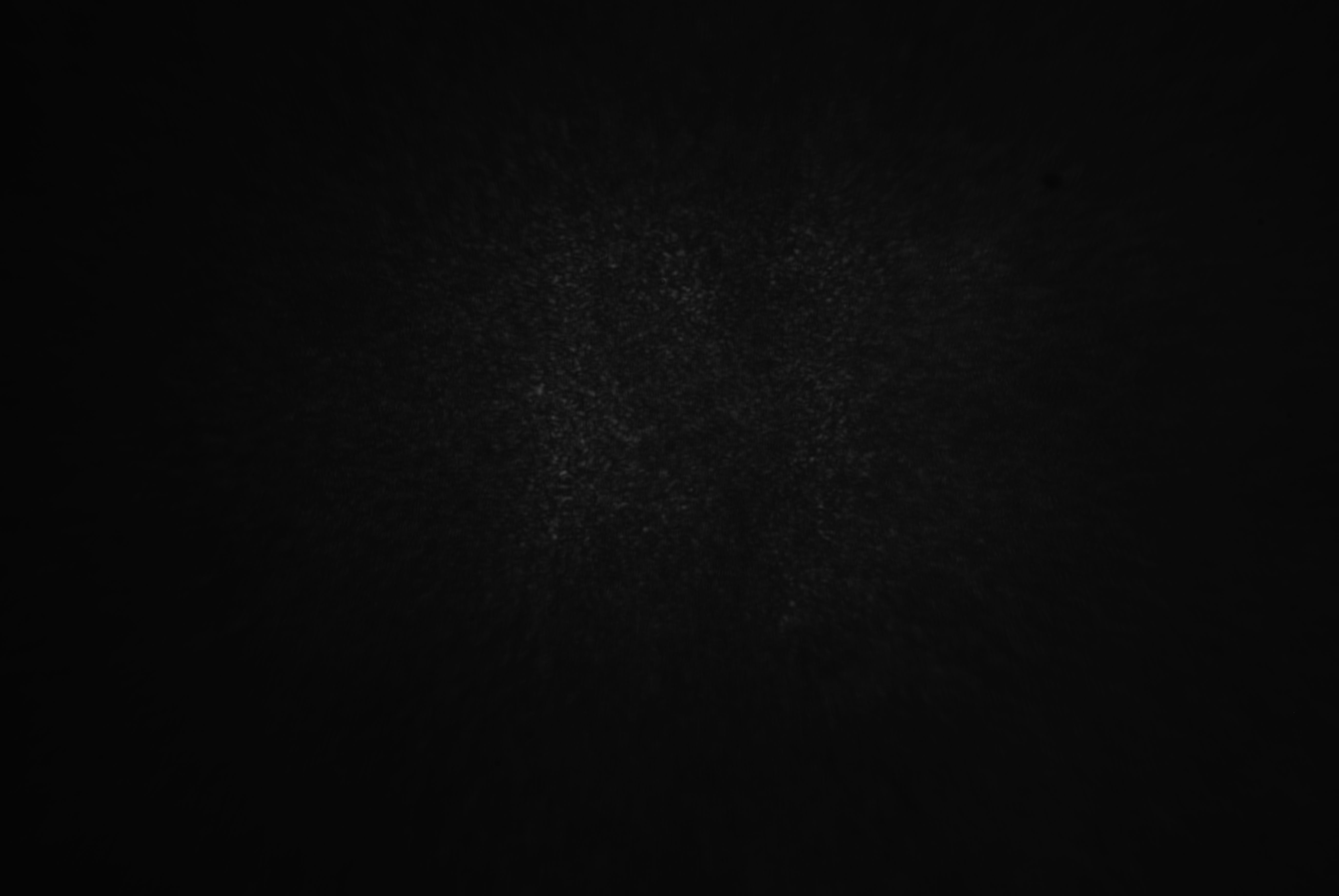

Supplement: Supplementary file 7 — Source Data [file 41467_2023_43674_MOESM7_ESM.zip › Source Data/Data 3/xx (18).JPG]

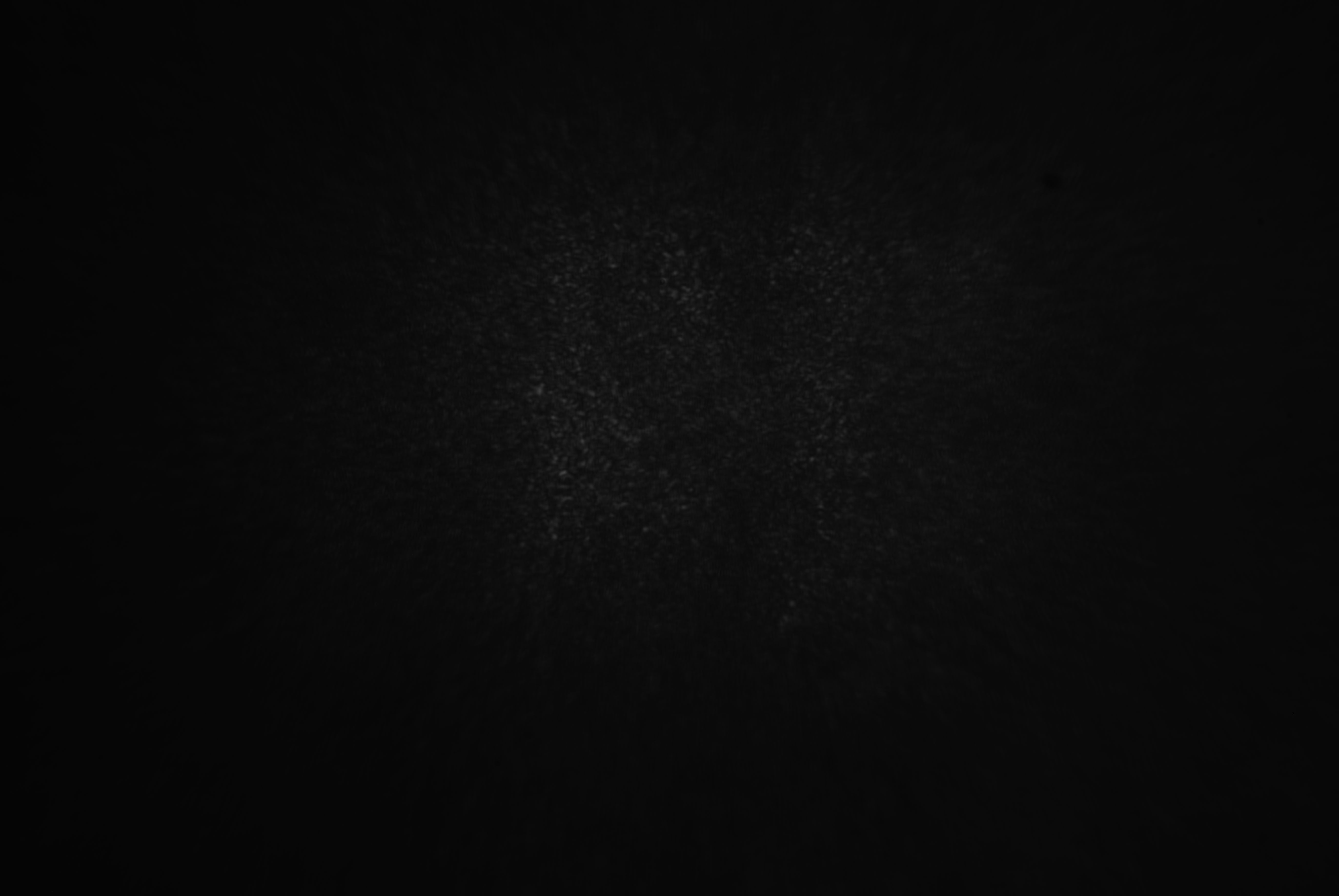

Supplement: Supplementary file 7 — Source Data [file 41467_2023_43674_MOESM7_ESM.zip › Source Data/Data 3/xx (19).JPG]

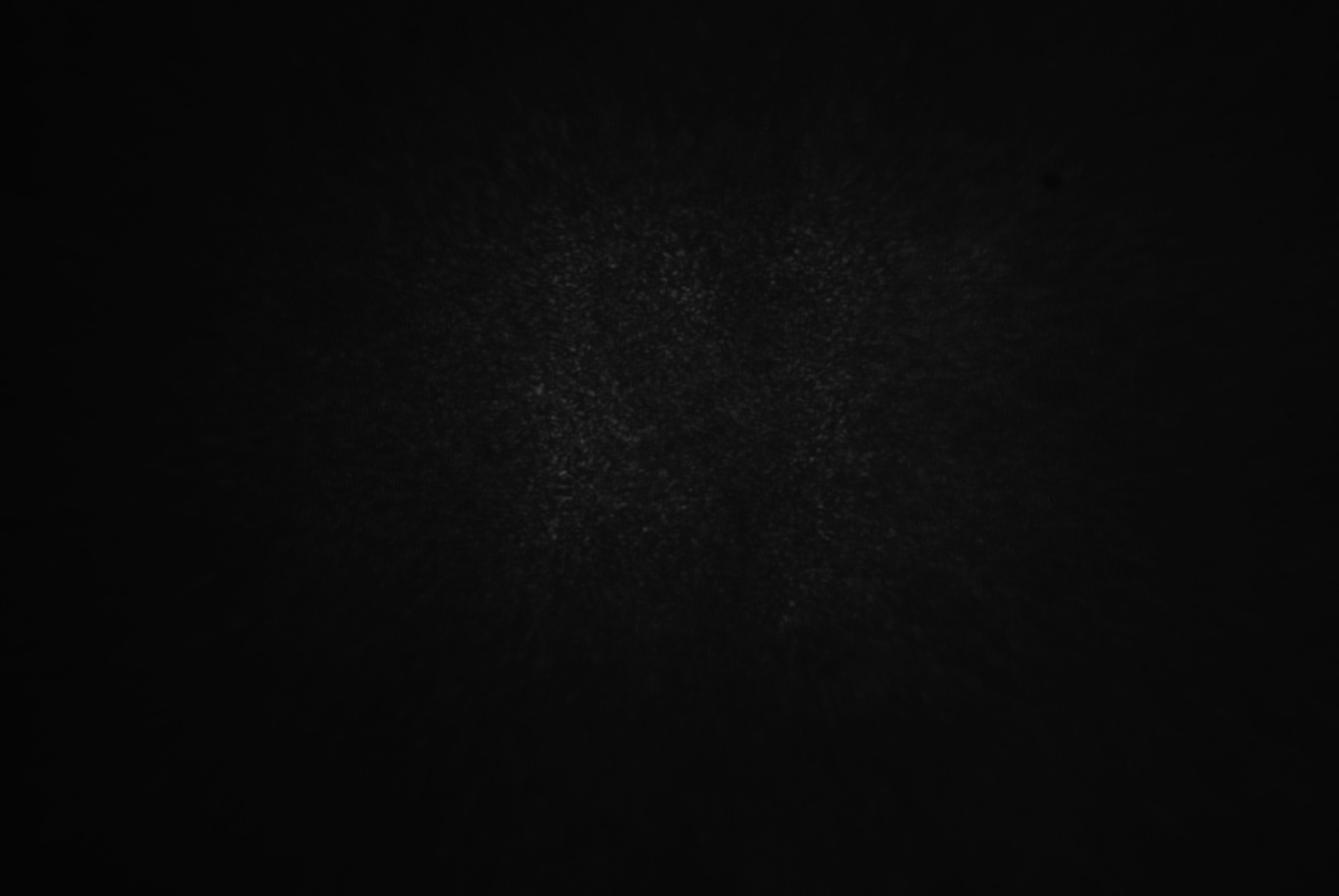

Supplement: Supplementary file 7 — Source Data [file 41467_2023_43674_MOESM7_ESM.zip › Source Data/Data 3/xx (20).JPG]

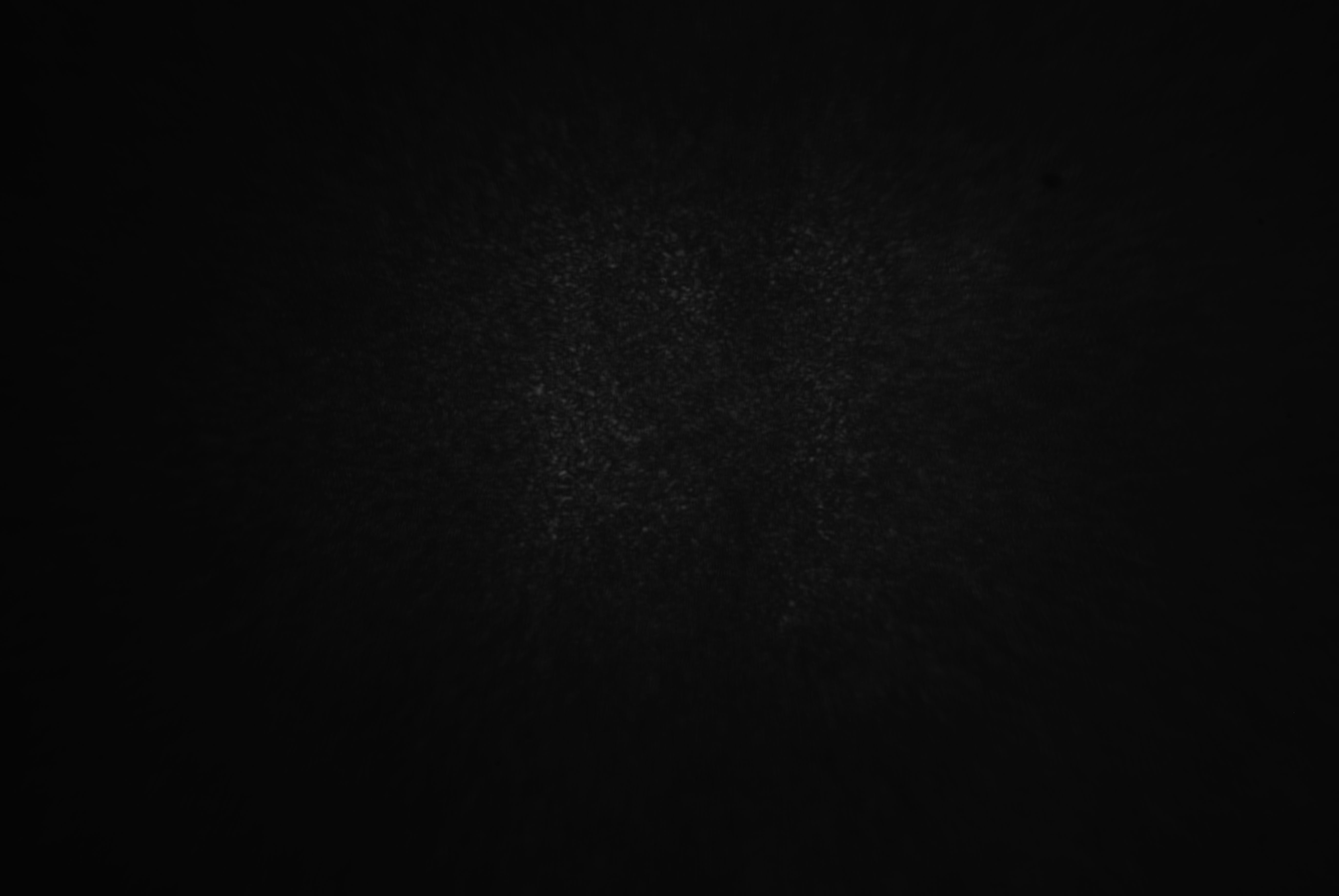

Supplement: Supplementary file 7 — Source Data [file 41467_2023_43674_MOESM7_ESM.zip › Source Data/Data 3/xx (21).JPG]

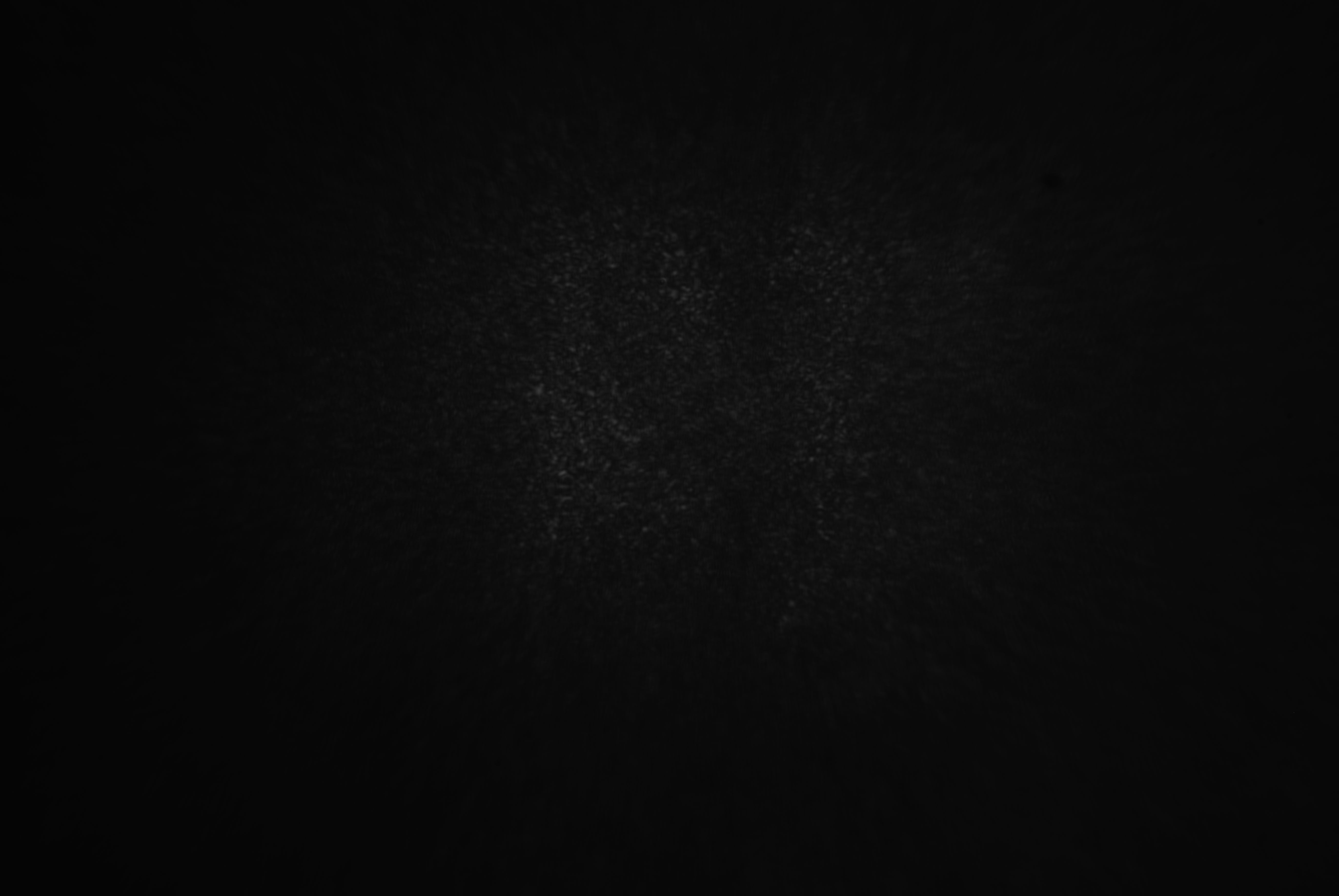

Supplement: Supplementary file 7 — Source Data [file 41467_2023_43674_MOESM7_ESM.zip › Source Data/Data 3/xx (22).JPG]

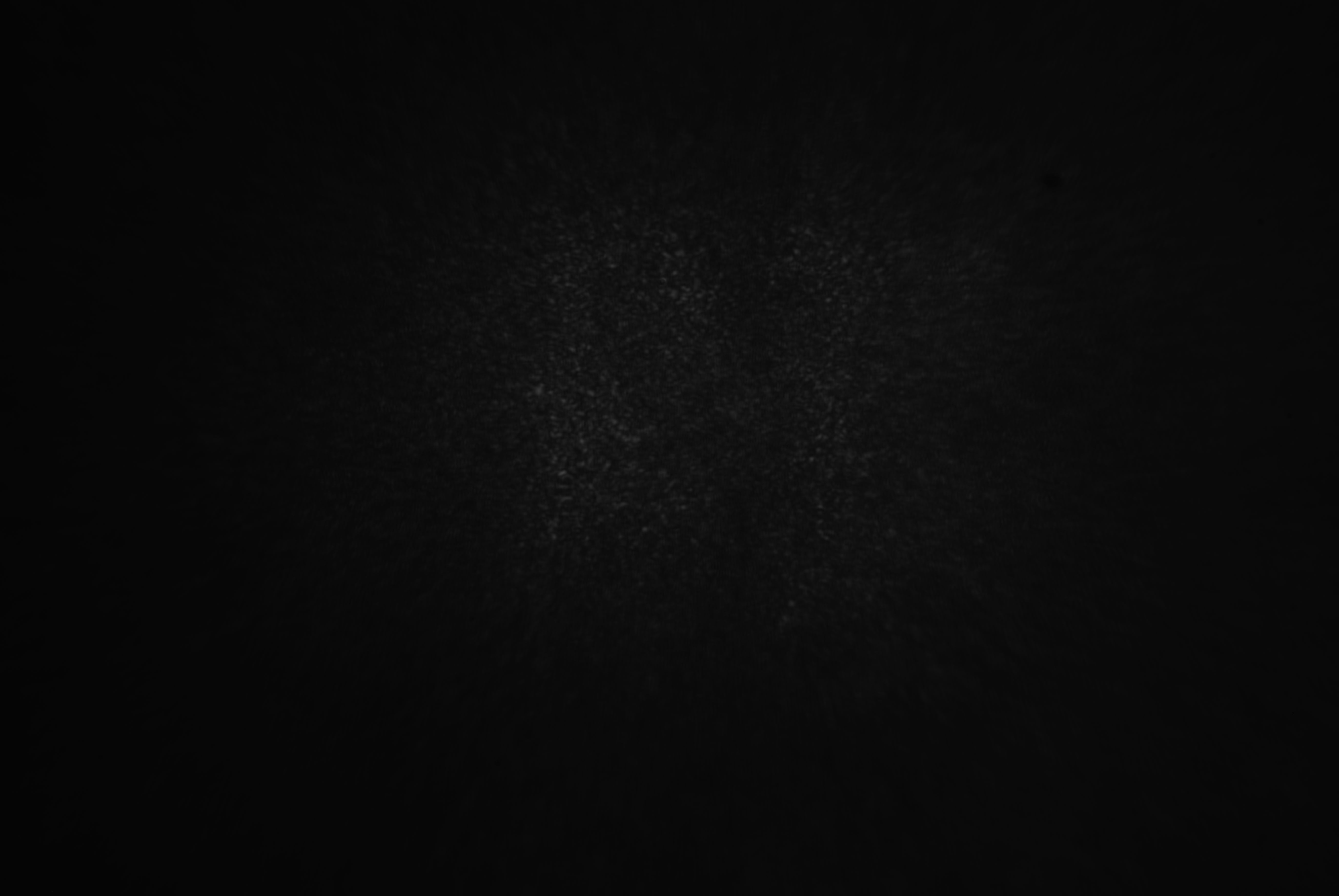

Supplement: Supplementary file 7 — Source Data [file 41467_2023_43674_MOESM7_ESM.zip › Source Data/Data 3/xx (23).JPG]

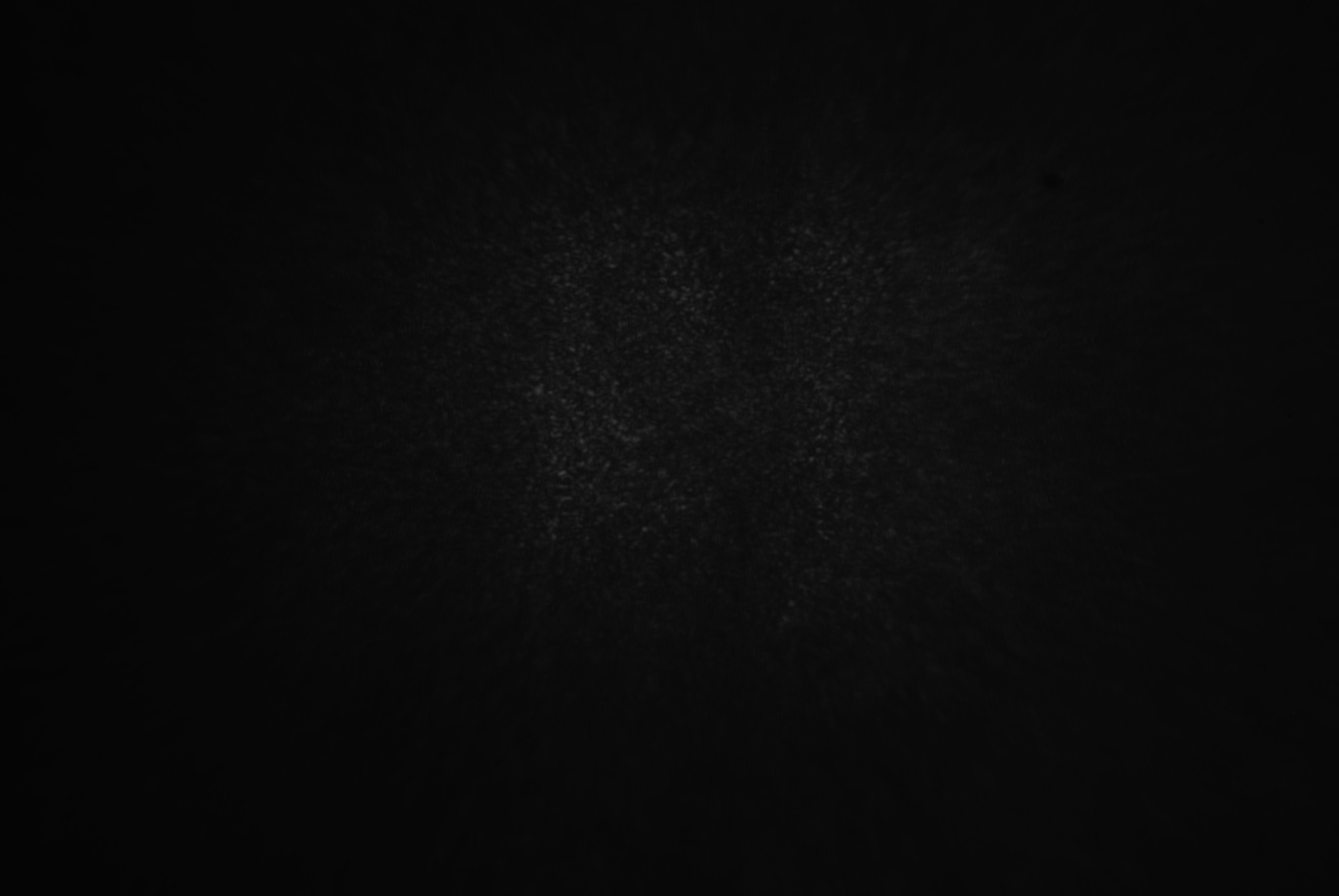

Supplement: Supplementary file 7 — Source Data [file 41467_2023_43674_MOESM7_ESM.zip › Source Data/Data 3/xx (24).JPG]

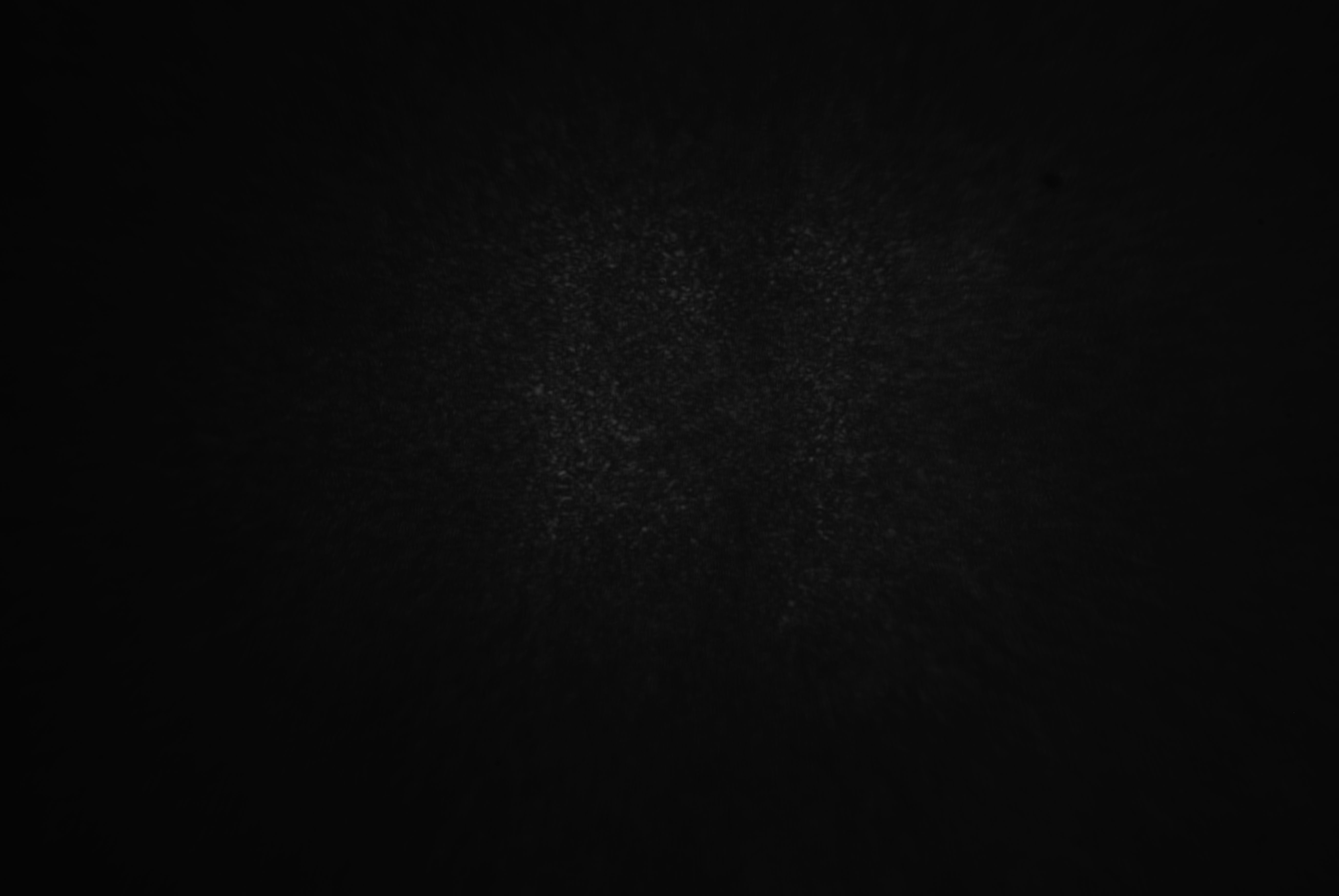

Supplement: Supplementary file 7 — Source Data [file 41467_2023_43674_MOESM7_ESM.zip › Source Data/Data 3/xx (25).JPG]

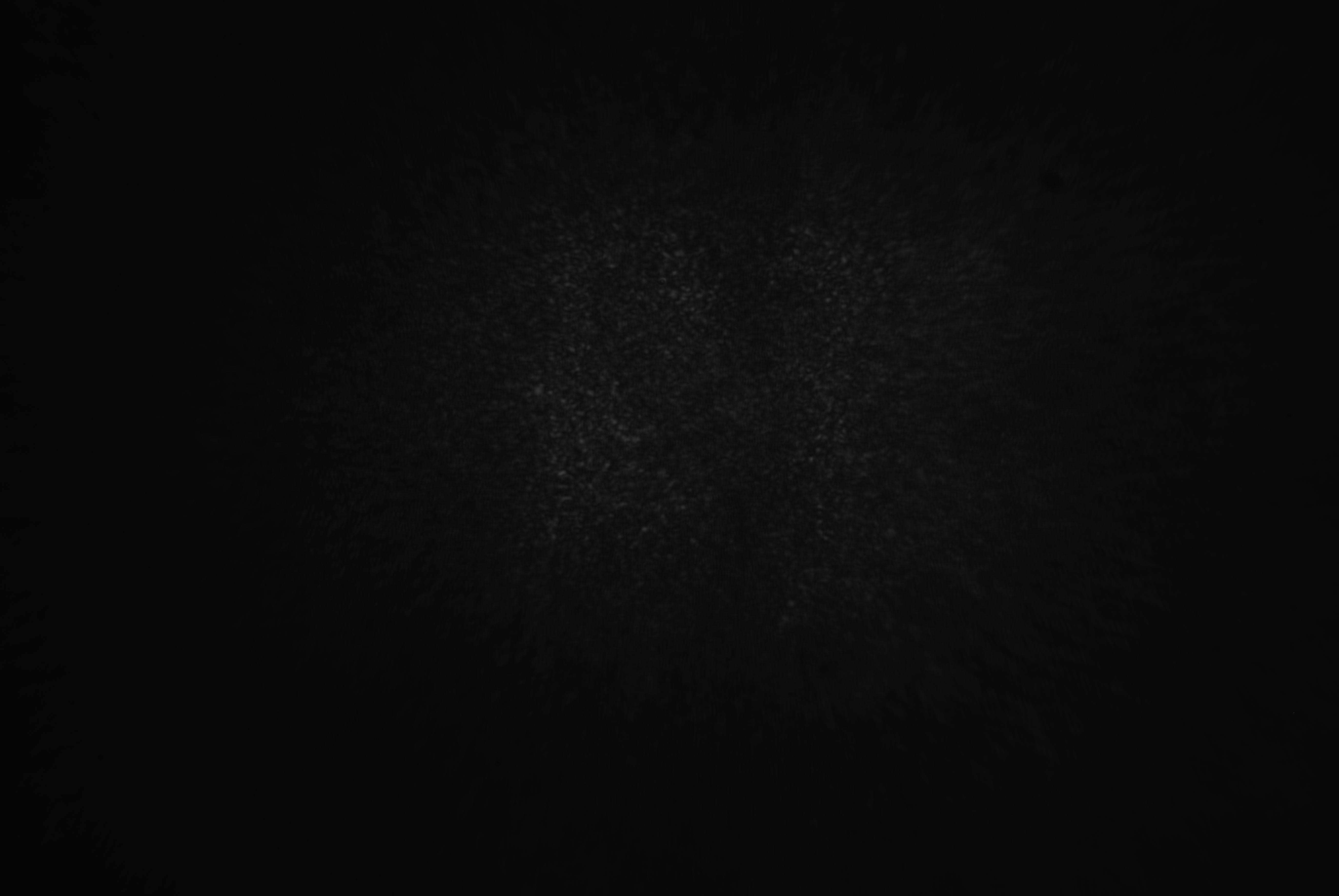

Supplement: Supplementary file 7 — Source Data [file 41467_2023_43674_MOESM7_ESM.zip › Source Data/Data 3/xx (26).JPG]

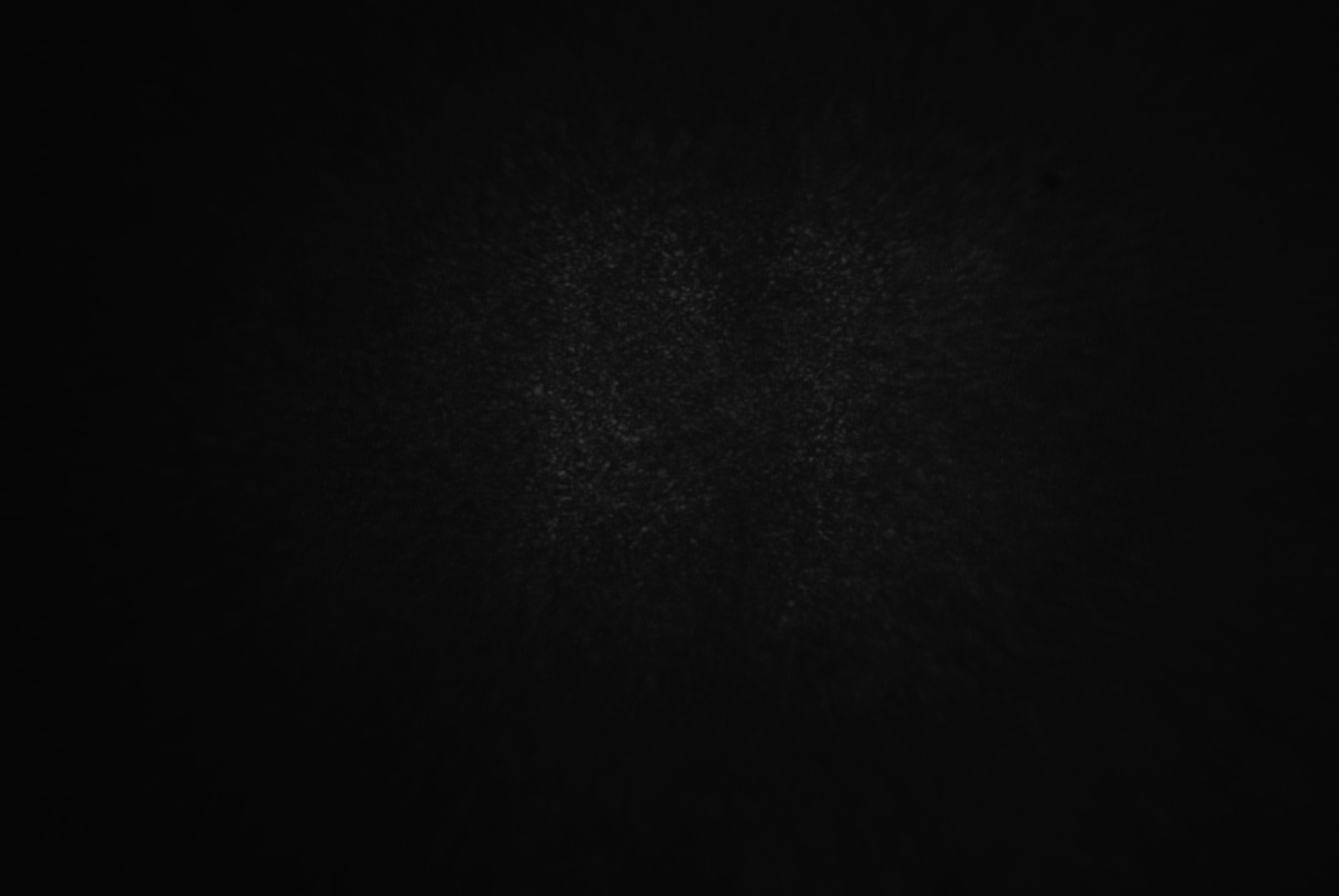

Supplement: Supplementary file 7 — Source Data [file 41467_2023_43674_MOESM7_ESM.zip › Source Data/Data 3/xx (27).JPG]

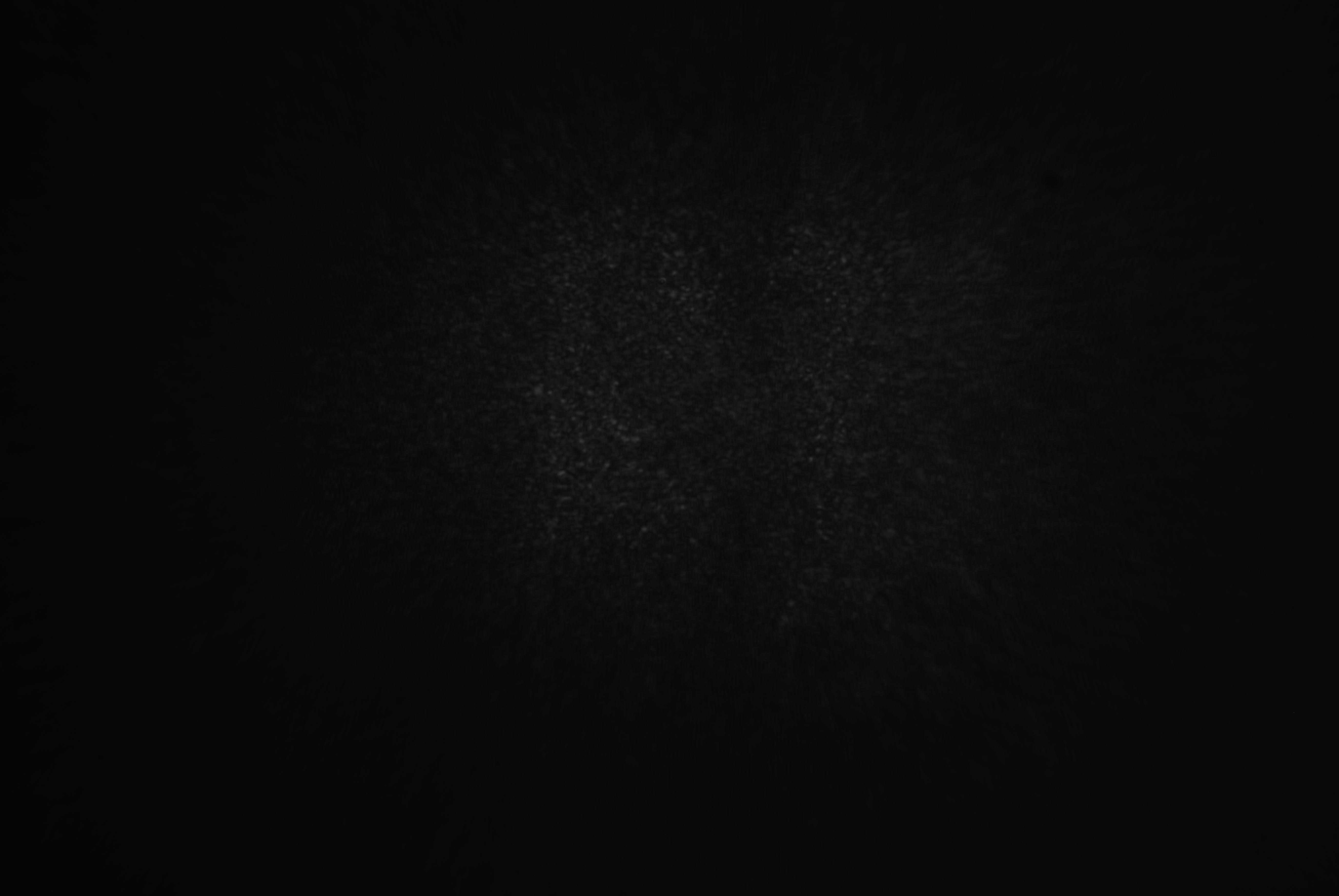

Supplement: Supplementary file 7 — Source Data [file 41467_2023_43674_MOESM7_ESM.zip › Source Data/Data 3/xx (28).JPG]

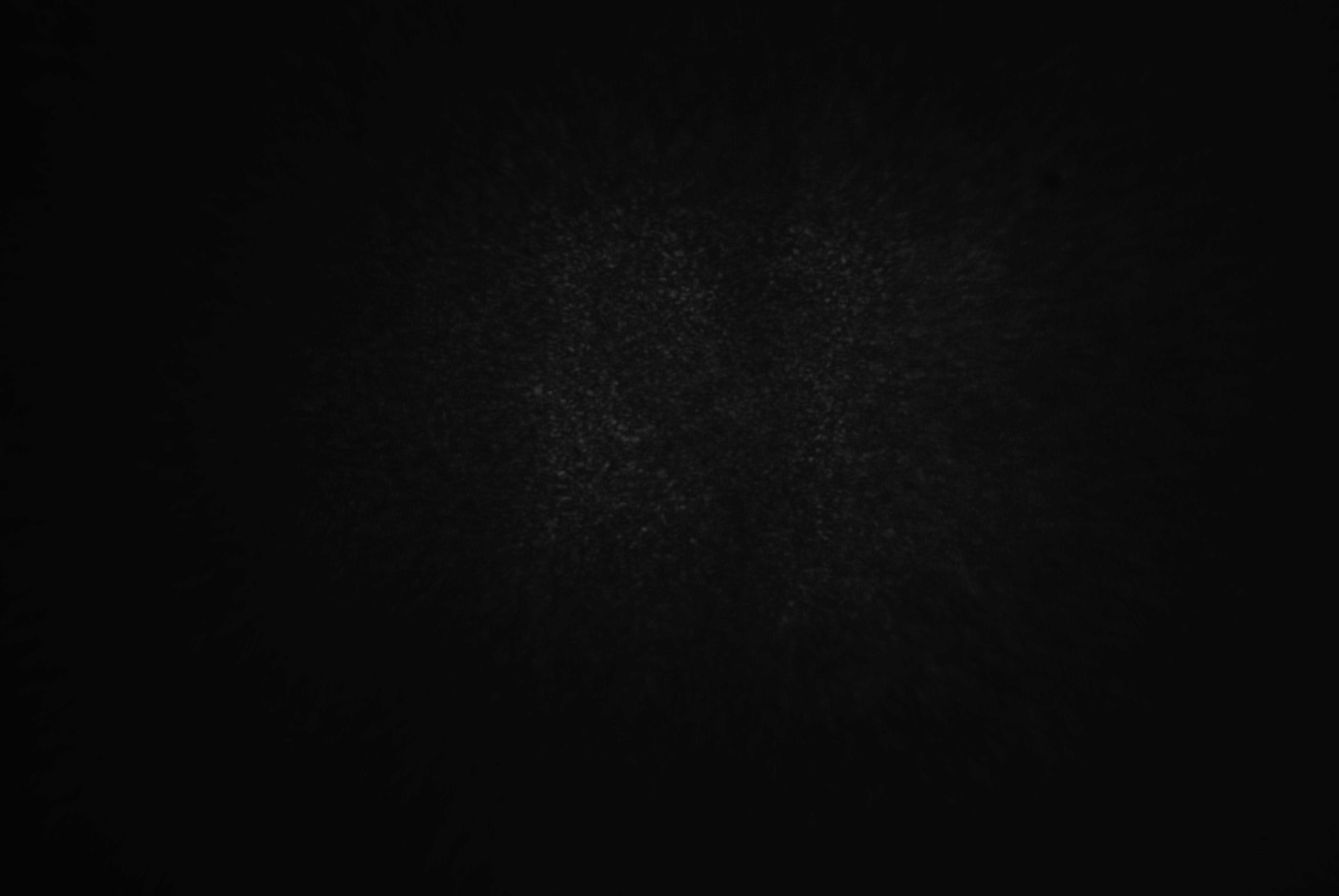

Supplement: Supplementary file 7 — Source Data [file 41467_2023_43674_MOESM7_ESM.zip › Source Data/Data 3/xx (29).JPG]

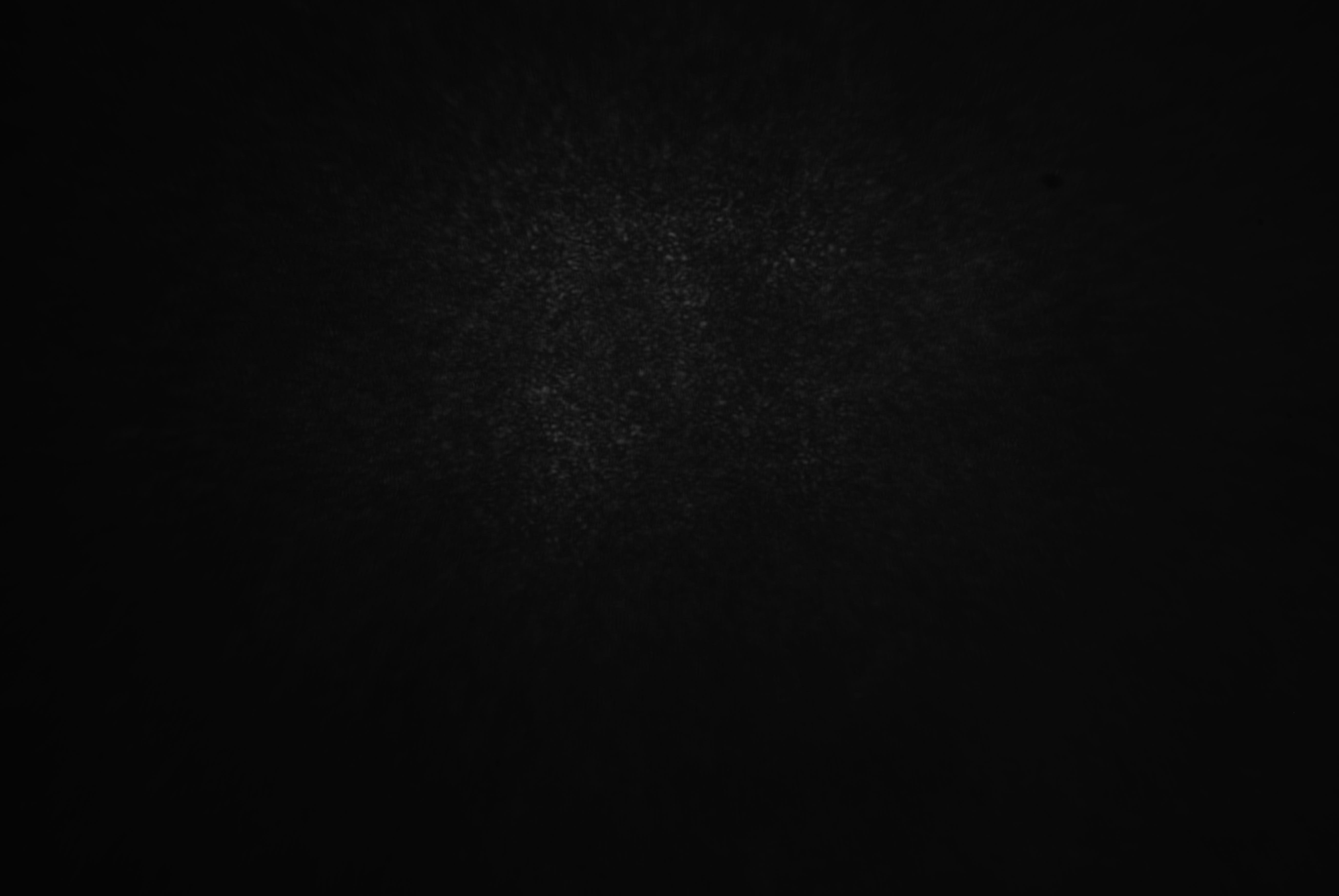

Supplement: Supplementary file 7 — Source Data [file 41467_2023_43674_MOESM7_ESM.zip › Source Data/Data 1/x (30).JPG]

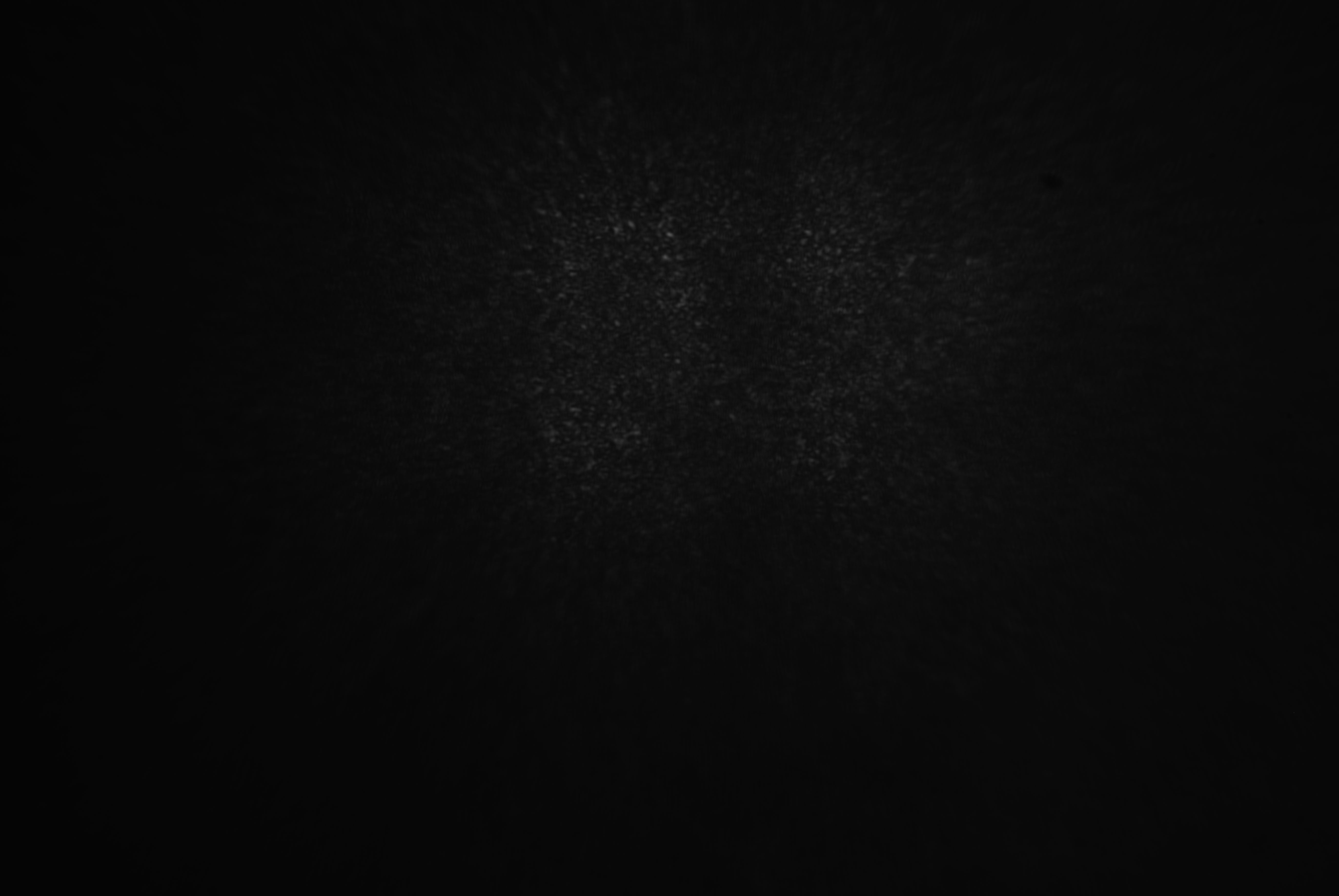

Supplement: Supplementary file 7 — Source Data [file 41467_2023_43674_MOESM7_ESM.zip › Source Data/Data 1/x (1).JPG]

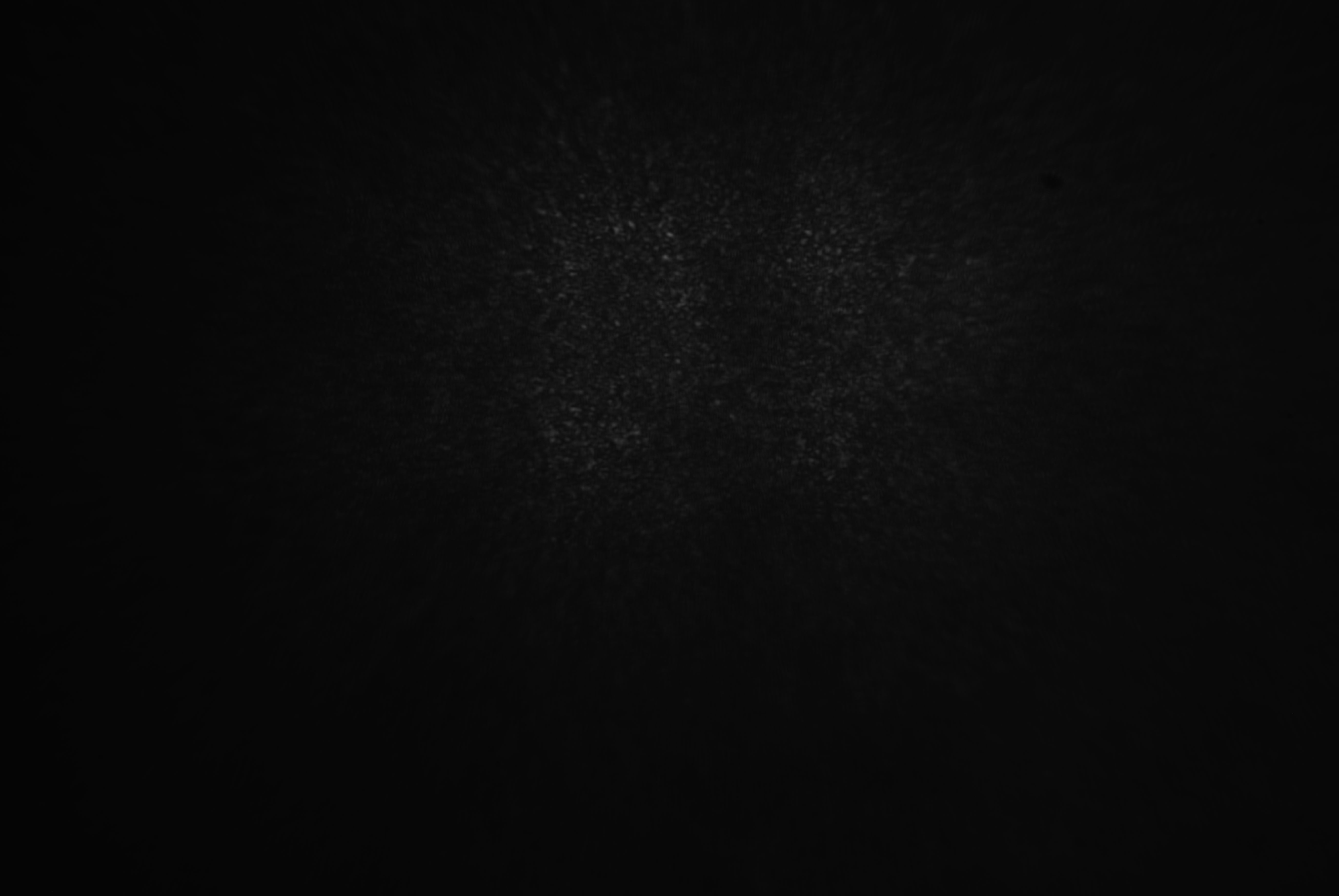

Supplement: Supplementary file 7 — Source Data [file 41467_2023_43674_MOESM7_ESM.zip › Source Data/Data 1/x (2).JPG]

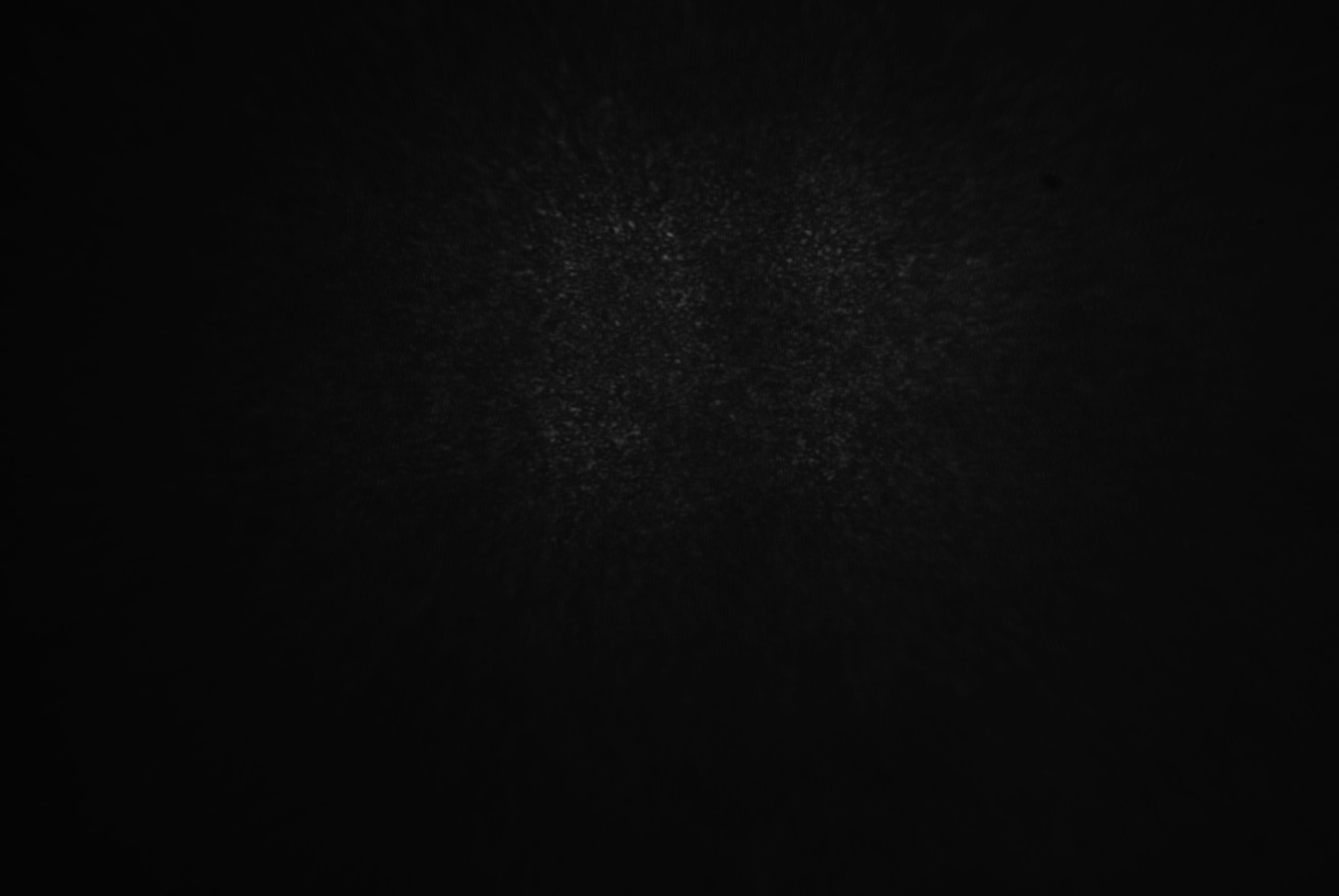

Supplement: Supplementary file 7 — Source Data [file 41467_2023_43674_MOESM7_ESM.zip › Source Data/Data 1/x (3).JPG]

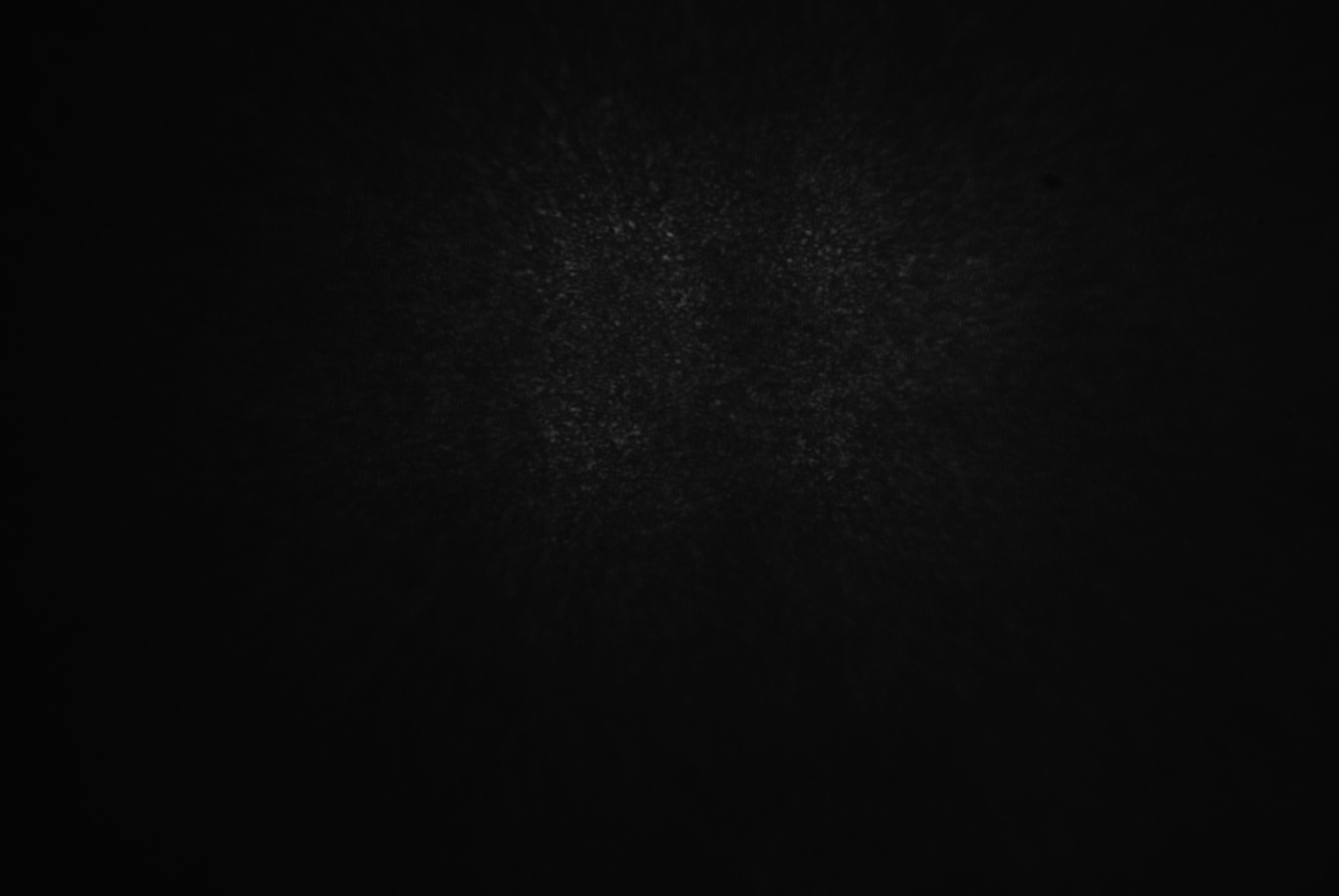

Supplement: Supplementary file 7 — Source Data [file 41467_2023_43674_MOESM7_ESM.zip › Source Data/Data 1/x (4).JPG]

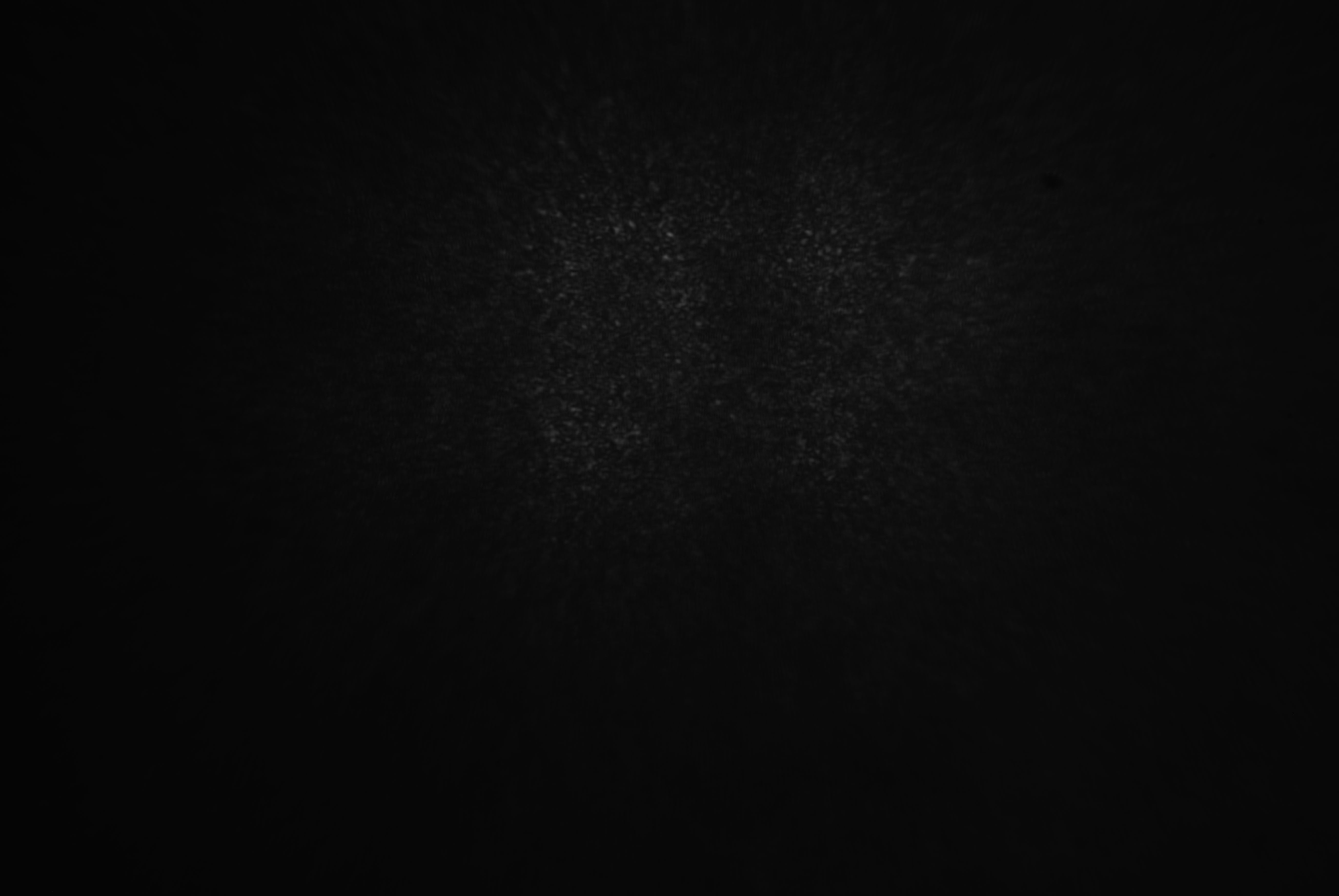

Supplement: Supplementary file 7 — Source Data [file 41467_2023_43674_MOESM7_ESM.zip › Source Data/Data 1/x (5).JPG]

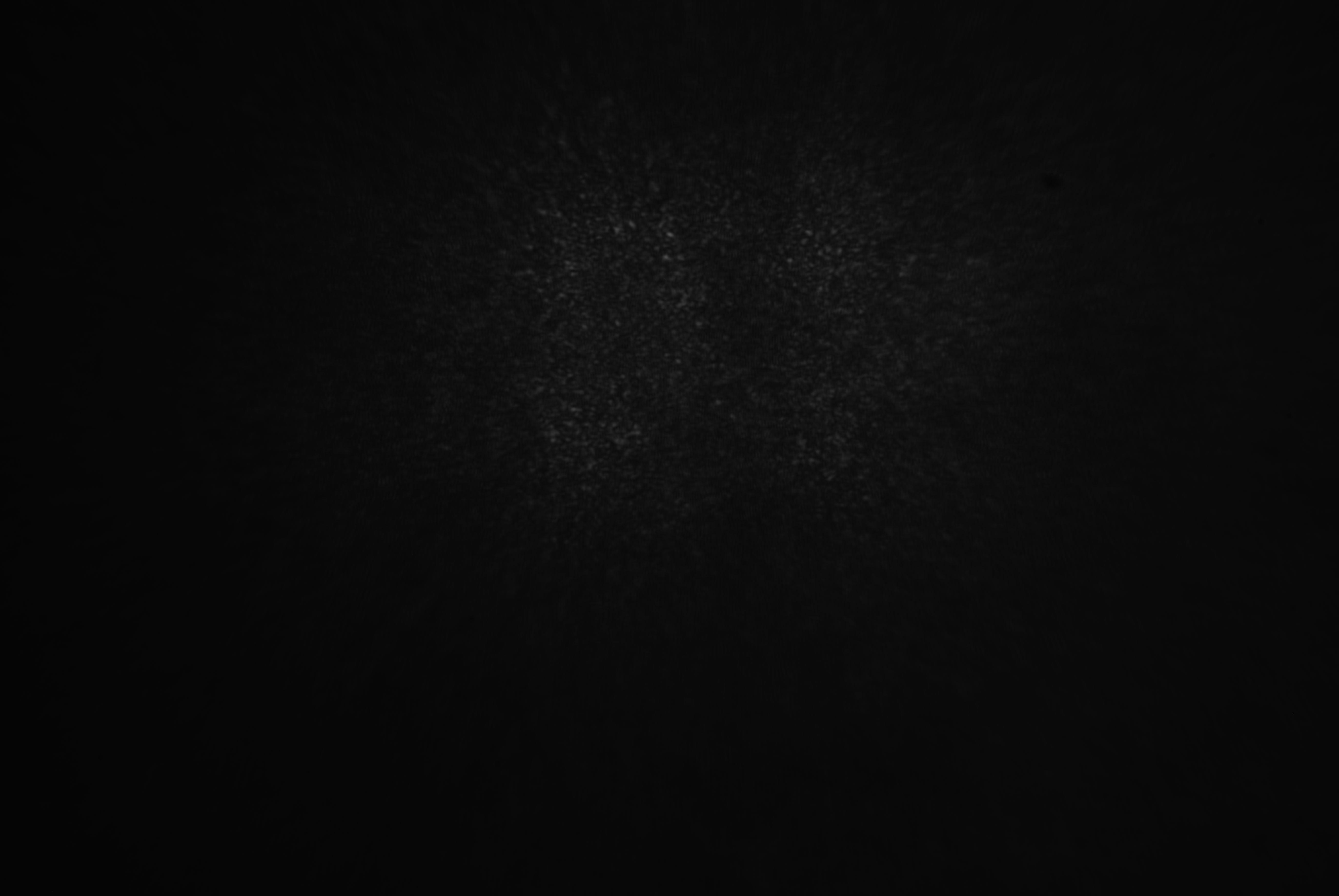

Supplement: Supplementary file 7 — Source Data [file 41467_2023_43674_MOESM7_ESM.zip › Source Data/Data 1/x (6).JPG]

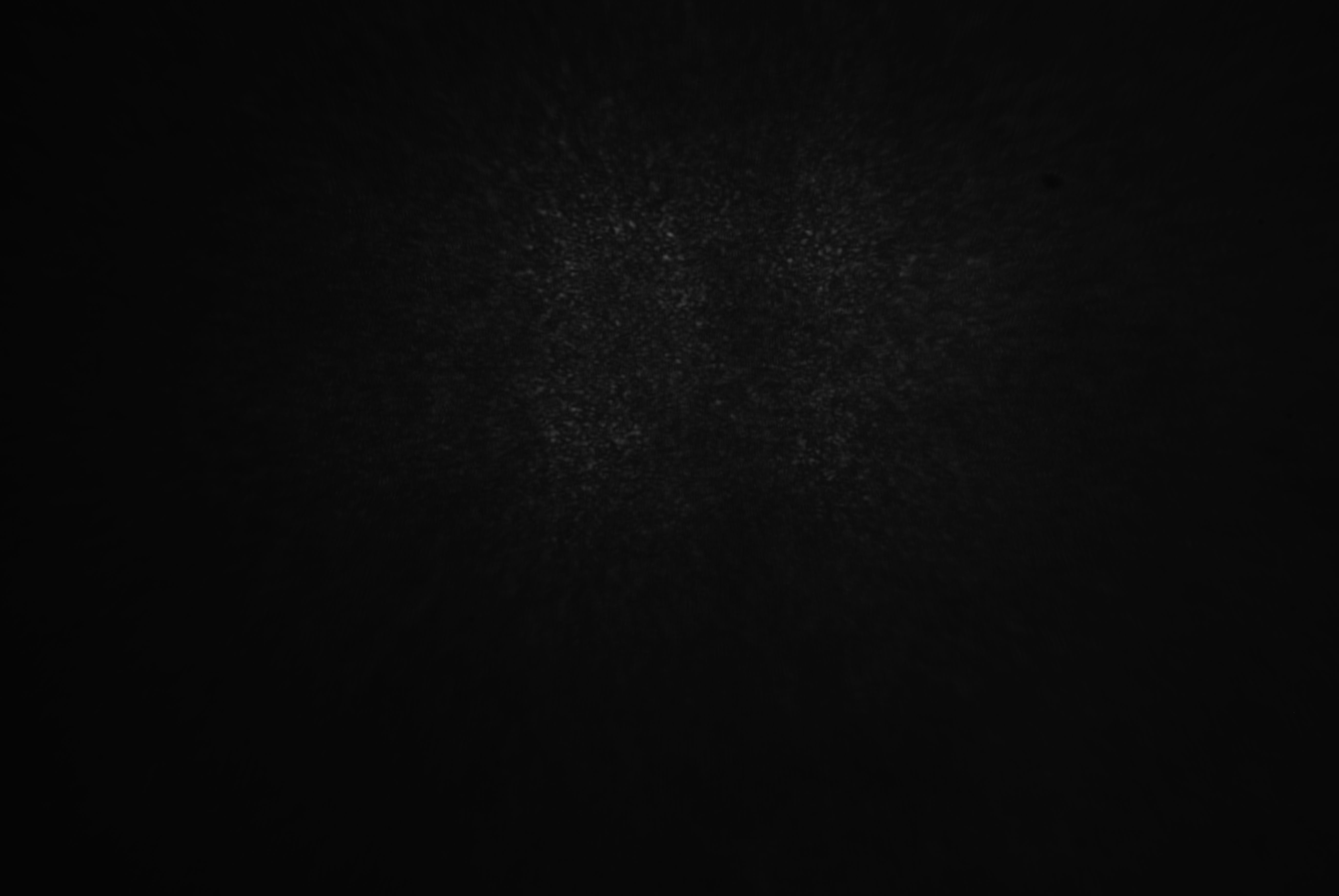

Supplement: Supplementary file 7 — Source Data [file 41467_2023_43674_MOESM7_ESM.zip › Source Data/Data 1/x (7).JPG]

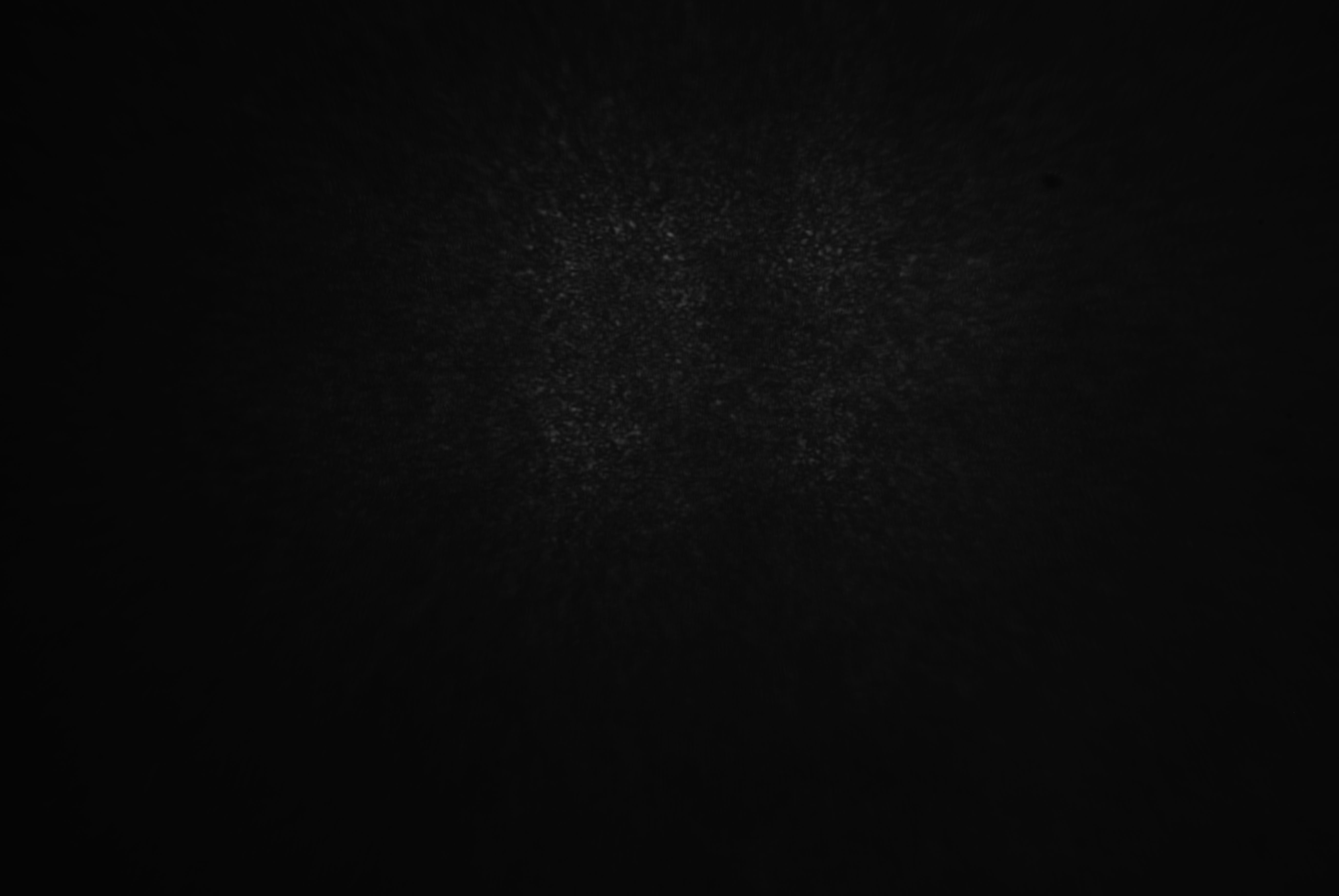

Supplement: Supplementary file 7 — Source Data [file 41467_2023_43674_MOESM7_ESM.zip › Source Data/Data 1/x (8).JPG]

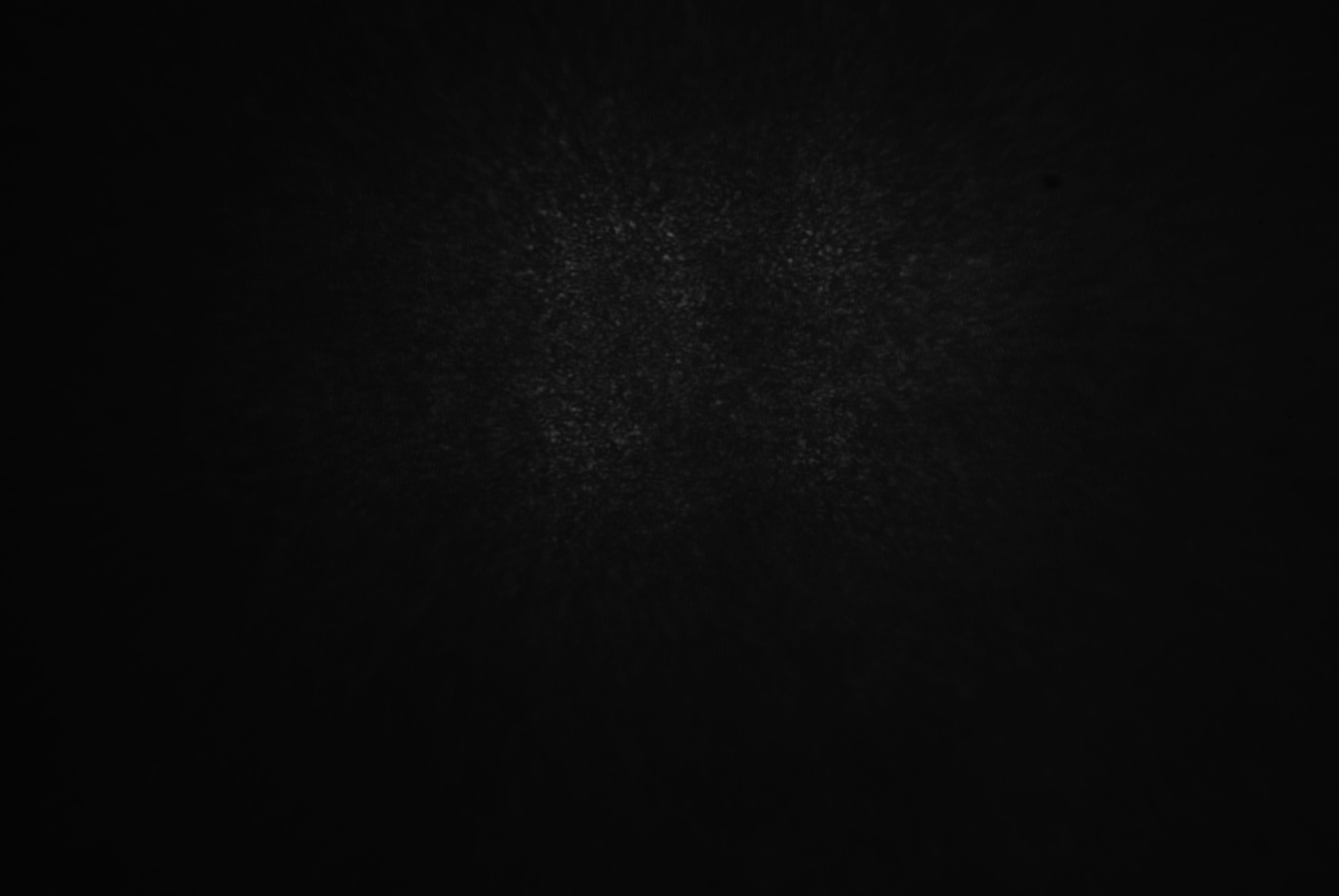

Supplement: Supplementary file 7 — Source Data [file 41467_2023_43674_MOESM7_ESM.zip › Source Data/Data 1/x (9).JPG]

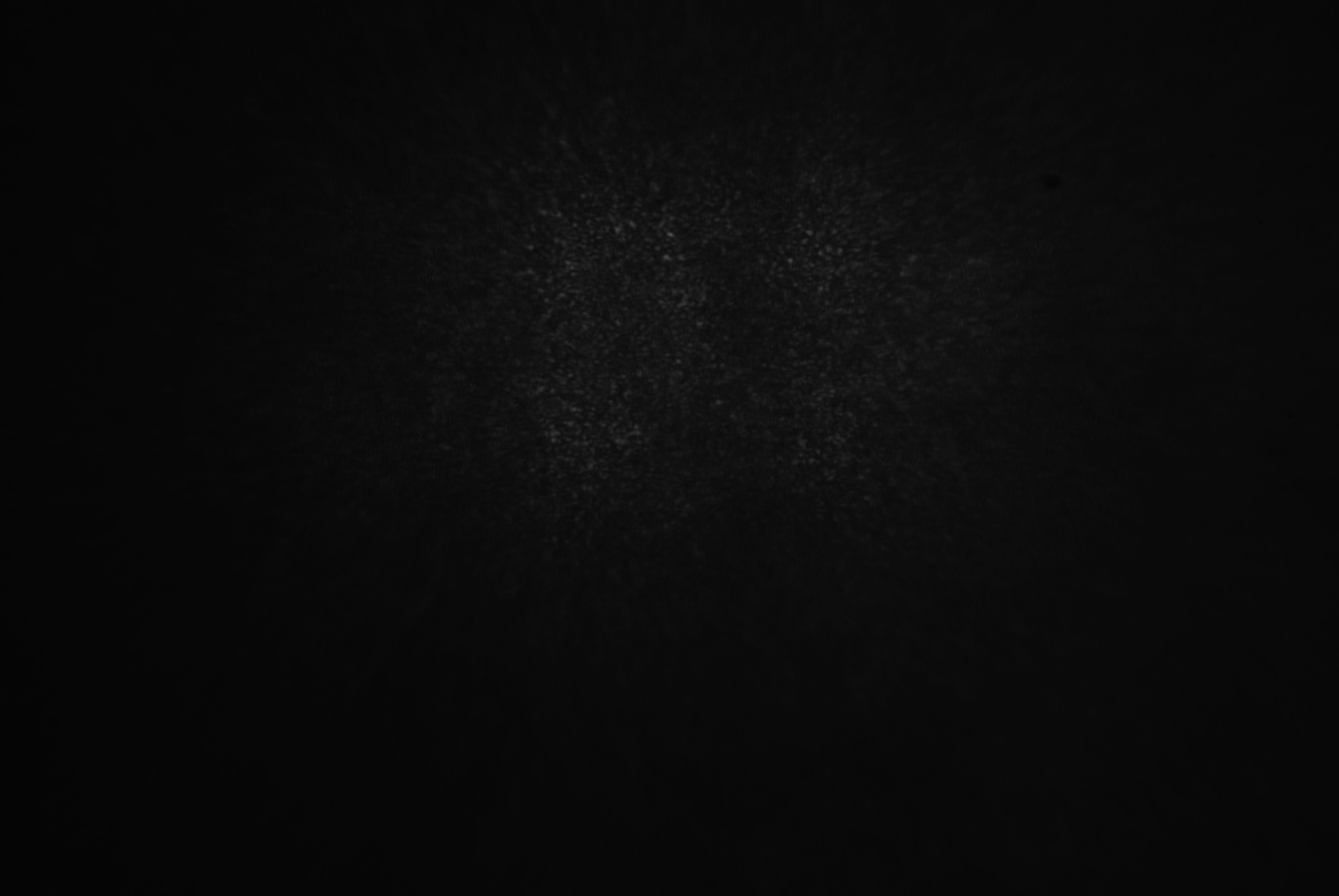

Supplement: Supplementary file 7 — Source Data [file 41467_2023_43674_MOESM7_ESM.zip › Source Data/Data 1/x (10).JPG]

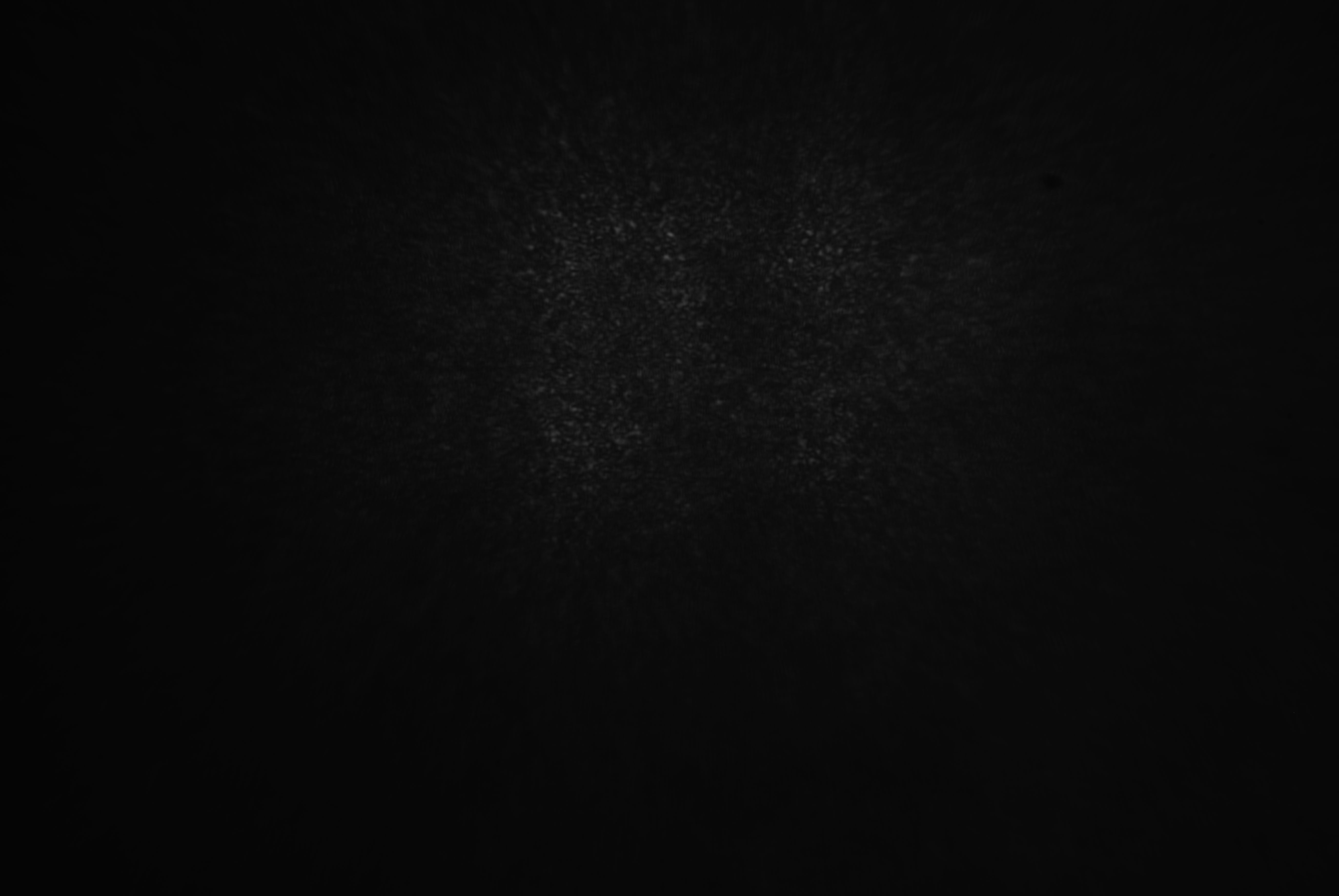

Supplement: Supplementary file 7 — Source Data [file 41467_2023_43674_MOESM7_ESM.zip › Source Data/Data 1/x (11).JPG]

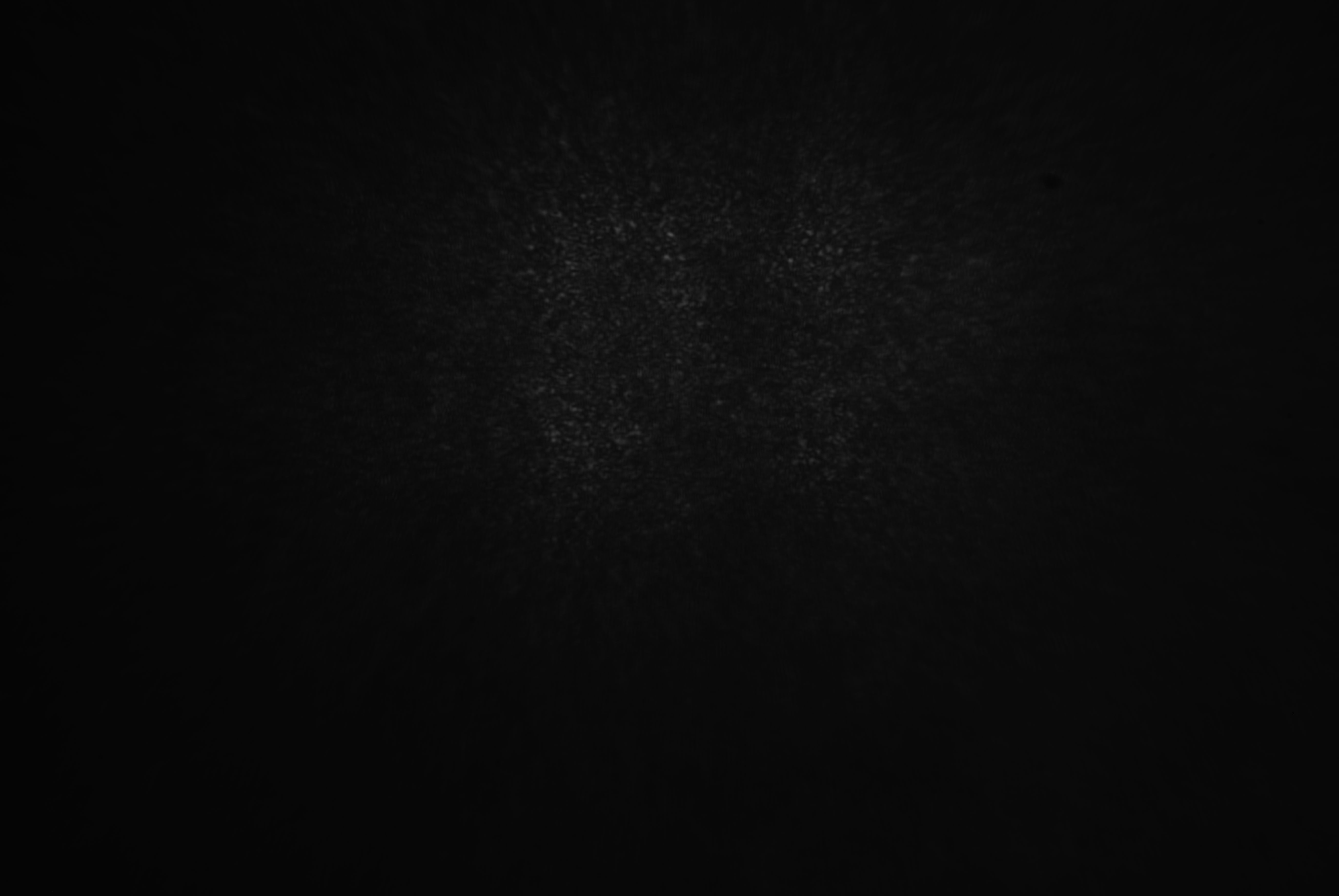

Supplement: Supplementary file 7 — Source Data [file 41467_2023_43674_MOESM7_ESM.zip › Source Data/Data 1/x (12).JPG]

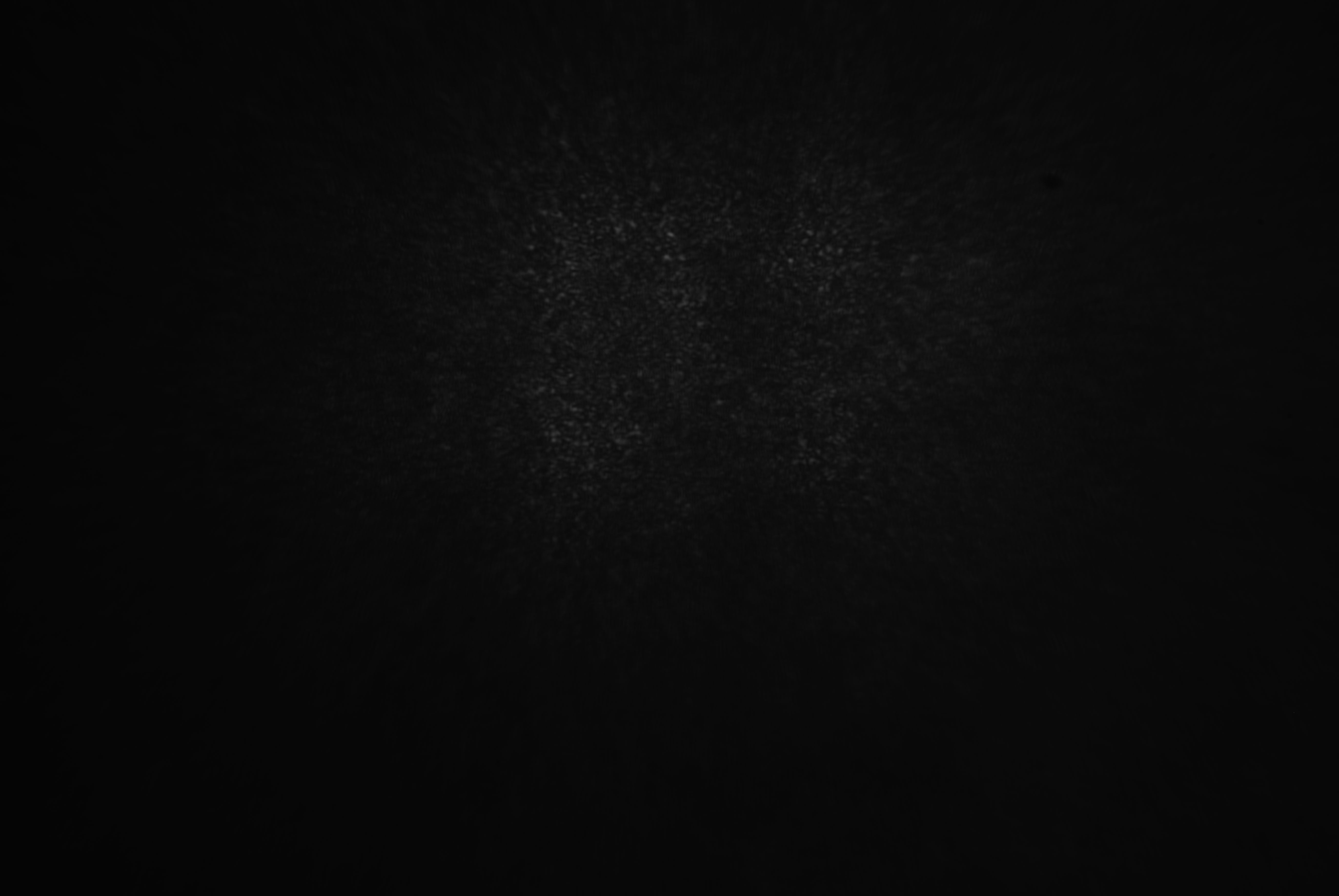

Supplement: Supplementary file 7 — Source Data [file 41467_2023_43674_MOESM7_ESM.zip › Source Data/Data 1/x (13).JPG]

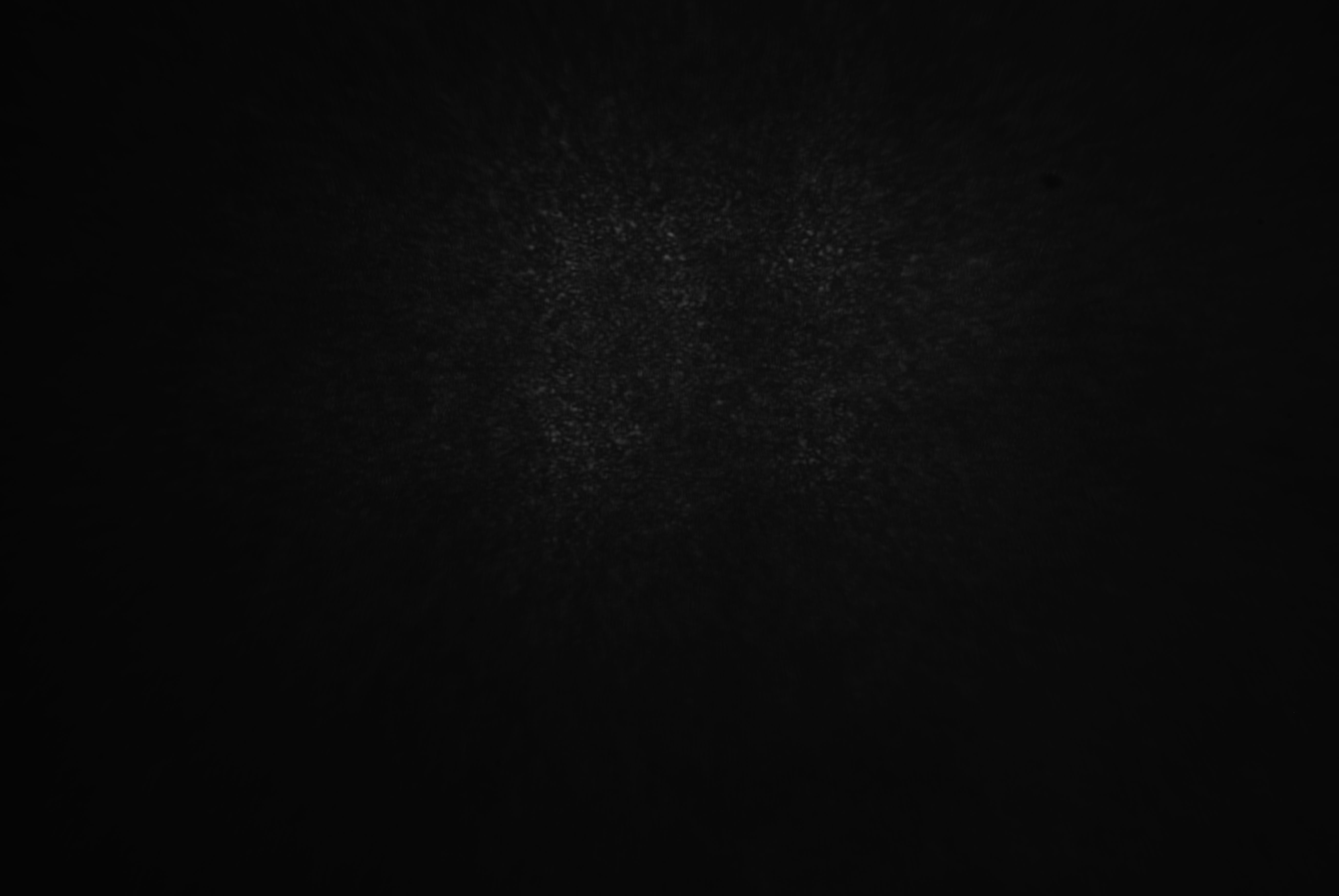

Supplement: Supplementary file 7 — Source Data [file 41467_2023_43674_MOESM7_ESM.zip › Source Data/Data 1/x (14).JPG]

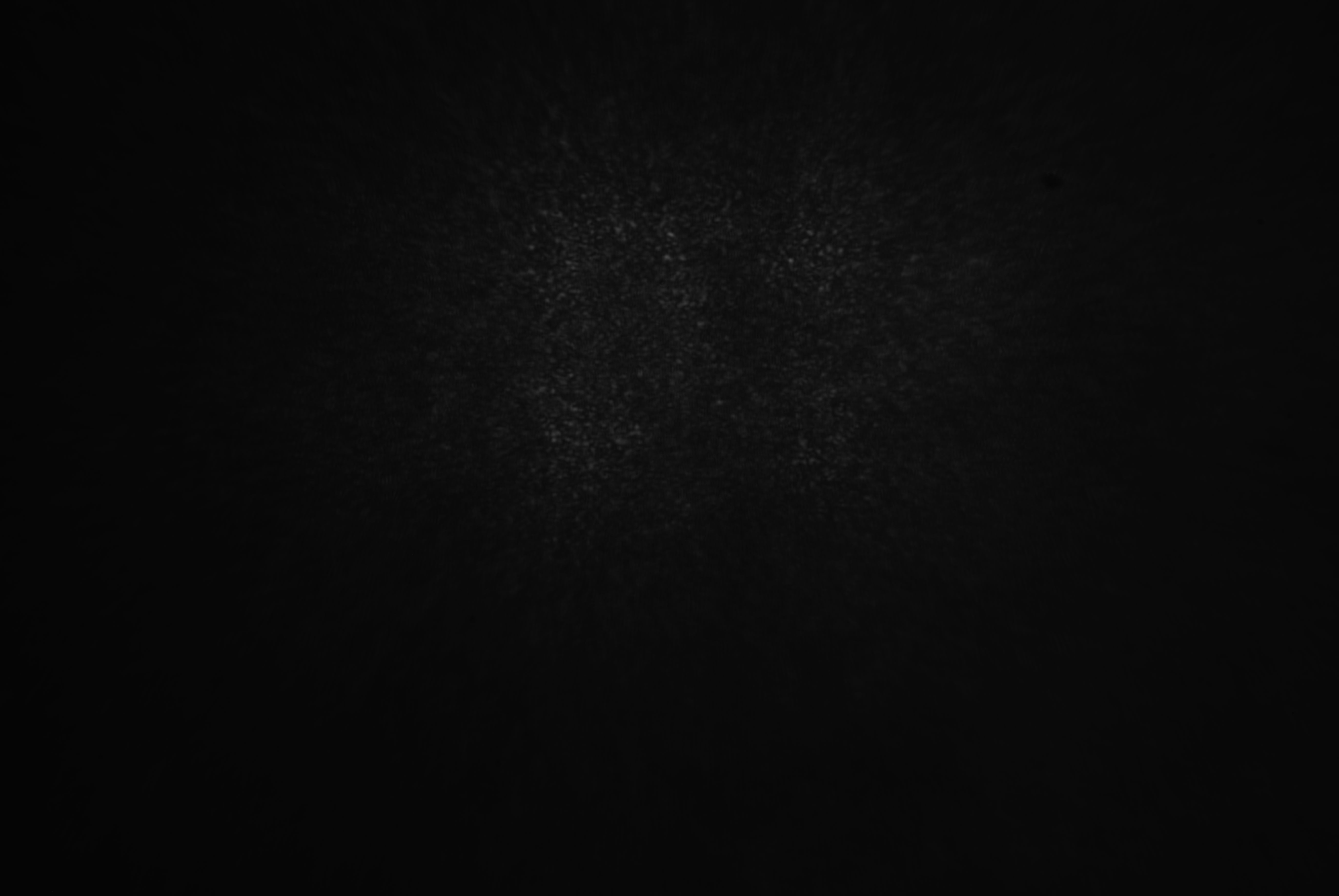

Supplement: Supplementary file 7 — Source Data [file 41467_2023_43674_MOESM7_ESM.zip › Source Data/Data 1/x (15).JPG]

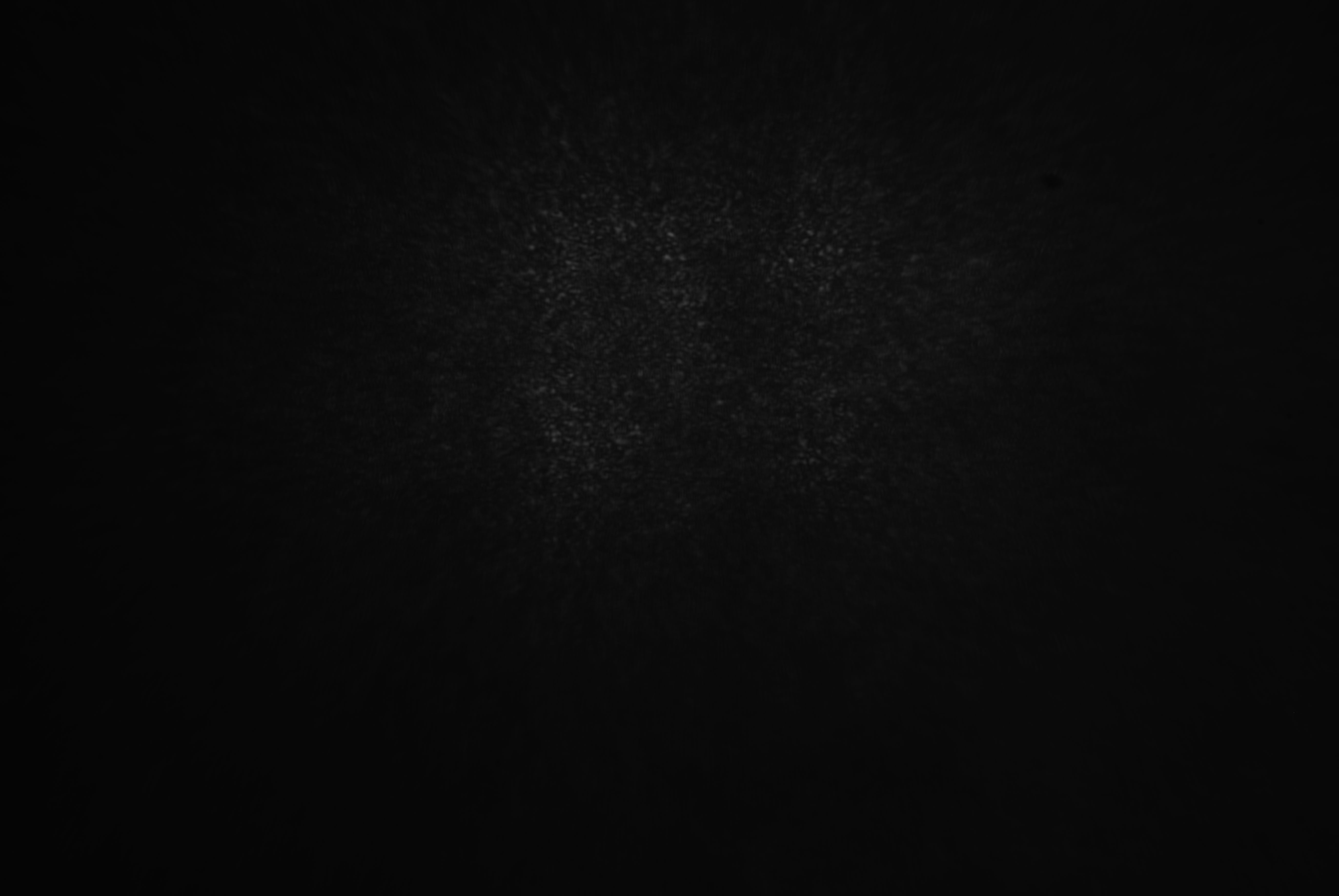

Supplement: Supplementary file 7 — Source Data [file 41467_2023_43674_MOESM7_ESM.zip › Source Data/Data 1/x (16).JPG]

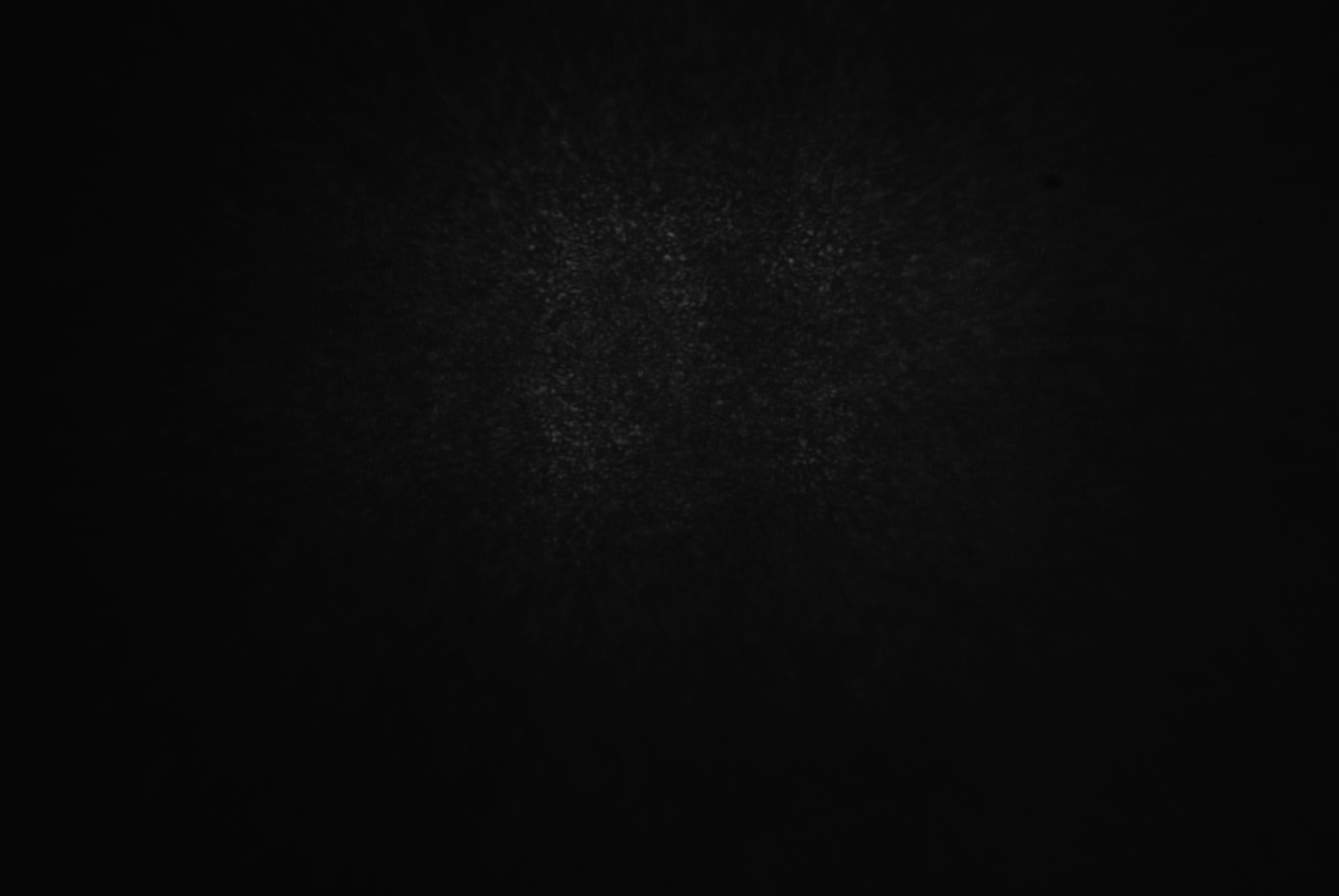

Supplement: Supplementary file 7 — Source Data [file 41467_2023_43674_MOESM7_ESM.zip › Source Data/Data 1/x (17).JPG]

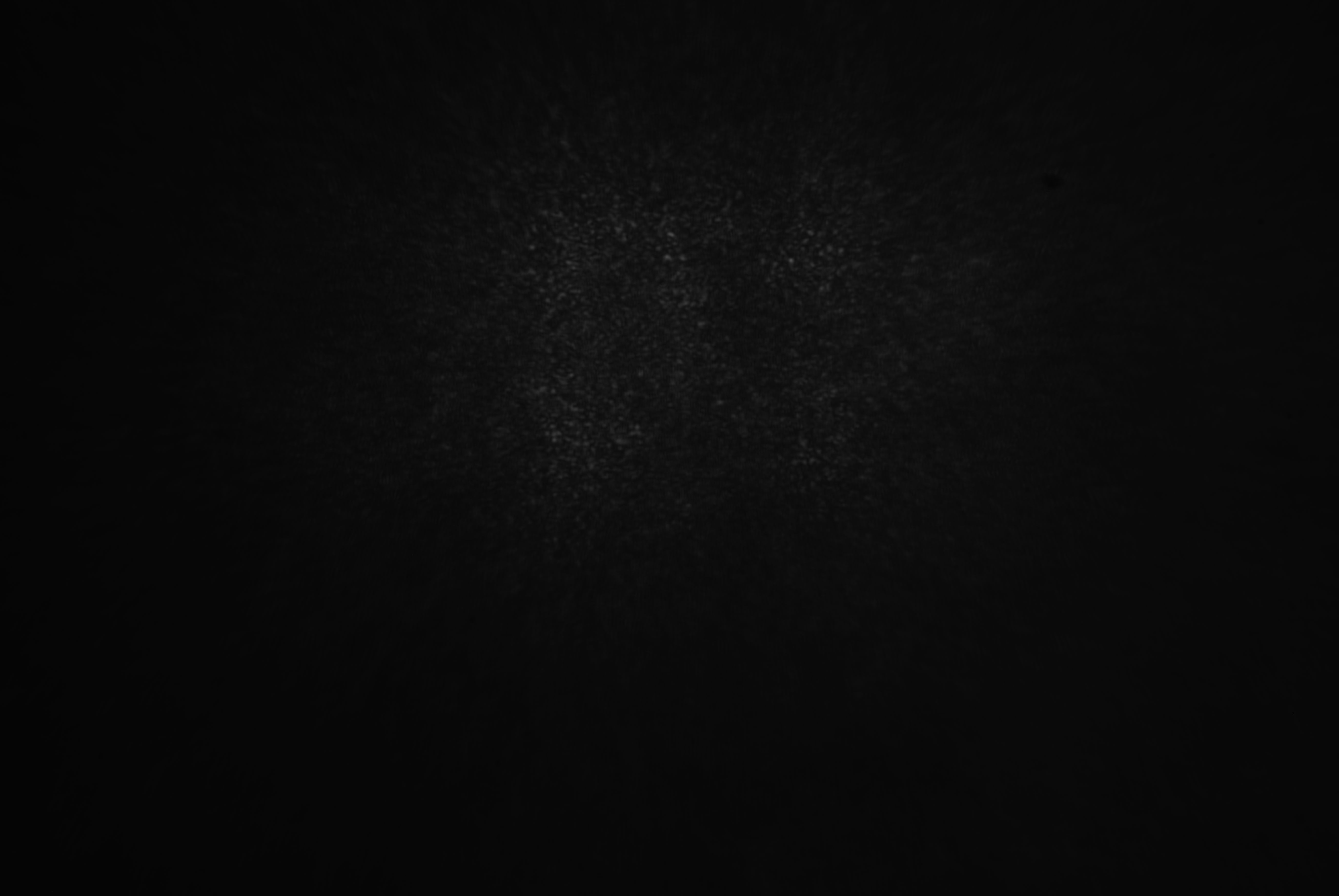

Supplement: Supplementary file 7 — Source Data [file 41467_2023_43674_MOESM7_ESM.zip › Source Data/Data 1/x (18).JPG]

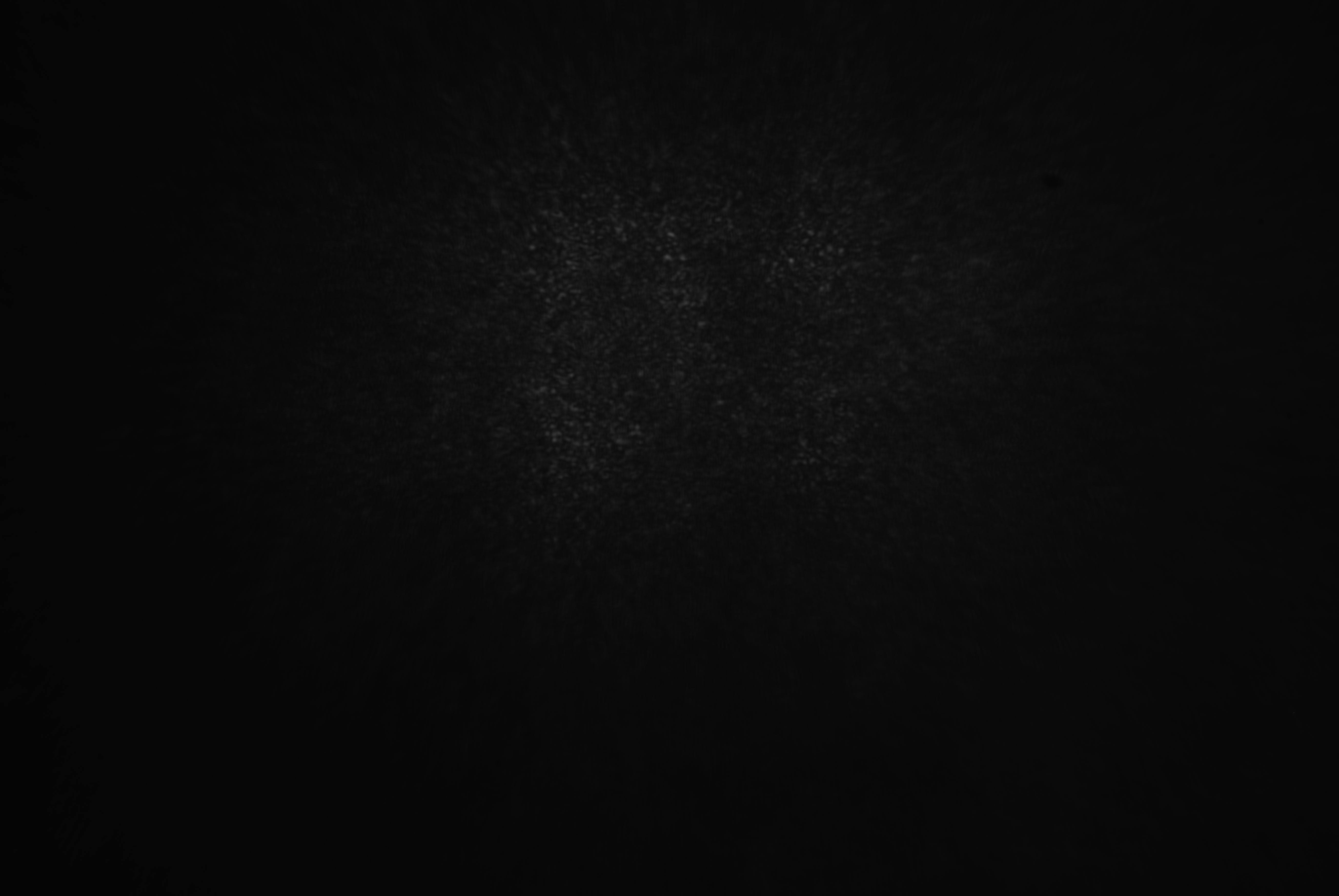

Supplement: Supplementary file 7 — Source Data [file 41467_2023_43674_MOESM7_ESM.zip › Source Data/Data 1/x (19).JPG]

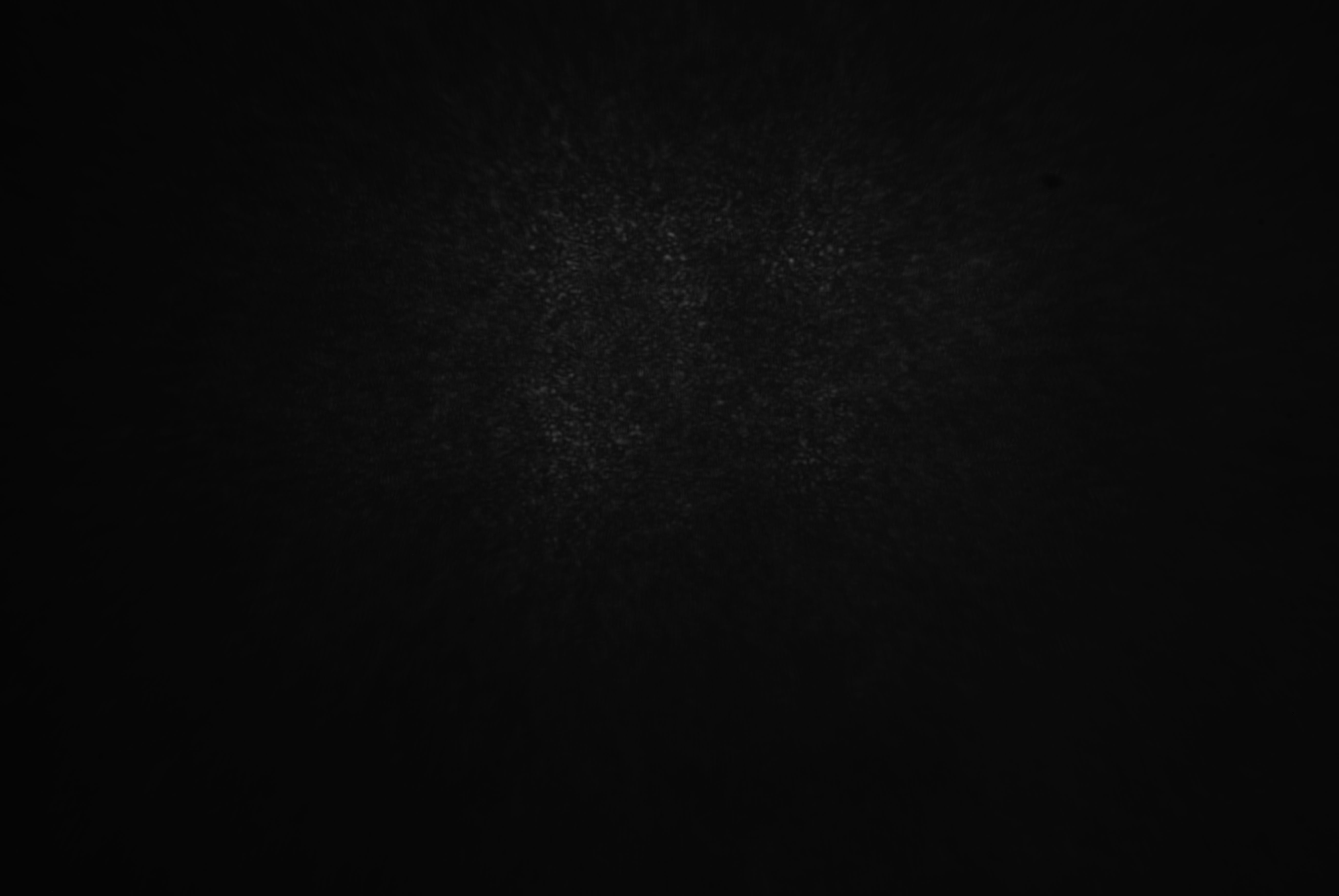

Supplement: Supplementary file 7 — Source Data [file 41467_2023_43674_MOESM7_ESM.zip › Source Data/Data 1/x (20).JPG]

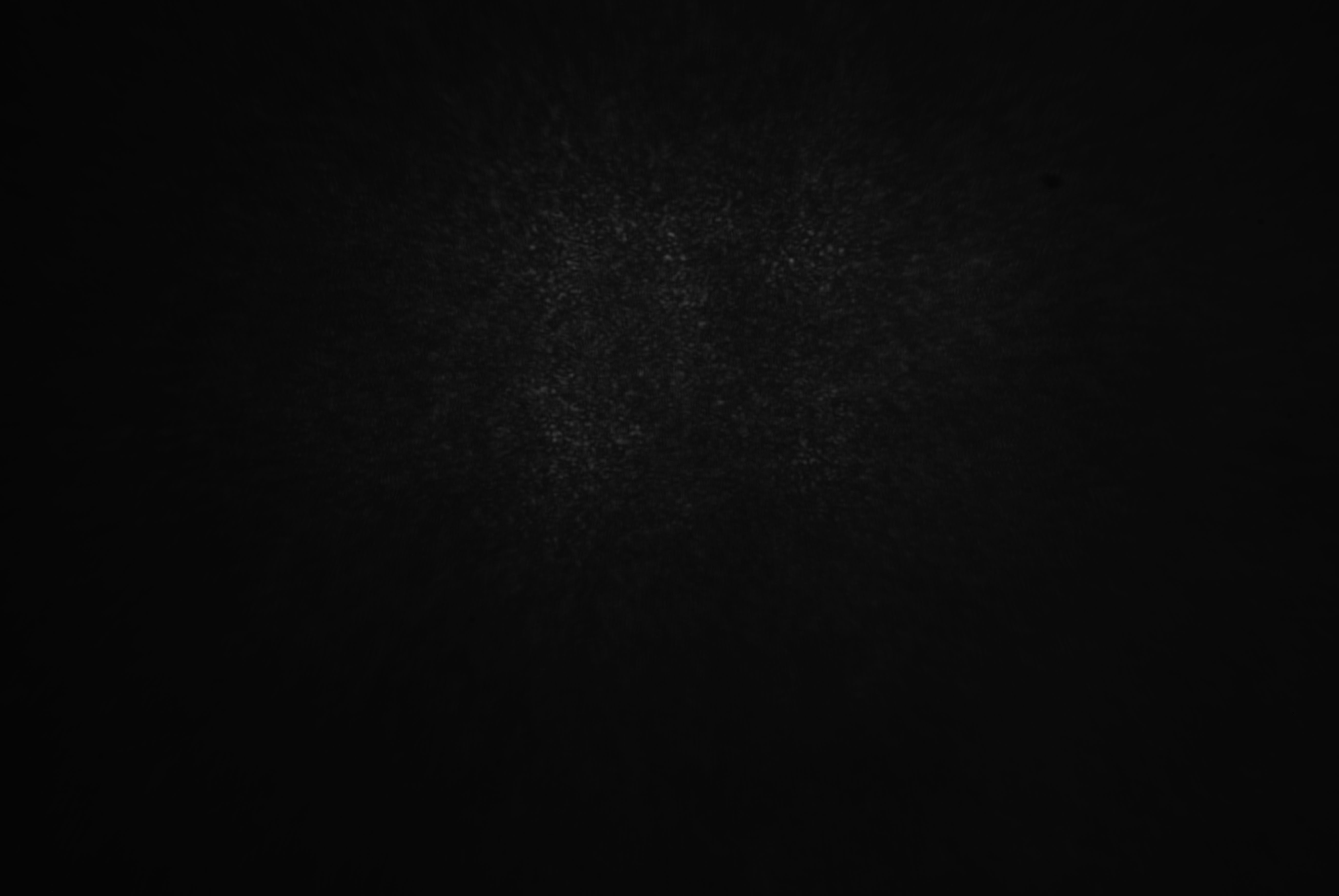

Supplement: Supplementary file 7 — Source Data [file 41467_2023_43674_MOESM7_ESM.zip › Source Data/Data 1/x (21).JPG]

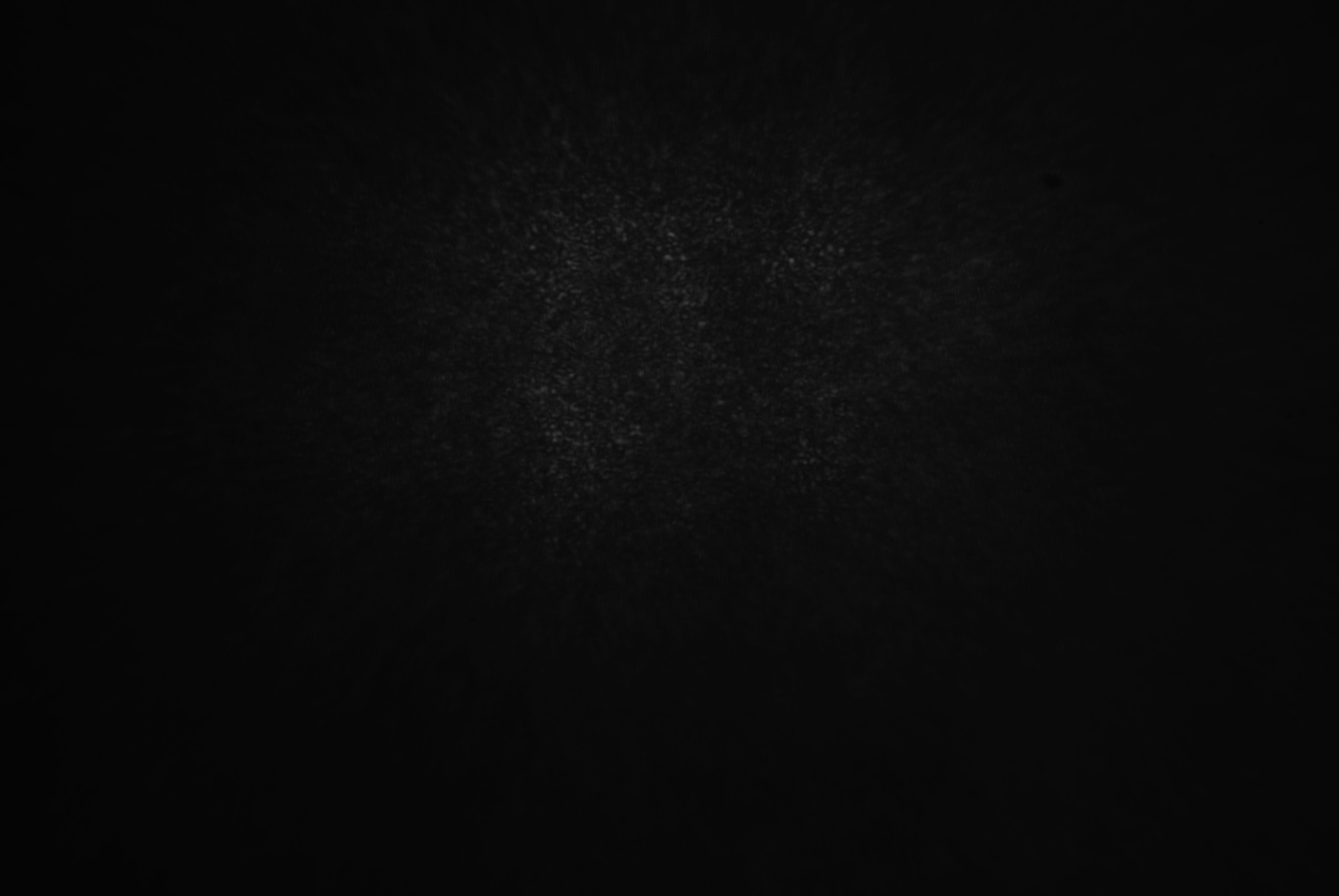

Supplement: Supplementary file 7 — Source Data [file 41467_2023_43674_MOESM7_ESM.zip › Source Data/Data 1/x (22).JPG]

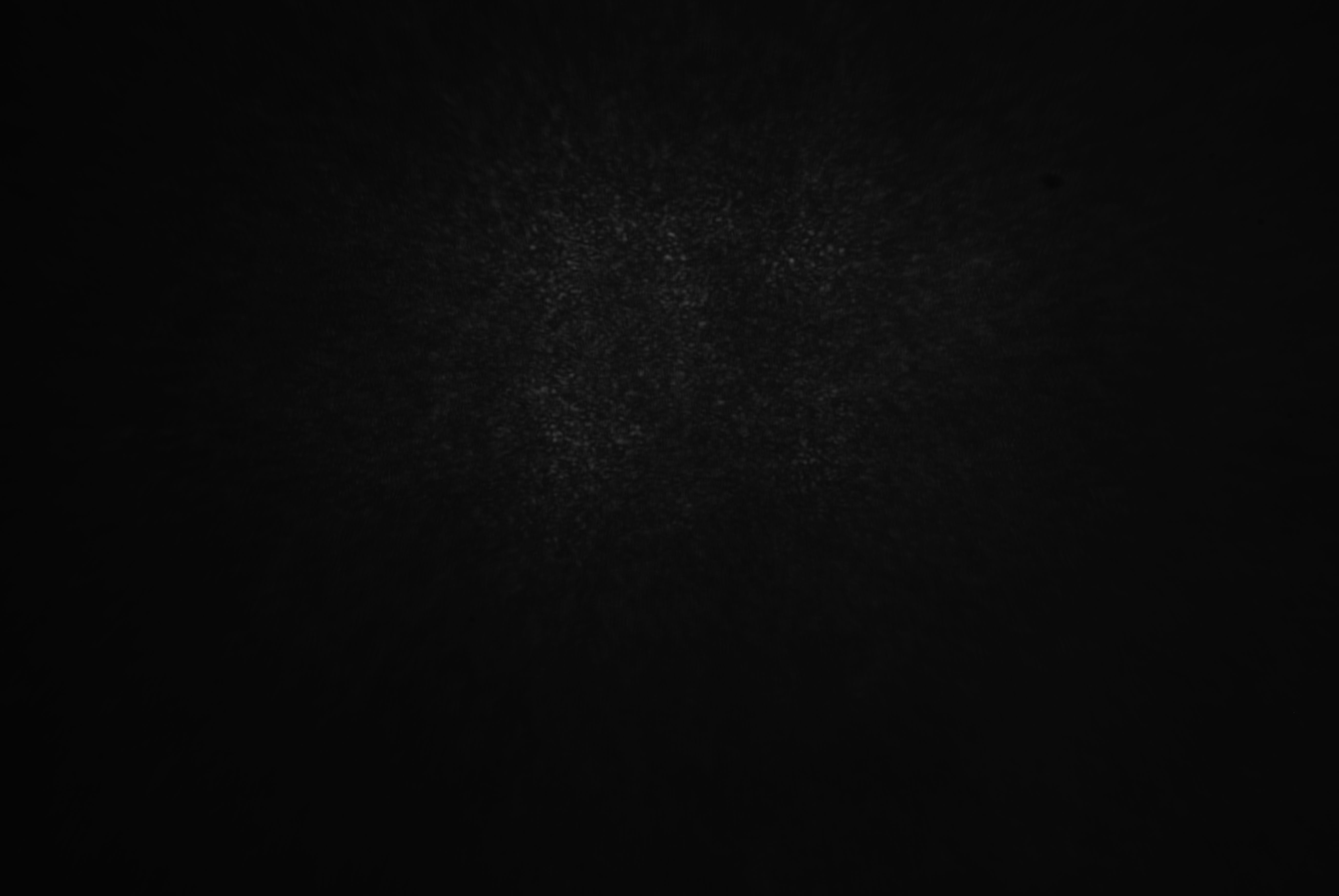

Supplement: Supplementary file 7 — Source Data [file 41467_2023_43674_MOESM7_ESM.zip › Source Data/Data 1/x (23).JPG]

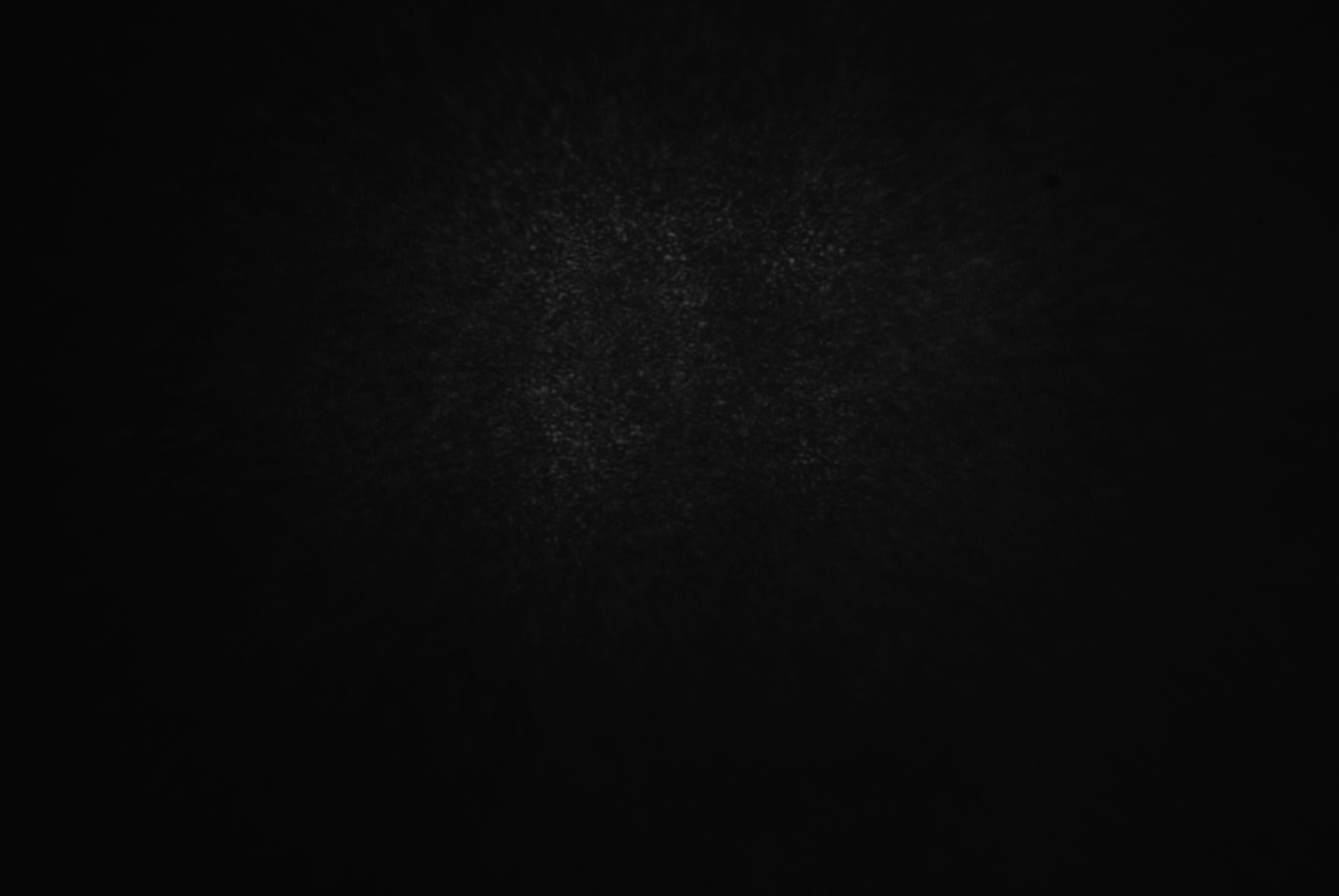

Supplement: Supplementary file 7 — Source Data [file 41467_2023_43674_MOESM7_ESM.zip › Source Data/Data 1/x (24).JPG]

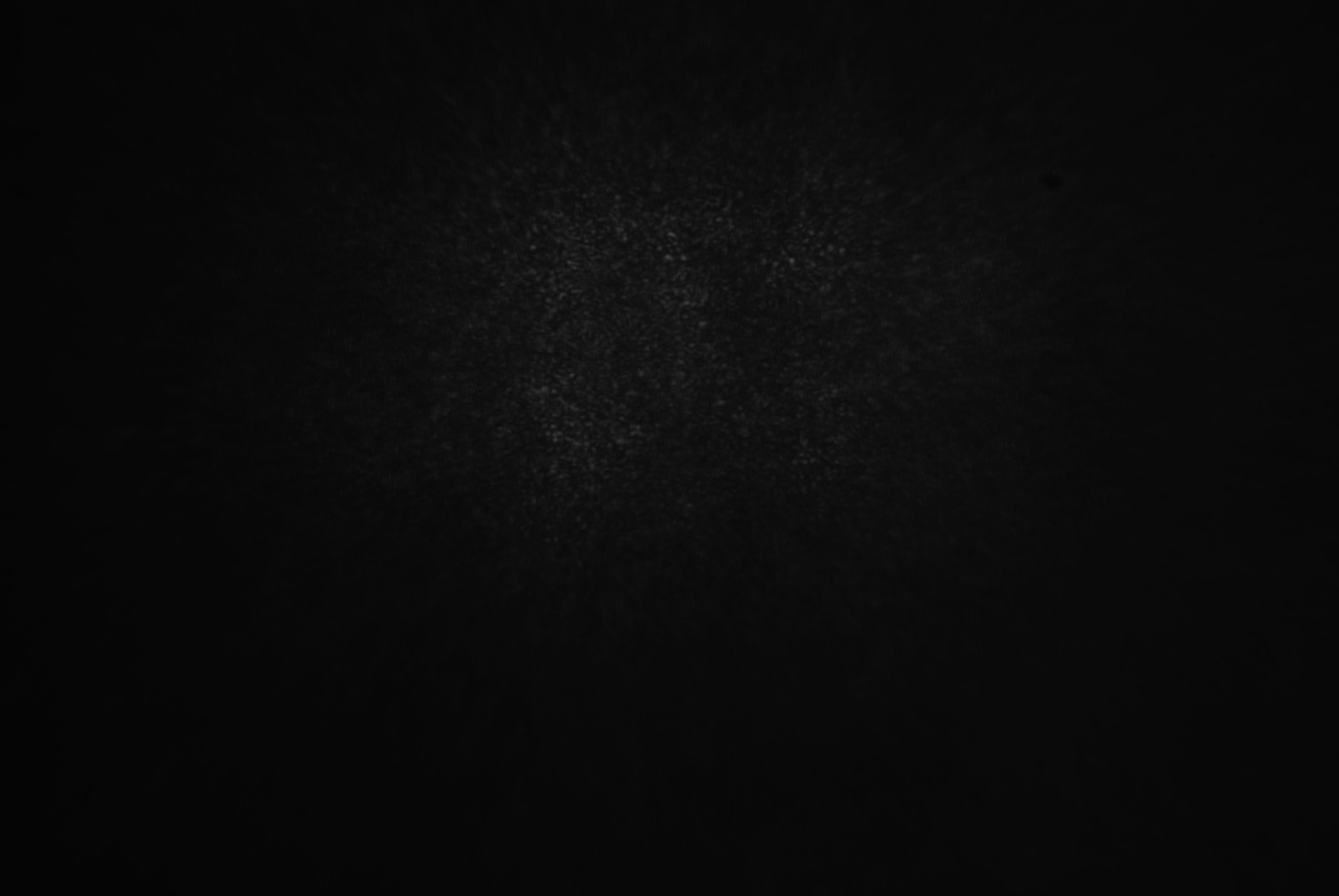

Supplement: Supplementary file 7 — Source Data [file 41467_2023_43674_MOESM7_ESM.zip › Source Data/Data 1/x (25).JPG]

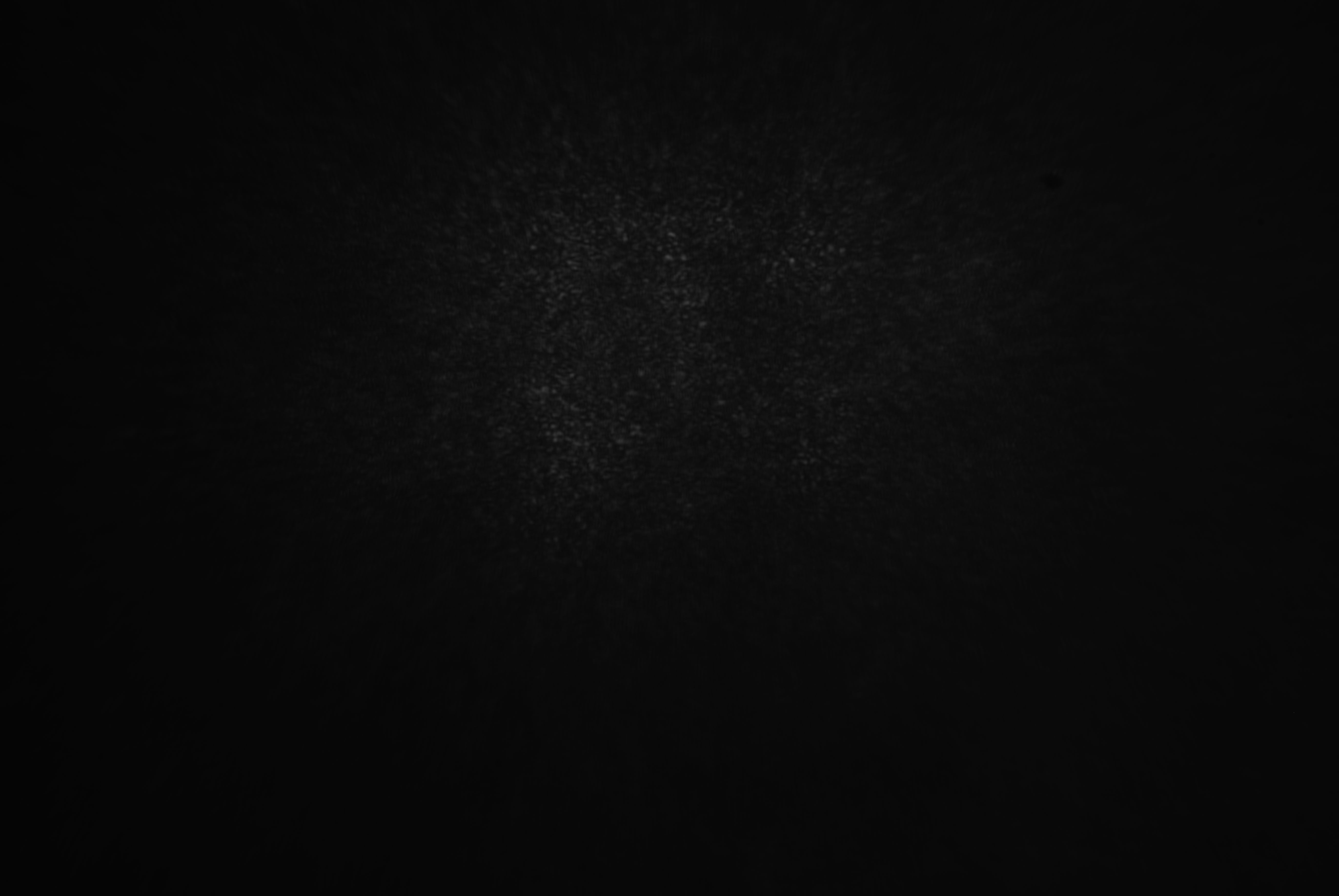

Supplement: Supplementary file 7 — Source Data [file 41467_2023_43674_MOESM7_ESM.zip › Source Data/Data 1/x (26).JPG]

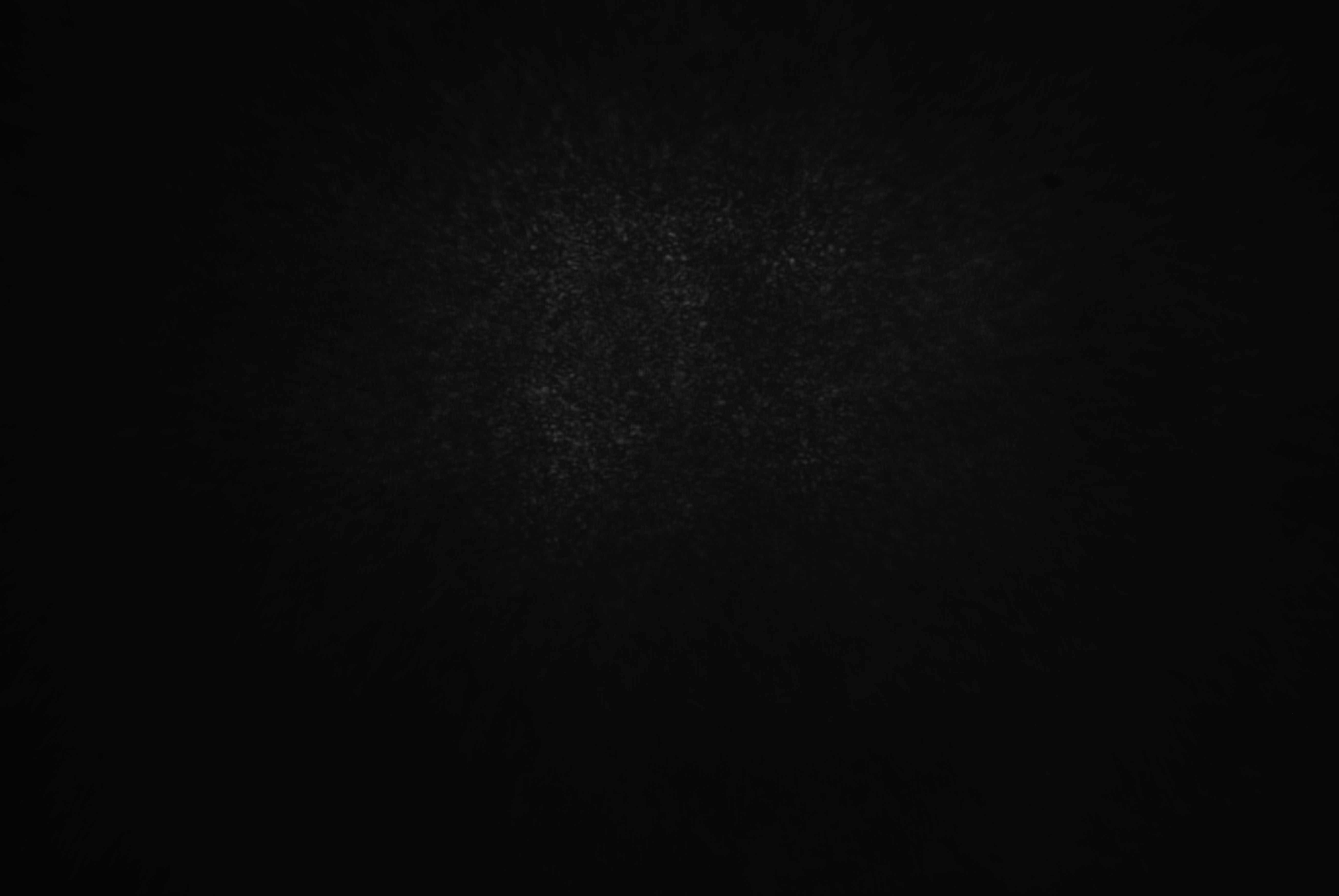

Supplement: Supplementary file 7 — Source Data [file 41467_2023_43674_MOESM7_ESM.zip › Source Data/Data 1/x (27).JPG]

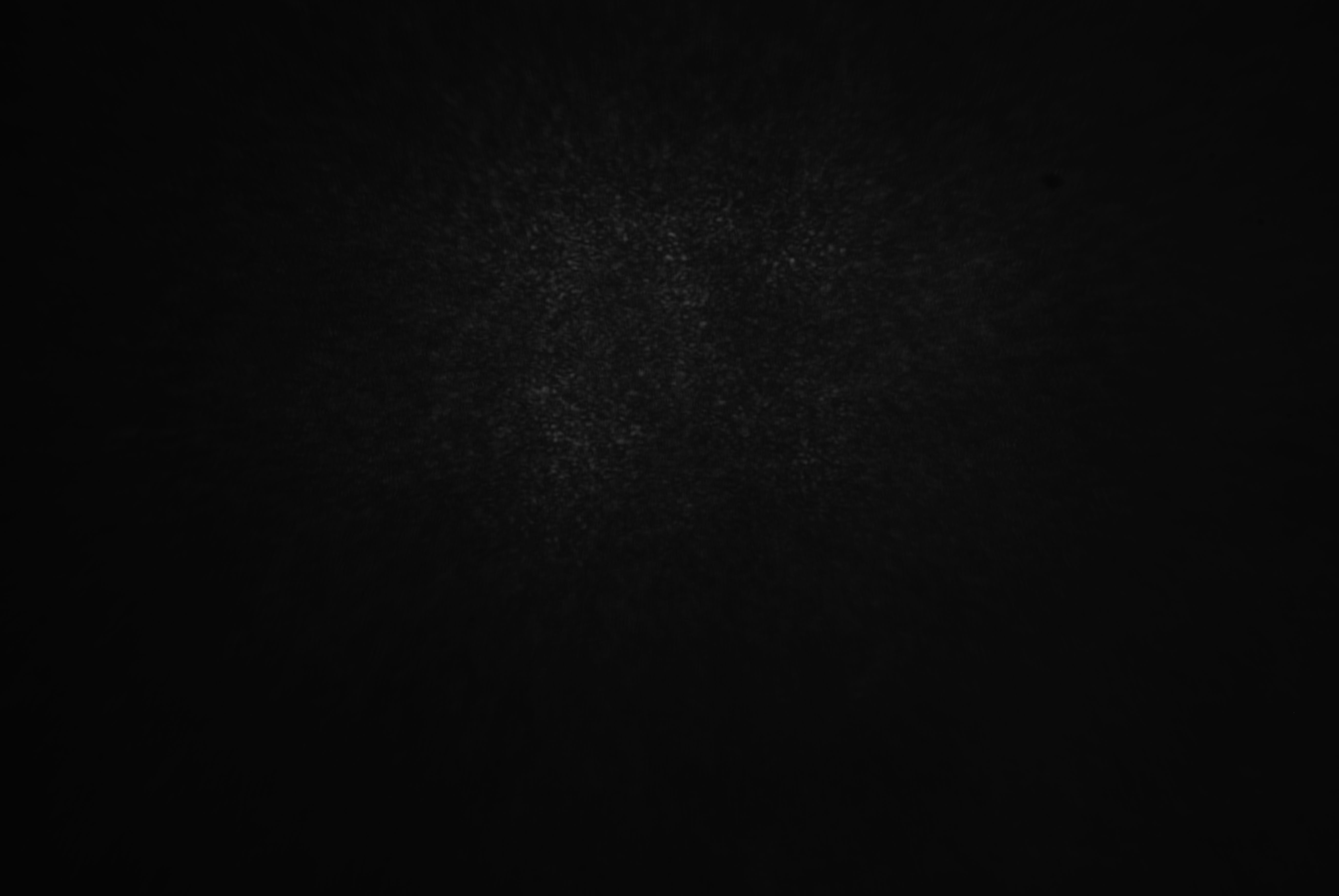

Supplement: Supplementary file 7 — Source Data [file 41467_2023_43674_MOESM7_ESM.zip › Source Data/Data 1/x (28).JPG]

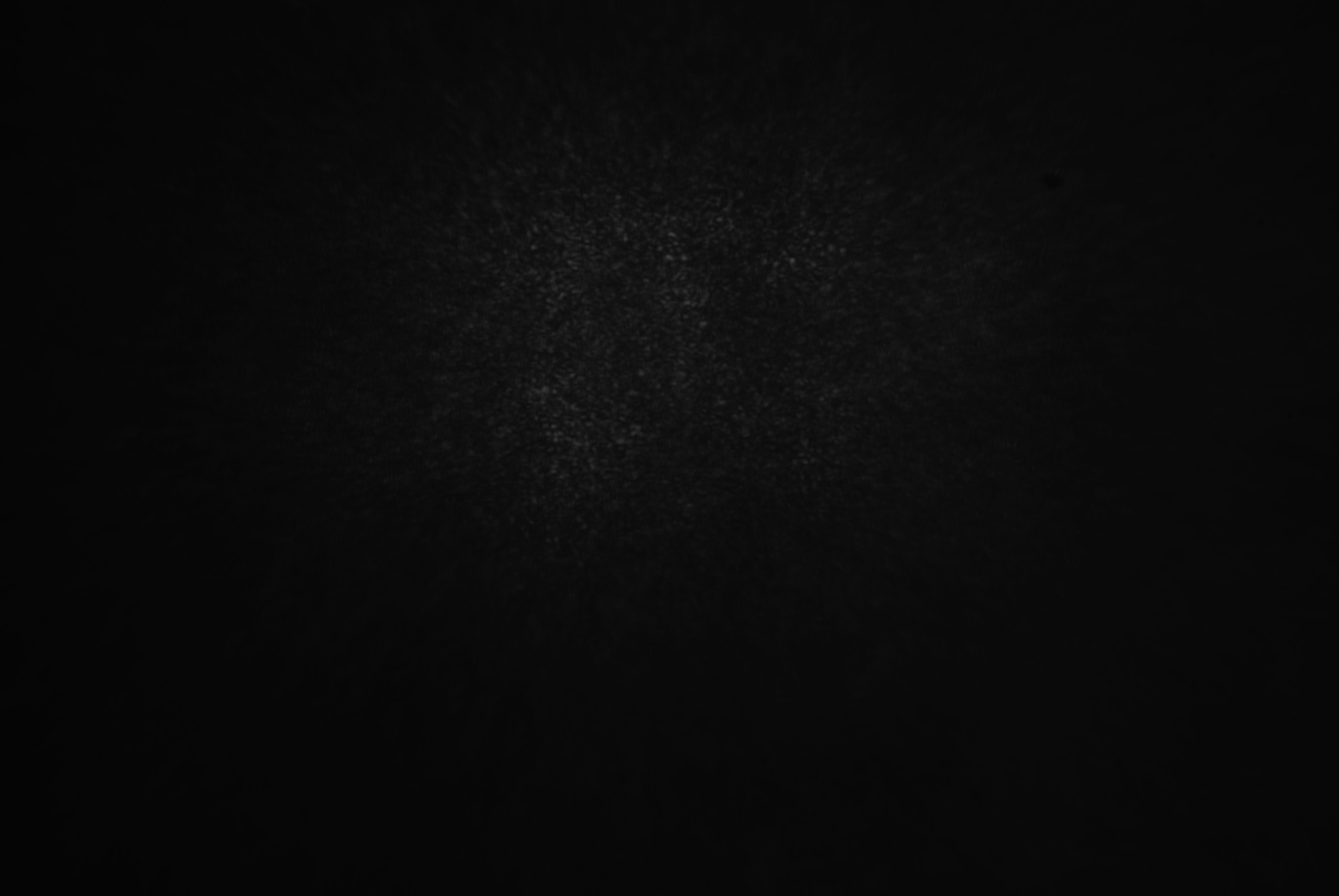

Supplement: Supplementary file 7 — Source Data [file 41467_2023_43674_MOESM7_ESM.zip › Source Data/Data 1/x (29).JPG]

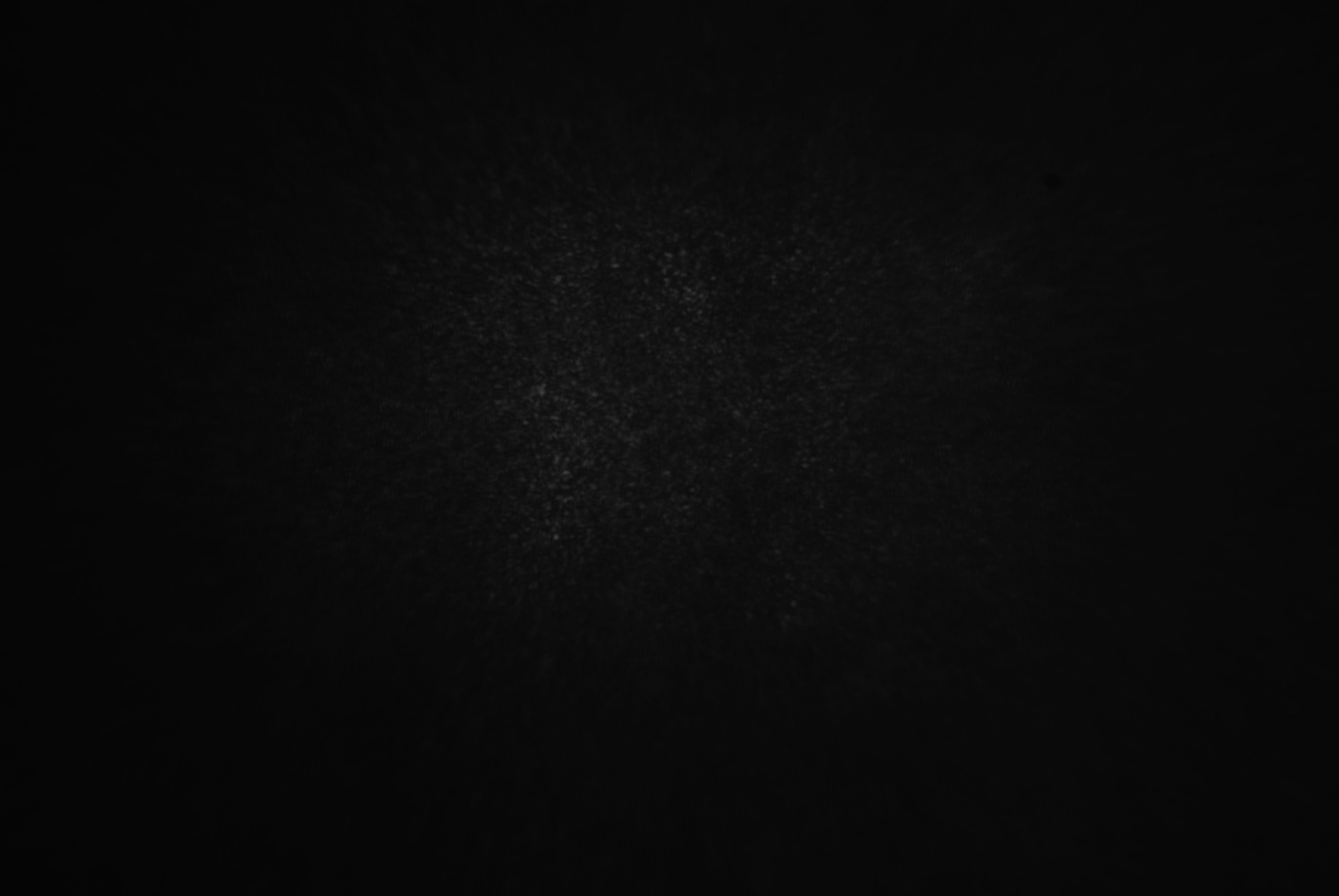

Supplement: Supplementary file 7 — Source Data [file 41467_2023_43674_MOESM7_ESM.zip › Source Data/Data 2/y (30).JPG]

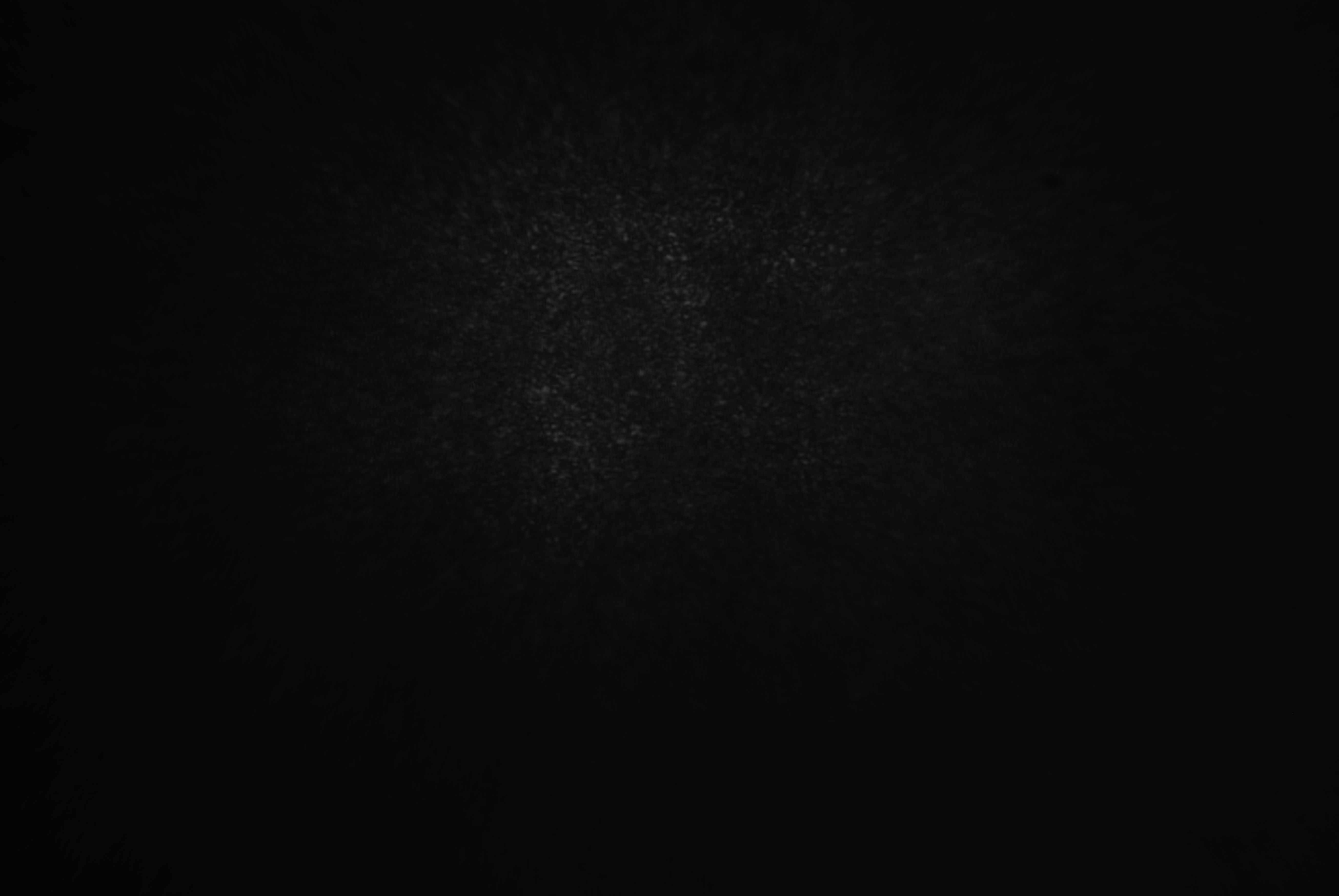

Supplement: Supplementary file 7 — Source Data [file 41467_2023_43674_MOESM7_ESM.zip › Source Data/Data 2/y (1).JPG]

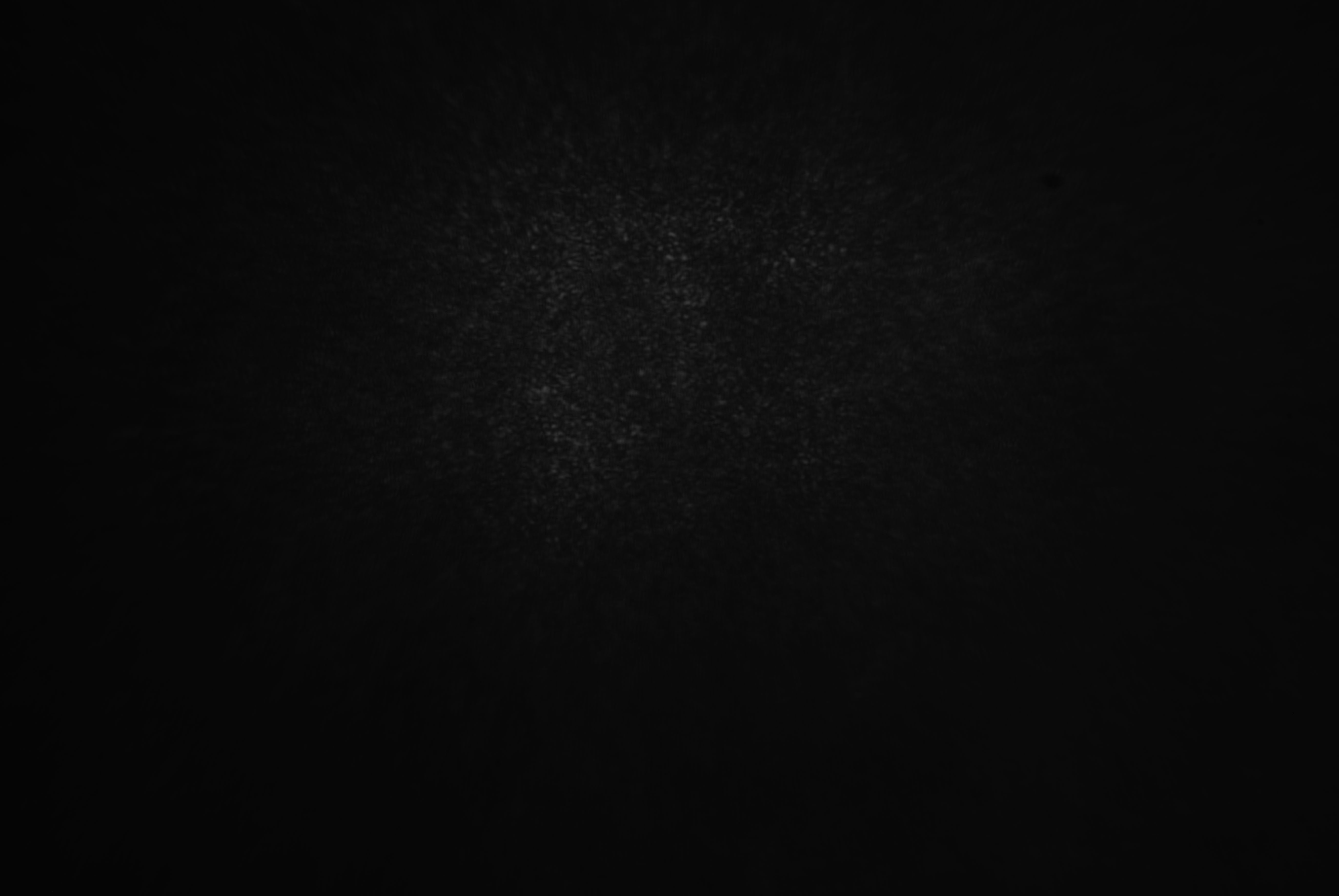

Supplement: Supplementary file 7 — Source Data [file 41467_2023_43674_MOESM7_ESM.zip › Source Data/Data 2/y (2).JPG]

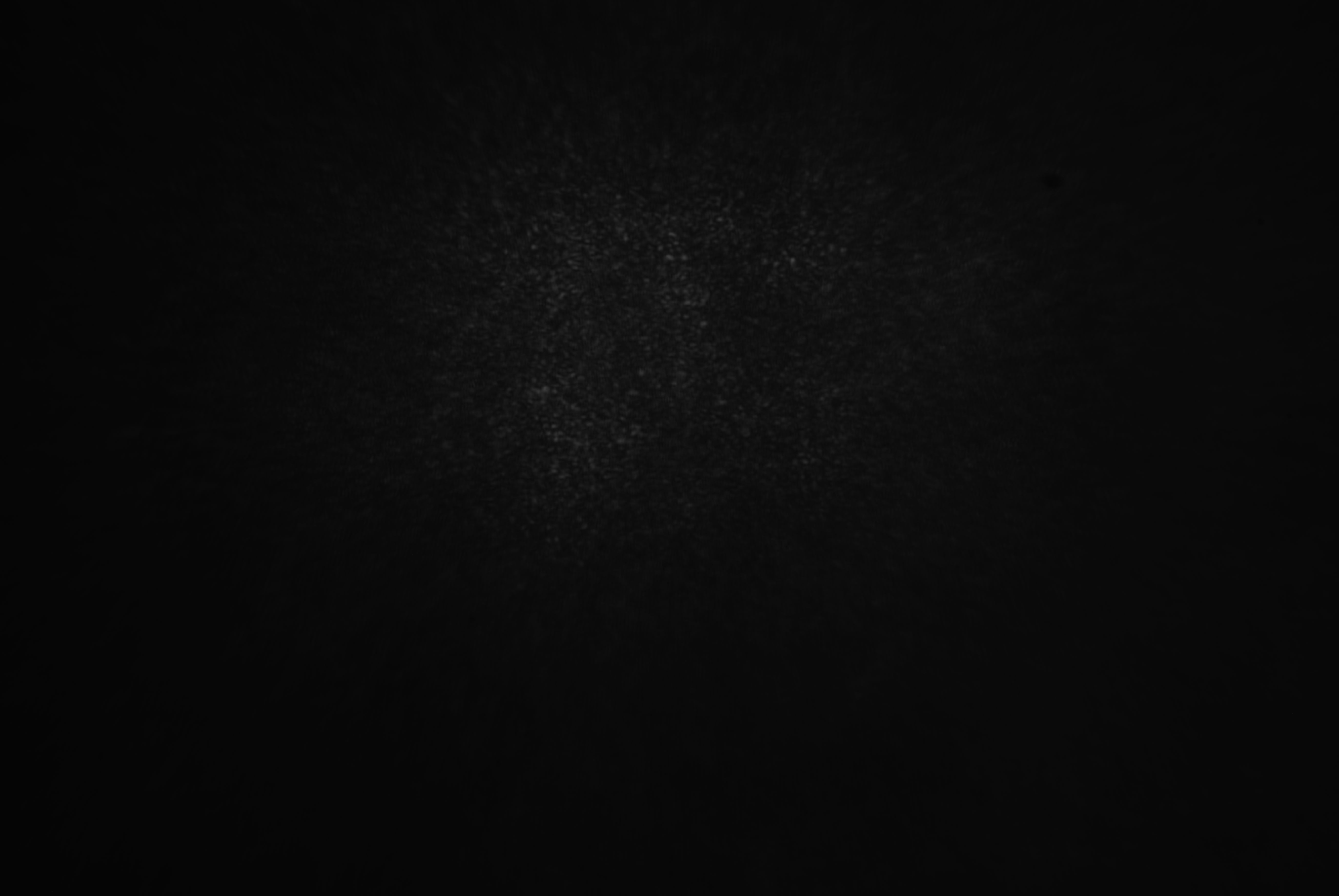

Supplement: Supplementary file 7 — Source Data [file 41467_2023_43674_MOESM7_ESM.zip › Source Data/Data 2/y (3).JPG]

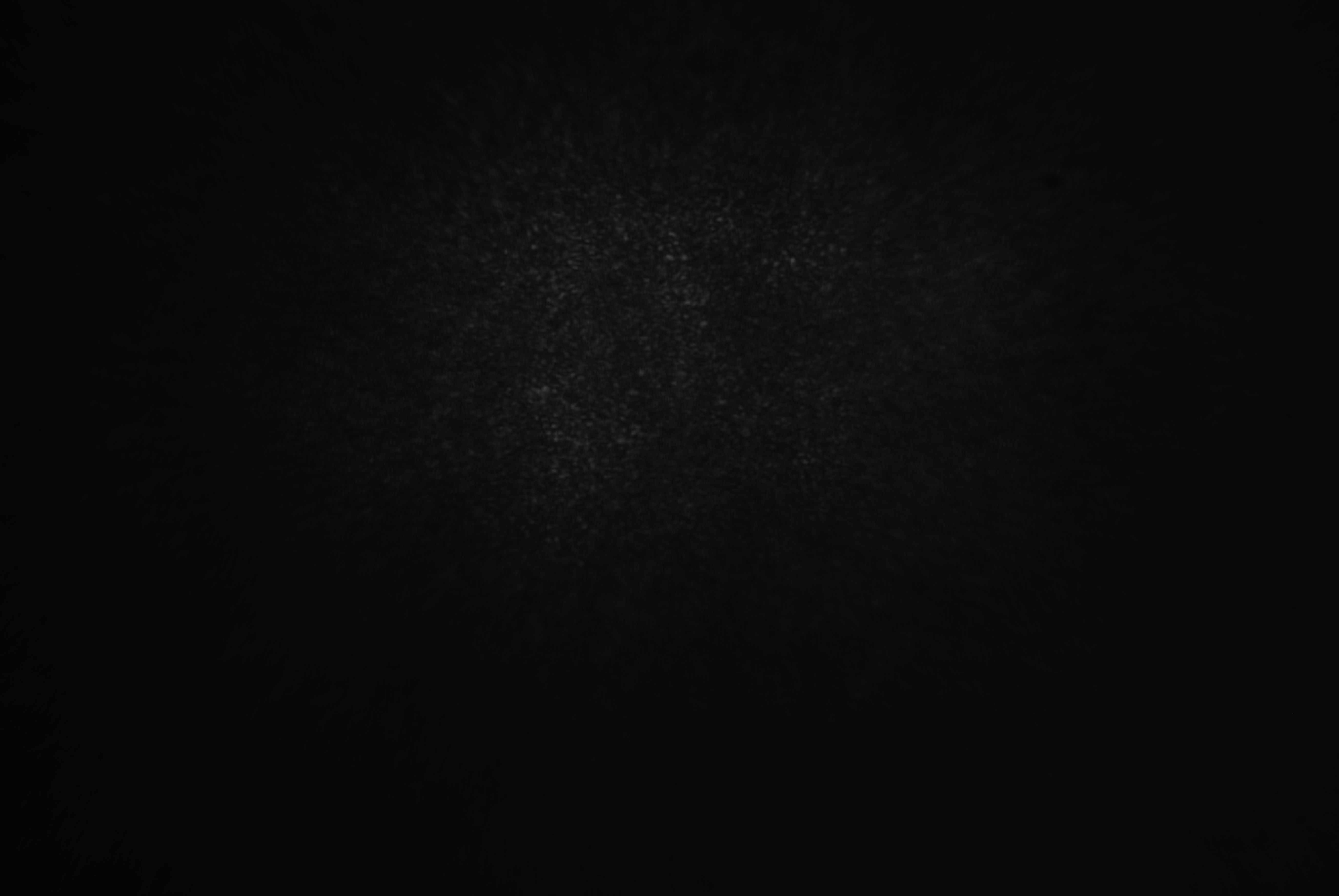

Supplement: Supplementary file 7 — Source Data [file 41467_2023_43674_MOESM7_ESM.zip › Source Data/Data 2/y (4).JPG]

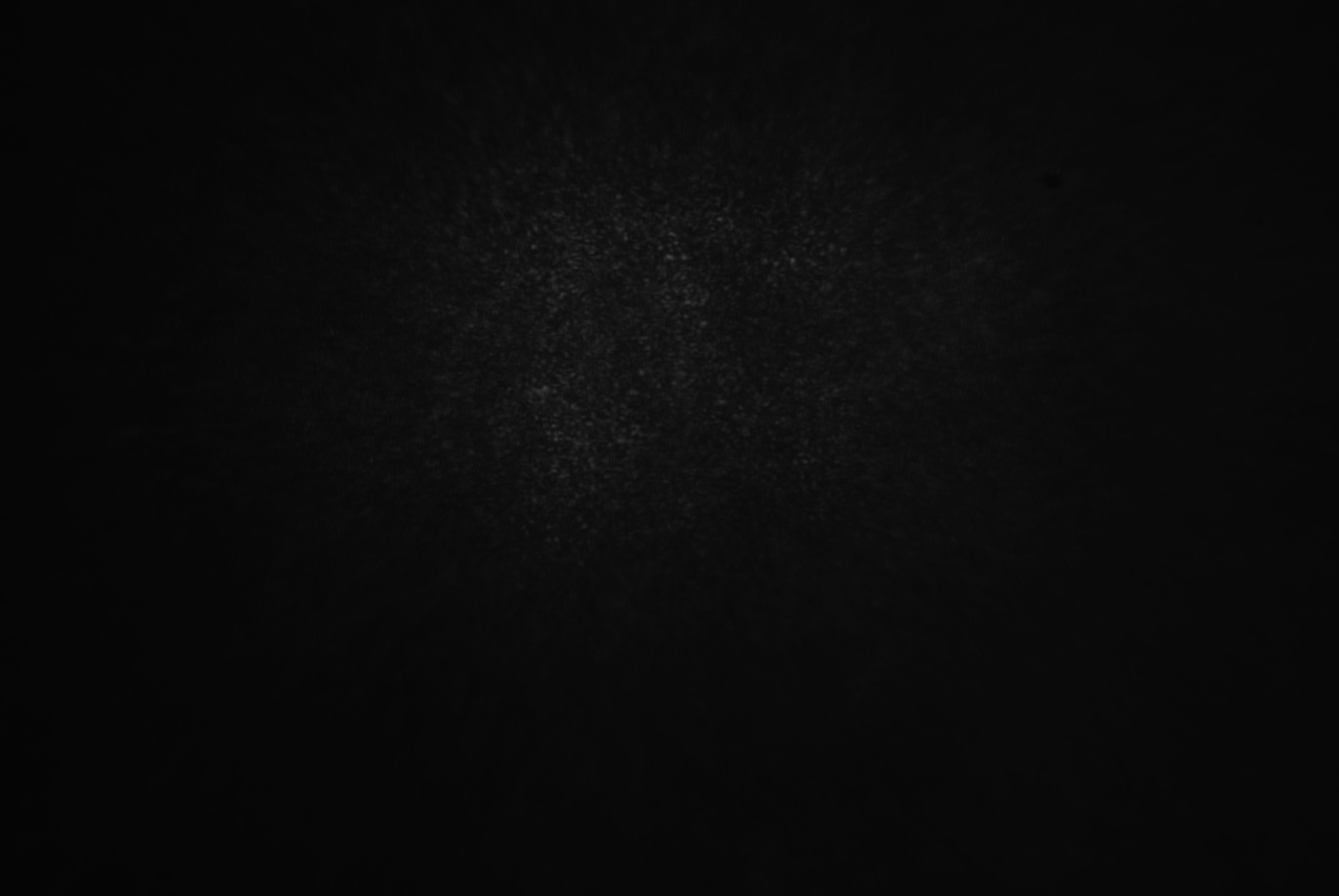

Supplement: Supplementary file 7 — Source Data [file 41467_2023_43674_MOESM7_ESM.zip › Source Data/Data 2/y (5).JPG]

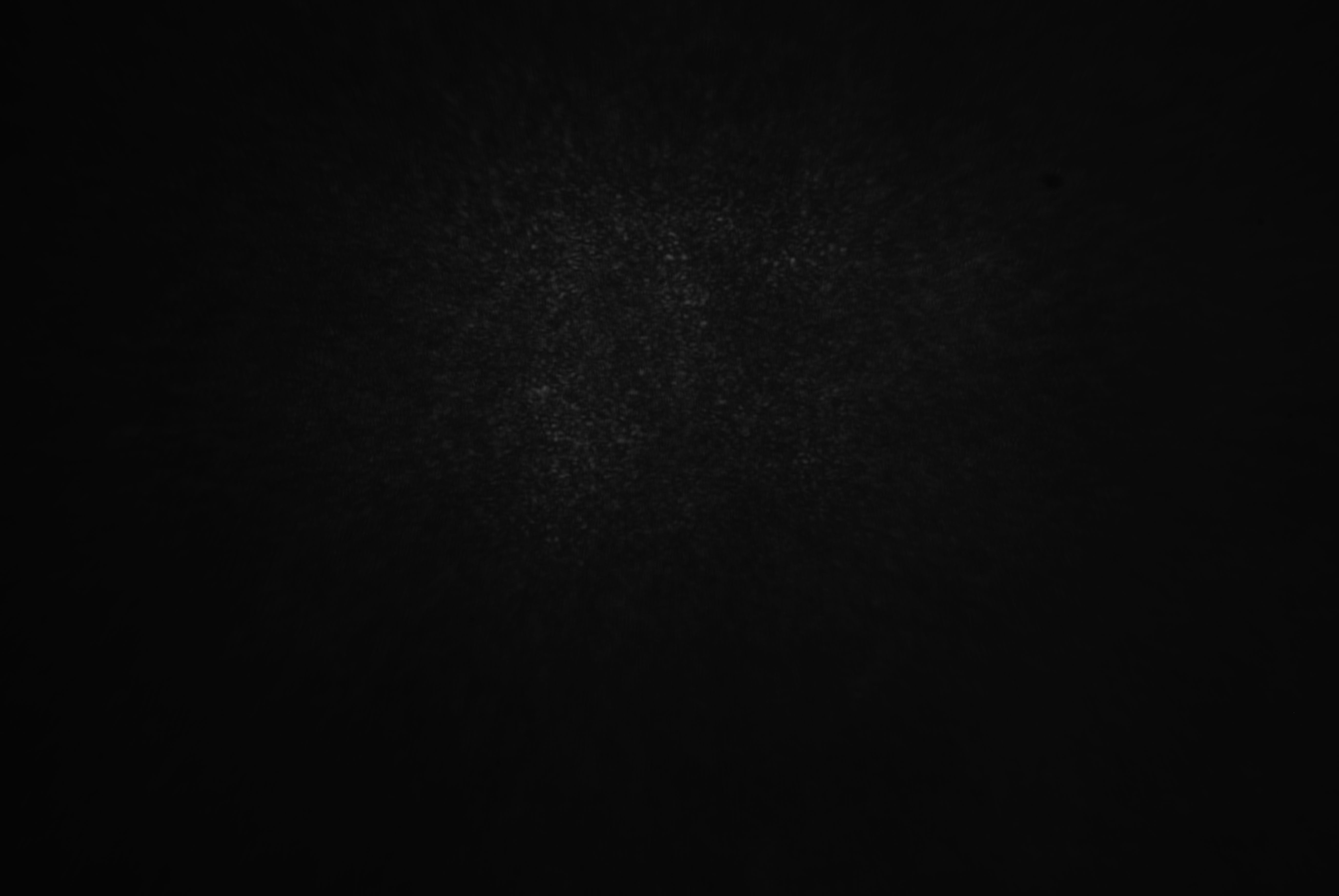

Supplement: Supplementary file 7 — Source Data [file 41467_2023_43674_MOESM7_ESM.zip › Source Data/Data 2/y (6).JPG]

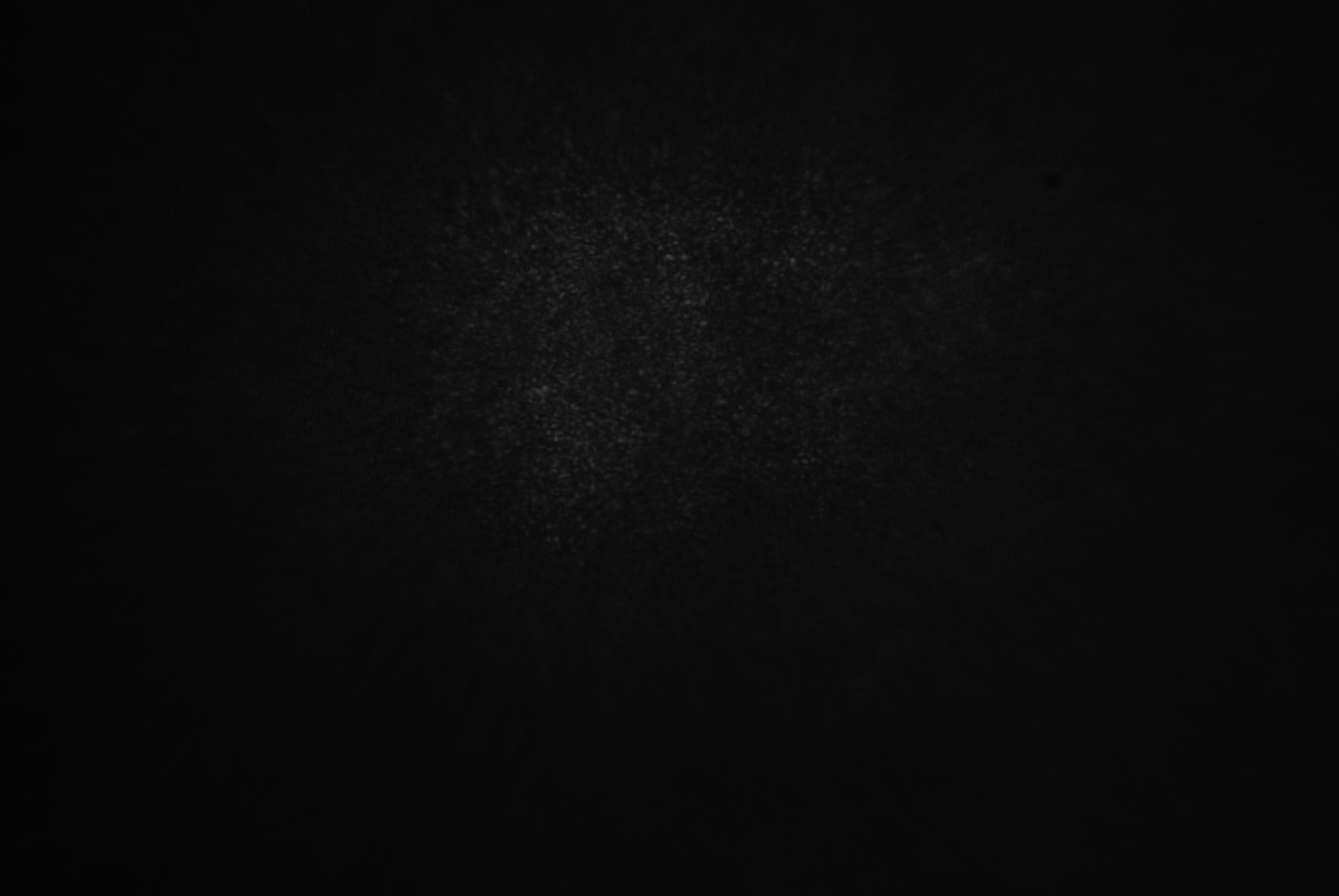

Supplement: Supplementary file 7 — Source Data [file 41467_2023_43674_MOESM7_ESM.zip › Source Data/Data 2/y (7).JPG]

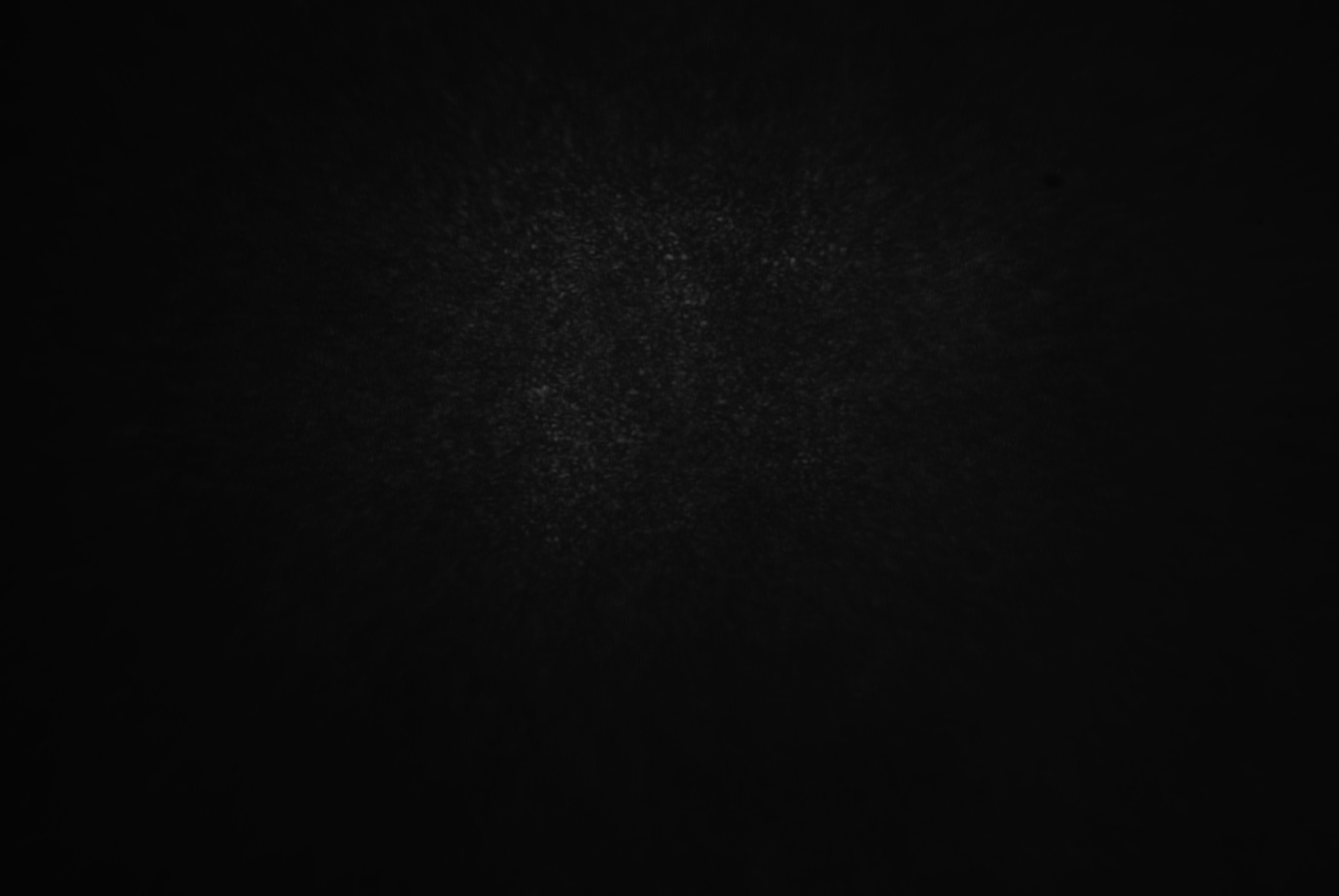

Supplement: Supplementary file 7 — Source Data [file 41467_2023_43674_MOESM7_ESM.zip › Source Data/Data 2/y (8).JPG]

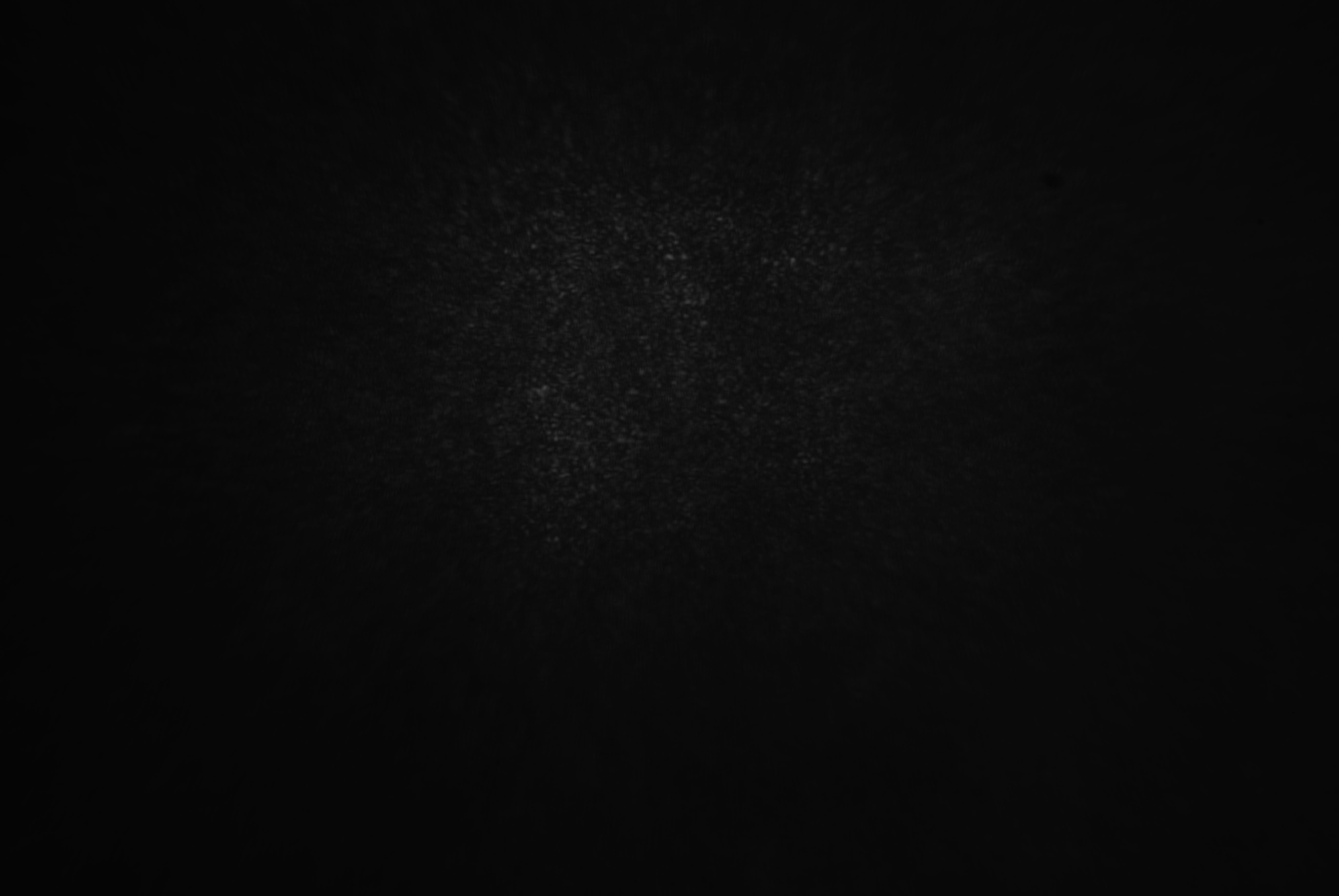

Supplement: Supplementary file 7 — Source Data [file 41467_2023_43674_MOESM7_ESM.zip › Source Data/Data 2/y (9).JPG]

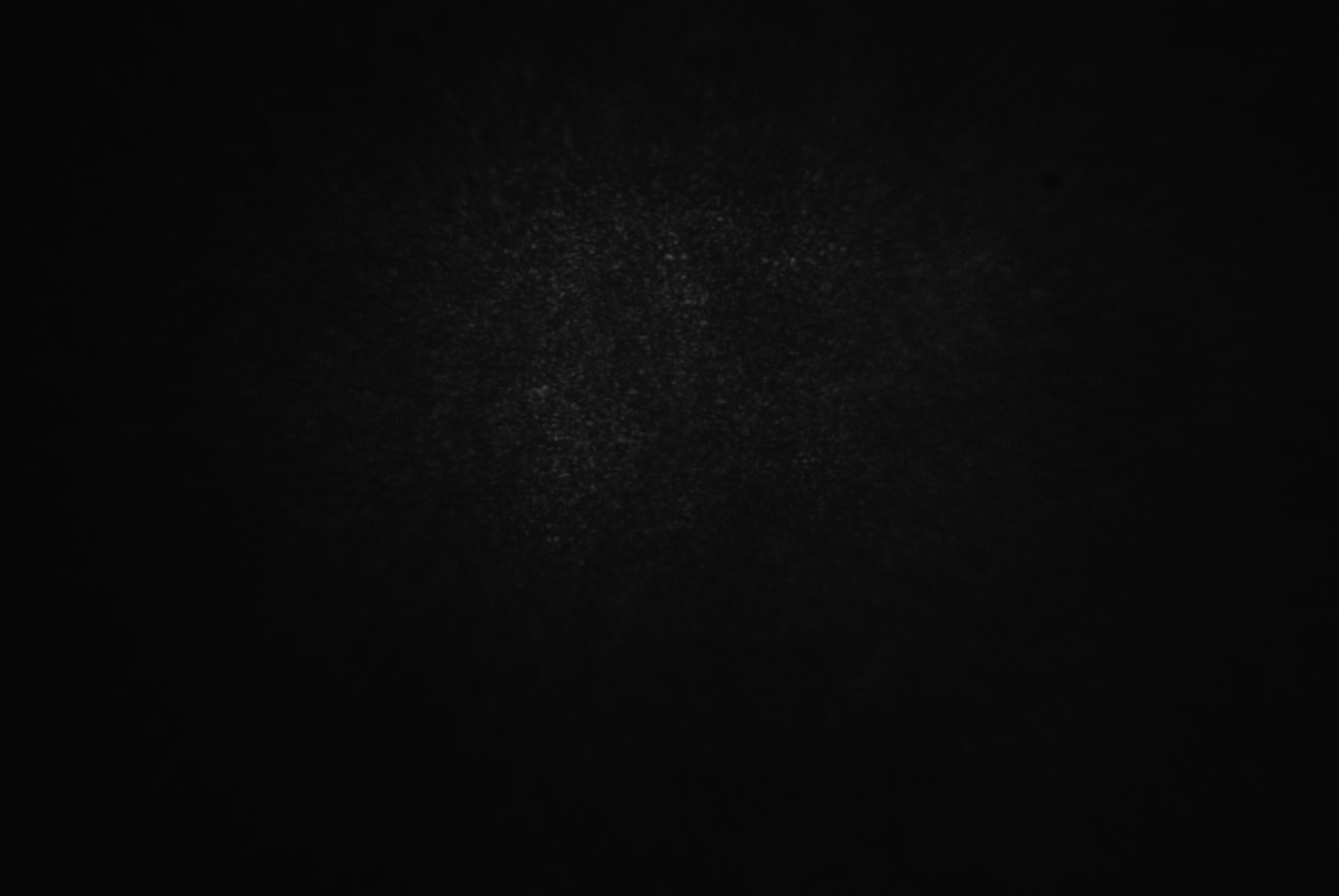

Supplement: Supplementary file 7 — Source Data [file 41467_2023_43674_MOESM7_ESM.zip › Source Data/Data 2/y (10).JPG]

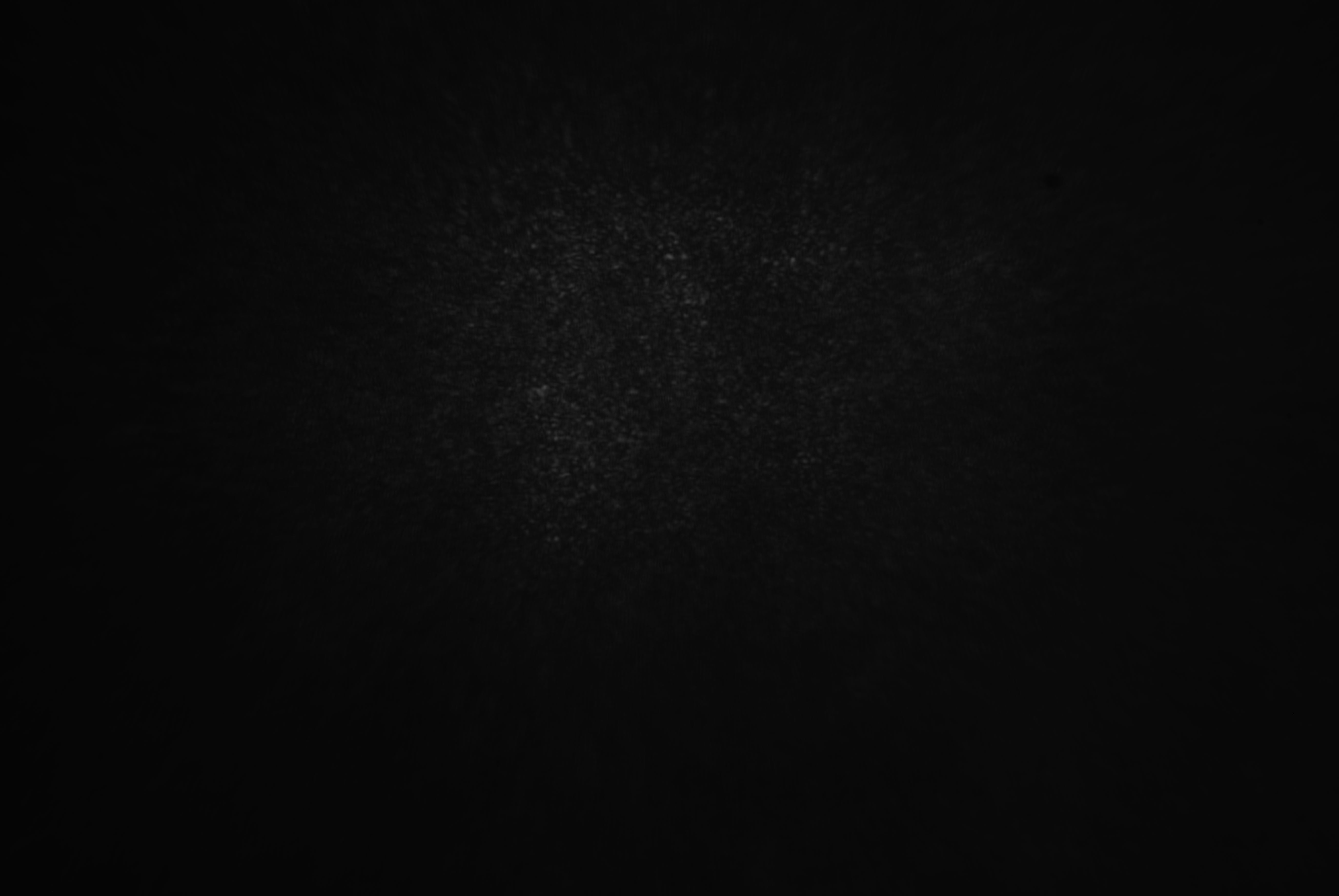

Supplement: Supplementary file 7 — Source Data [file 41467_2023_43674_MOESM7_ESM.zip › Source Data/Data 2/y (11).JPG]

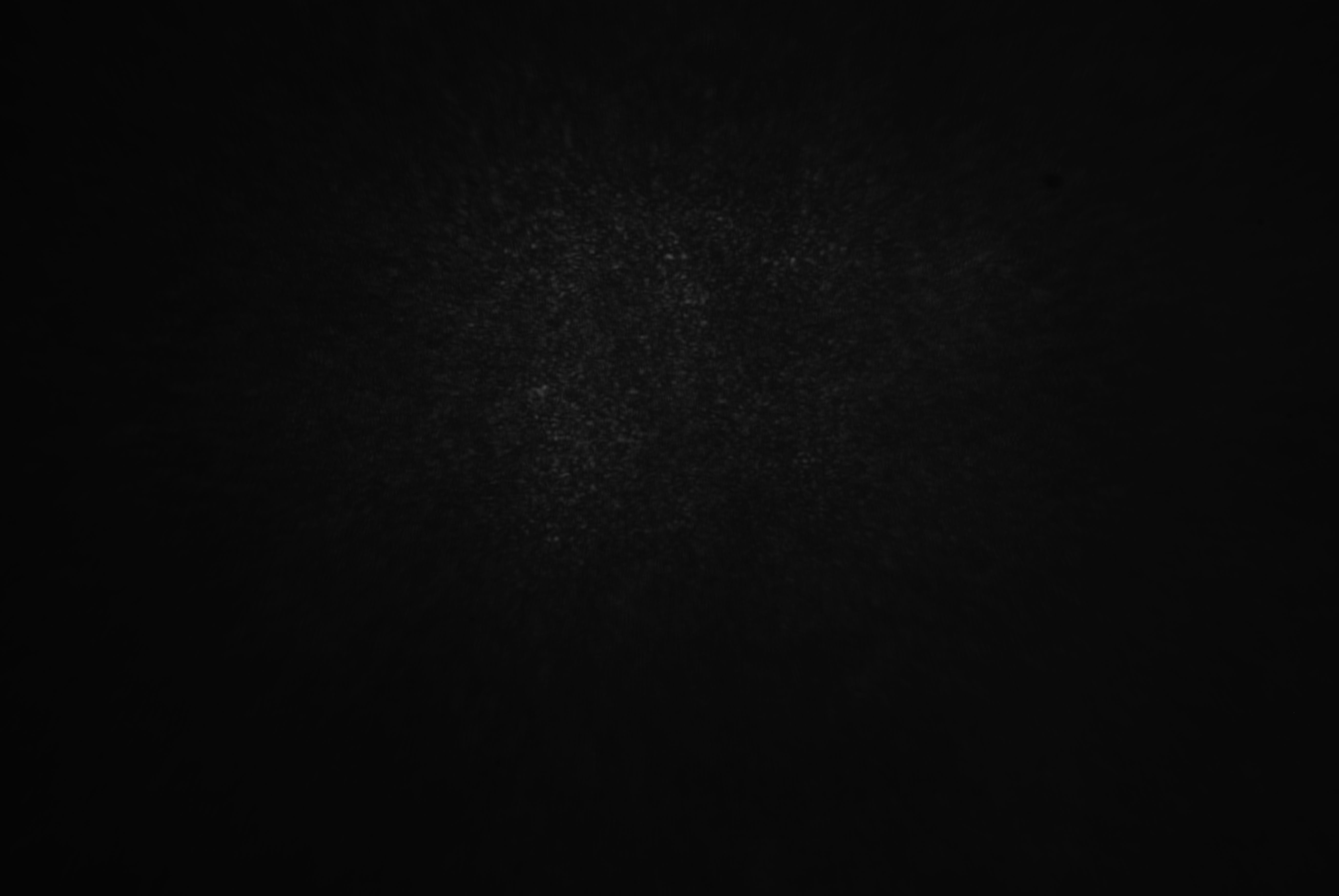

Supplement: Supplementary file 7 — Source Data [file 41467_2023_43674_MOESM7_ESM.zip › Source Data/Data 2/y (12).JPG]

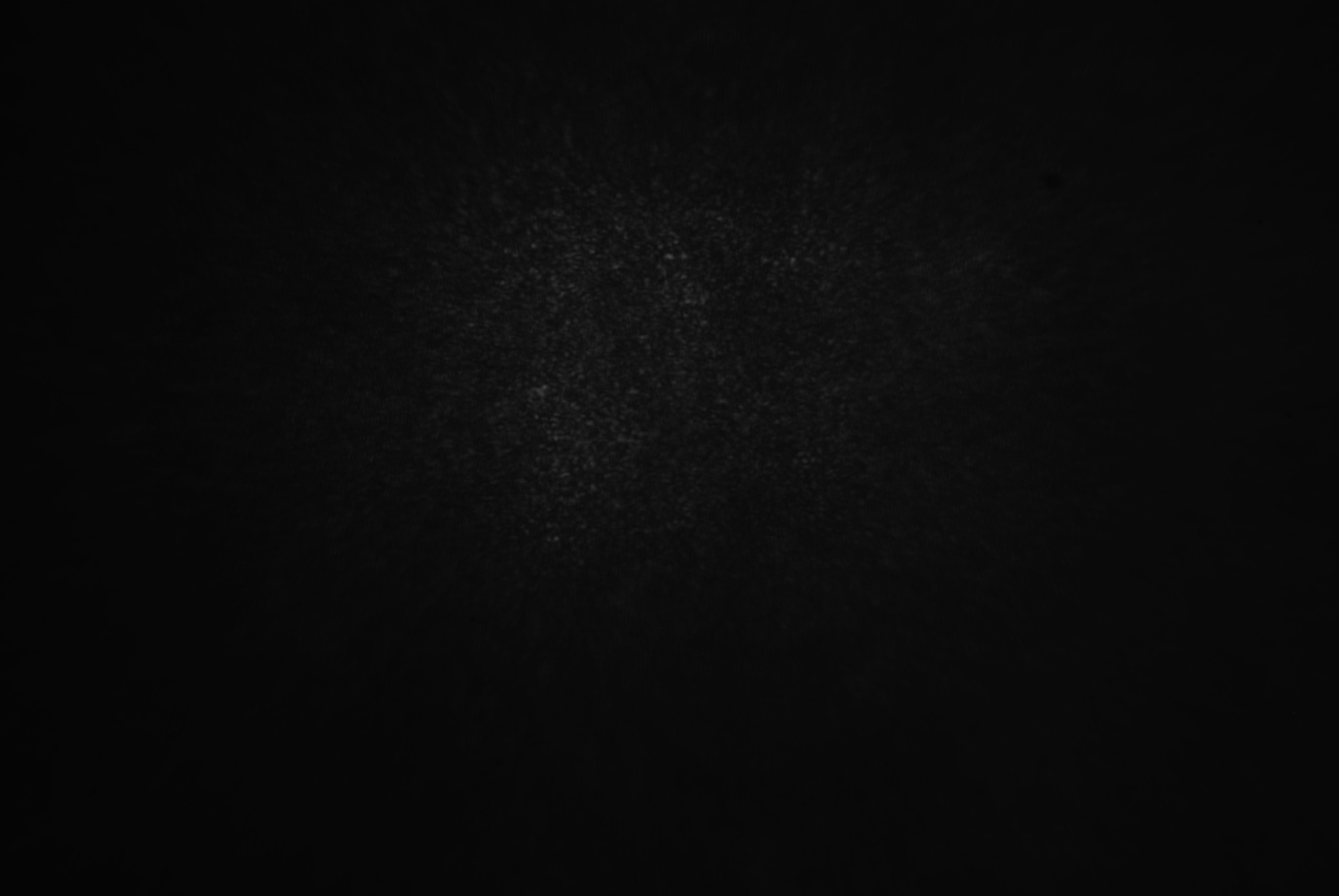

Supplement: Supplementary file 7 — Source Data [file 41467_2023_43674_MOESM7_ESM.zip › Source Data/Data 2/y (13).JPG]

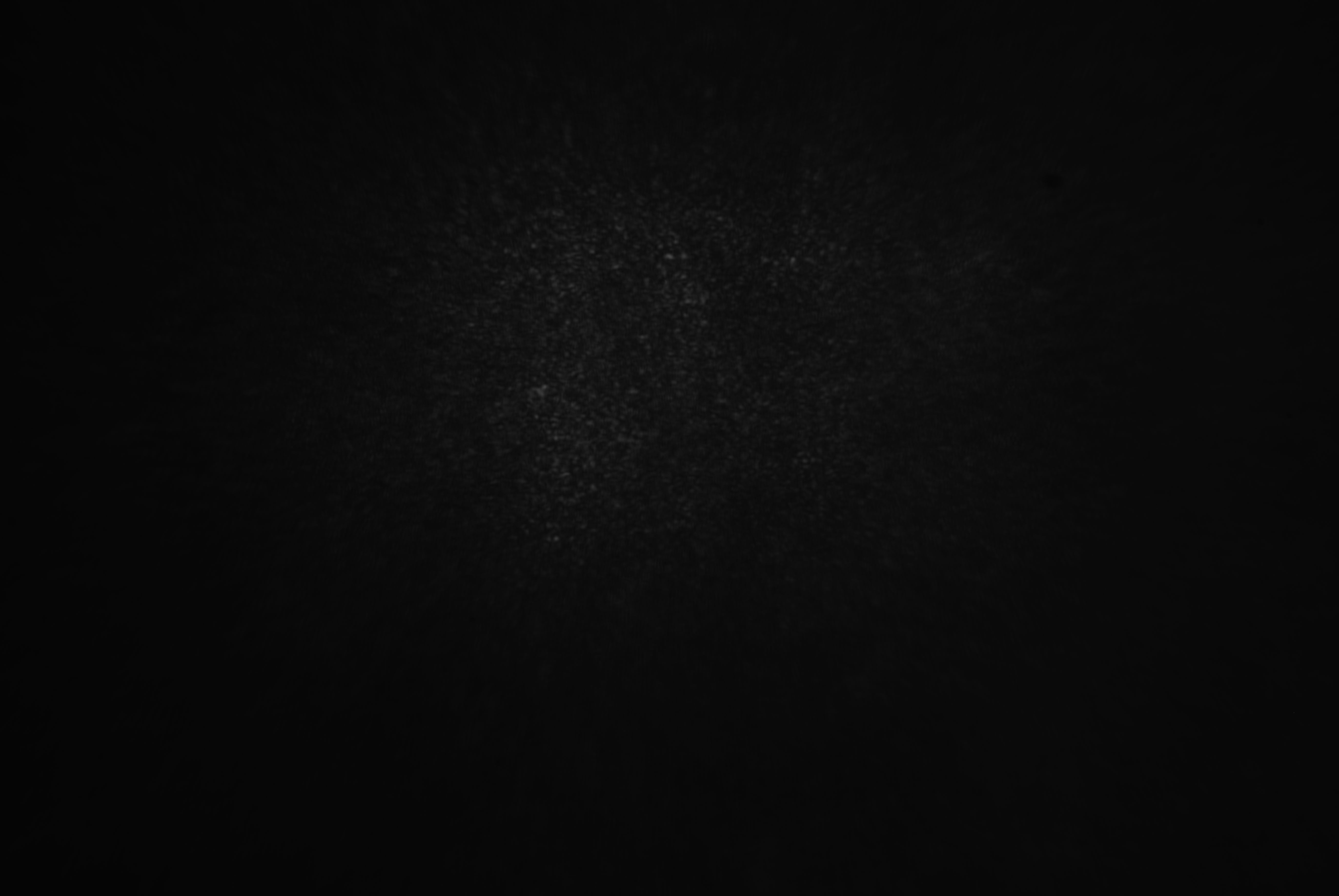

Supplement: Supplementary file 7 — Source Data [file 41467_2023_43674_MOESM7_ESM.zip › Source Data/Data 2/y (14).JPG]

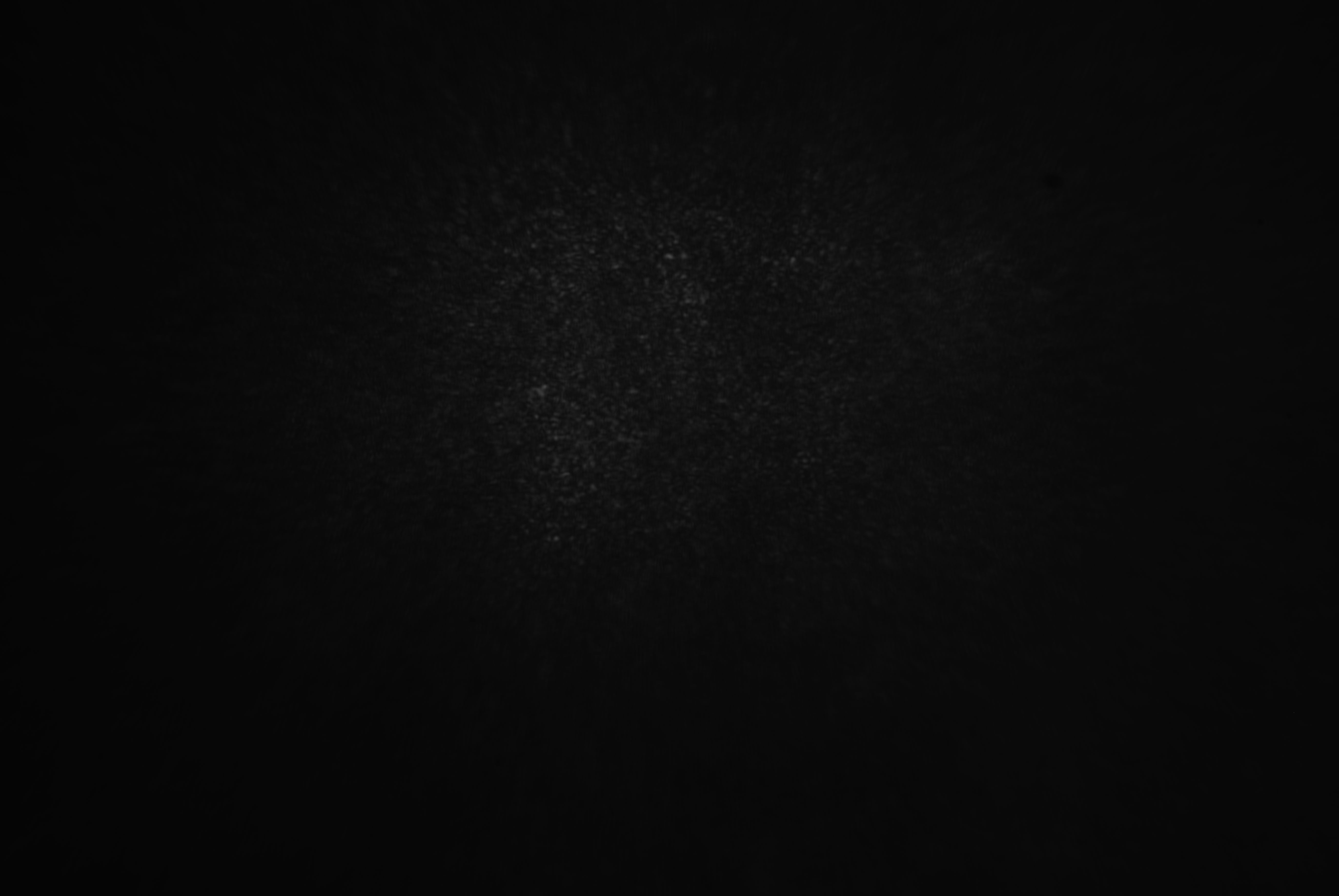

Supplement: Supplementary file 7 — Source Data [file 41467_2023_43674_MOESM7_ESM.zip › Source Data/Data 2/y (15).JPG]

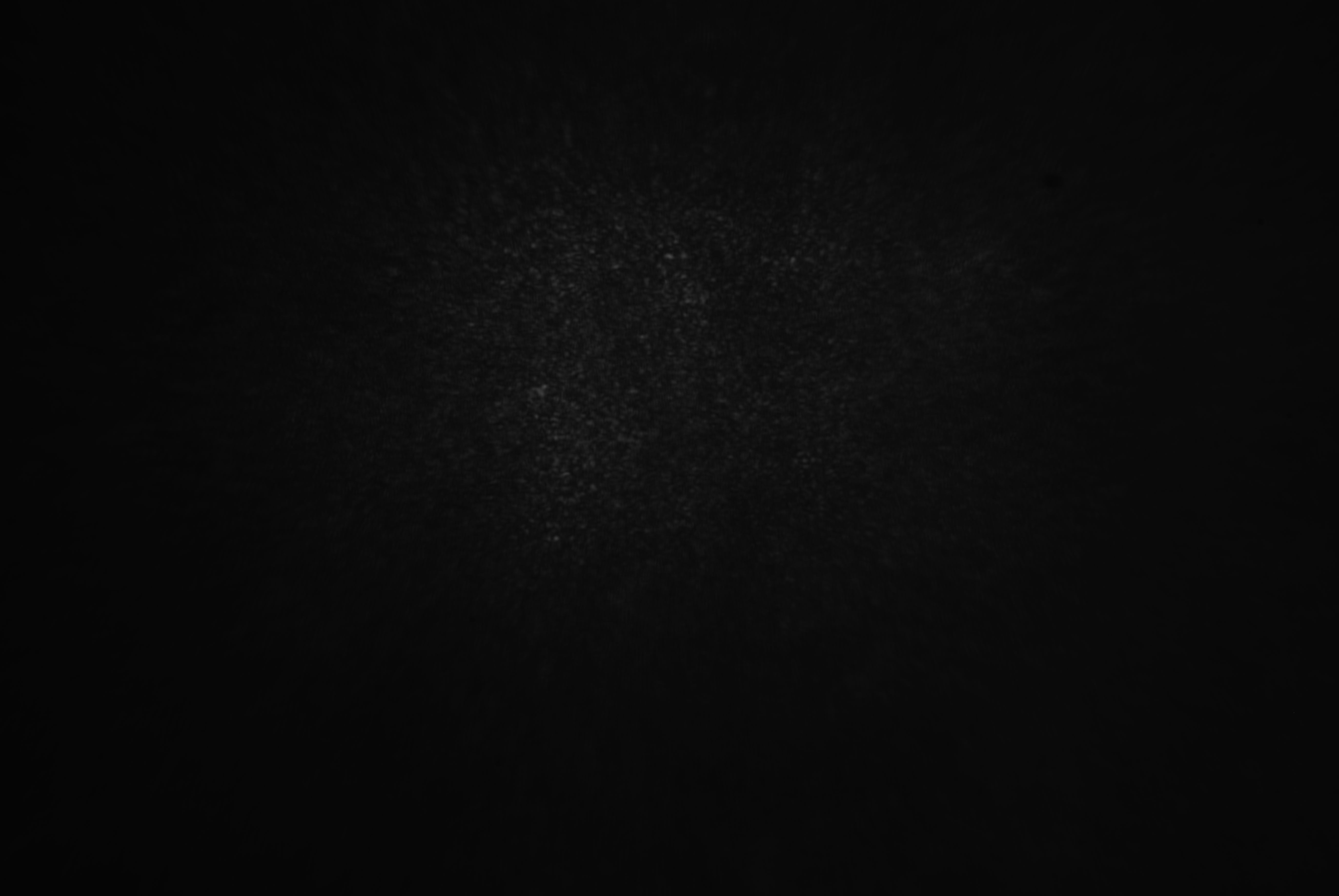

Supplement: Supplementary file 7 — Source Data [file 41467_2023_43674_MOESM7_ESM.zip › Source Data/Data 2/y (16).JPG]

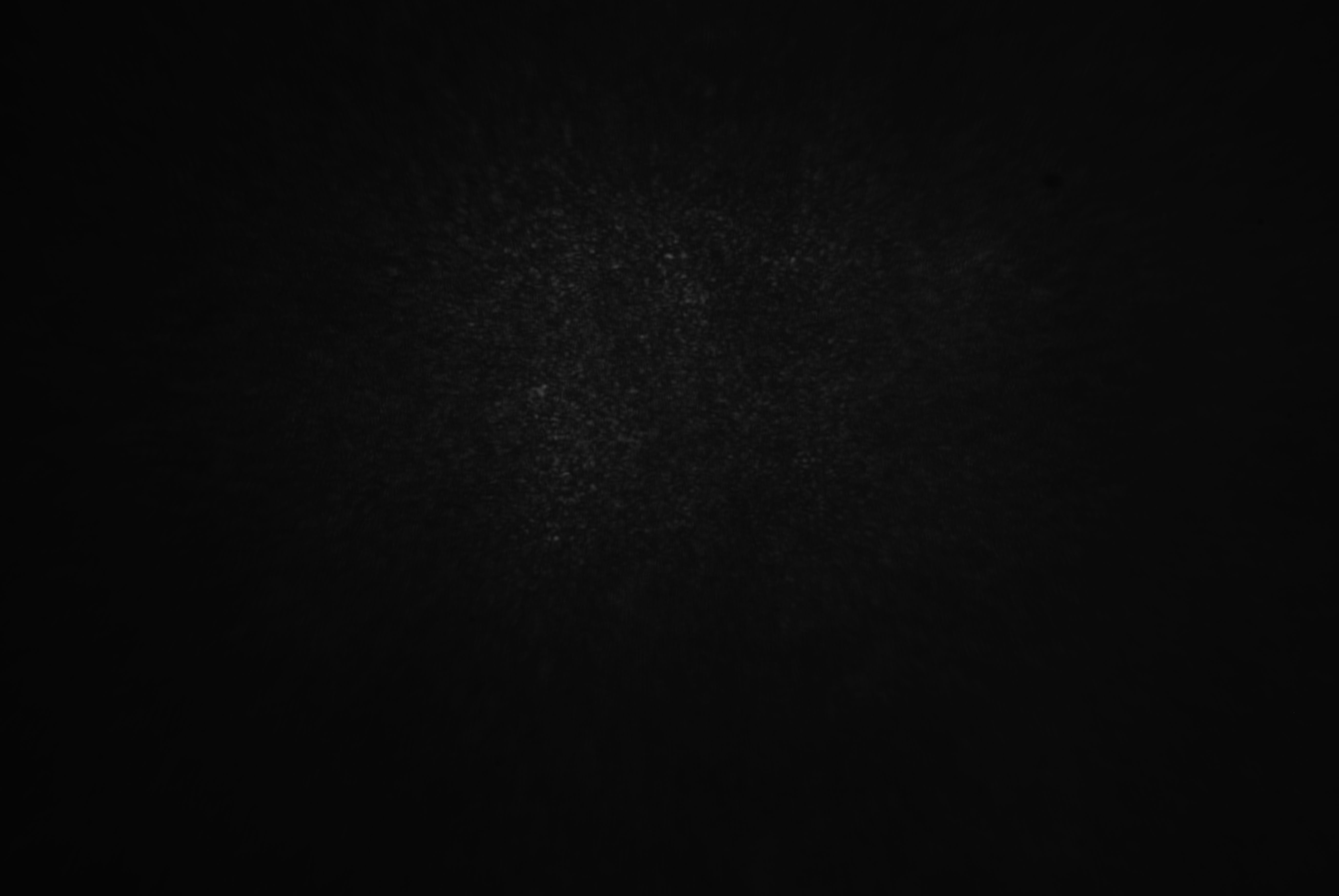

Supplement: Supplementary file 7 — Source Data [file 41467_2023_43674_MOESM7_ESM.zip › Source Data/Data 2/y (17).JPG]

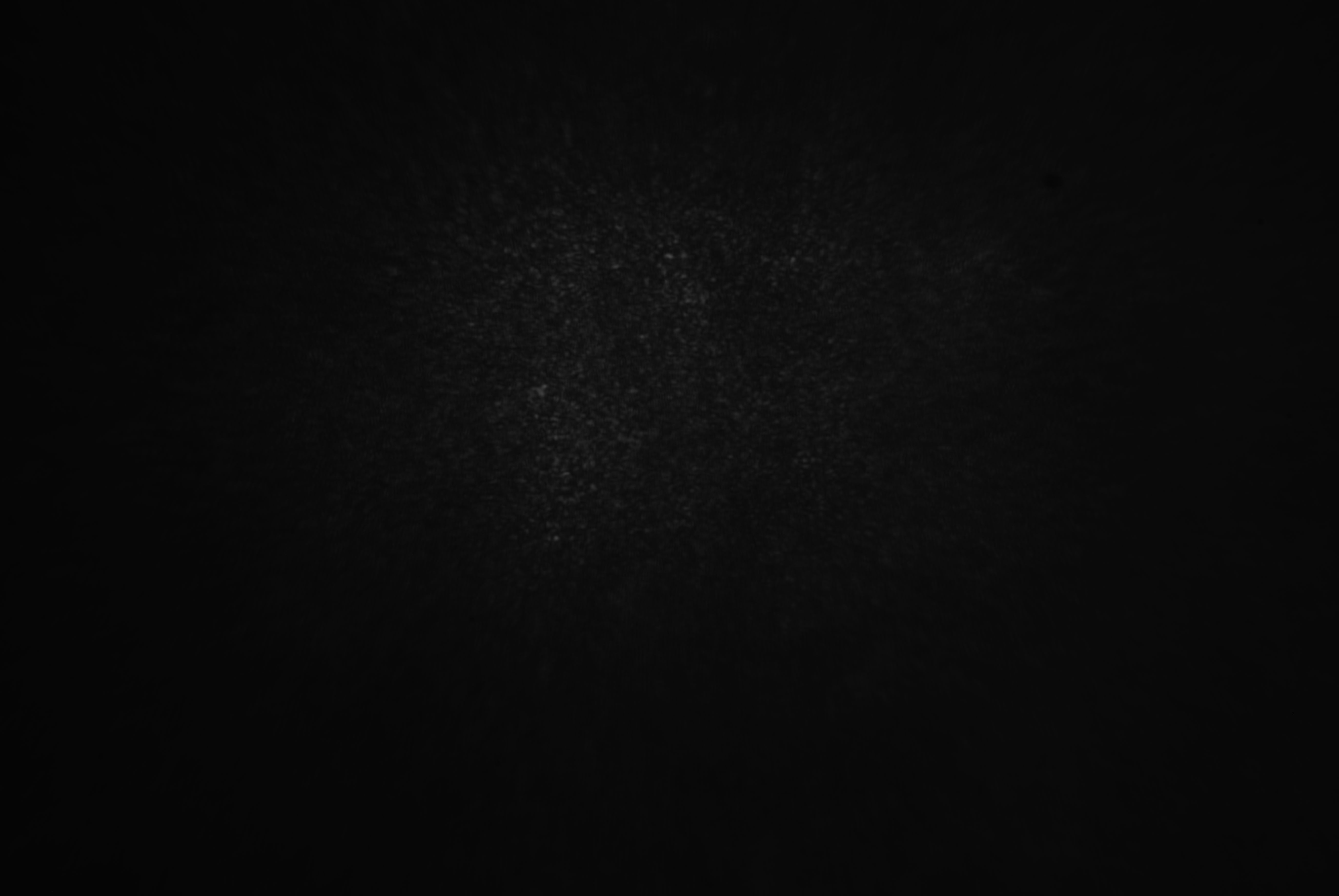

Supplement: Supplementary file 7 — Source Data [file 41467_2023_43674_MOESM7_ESM.zip › Source Data/Data 2/y (18).JPG]

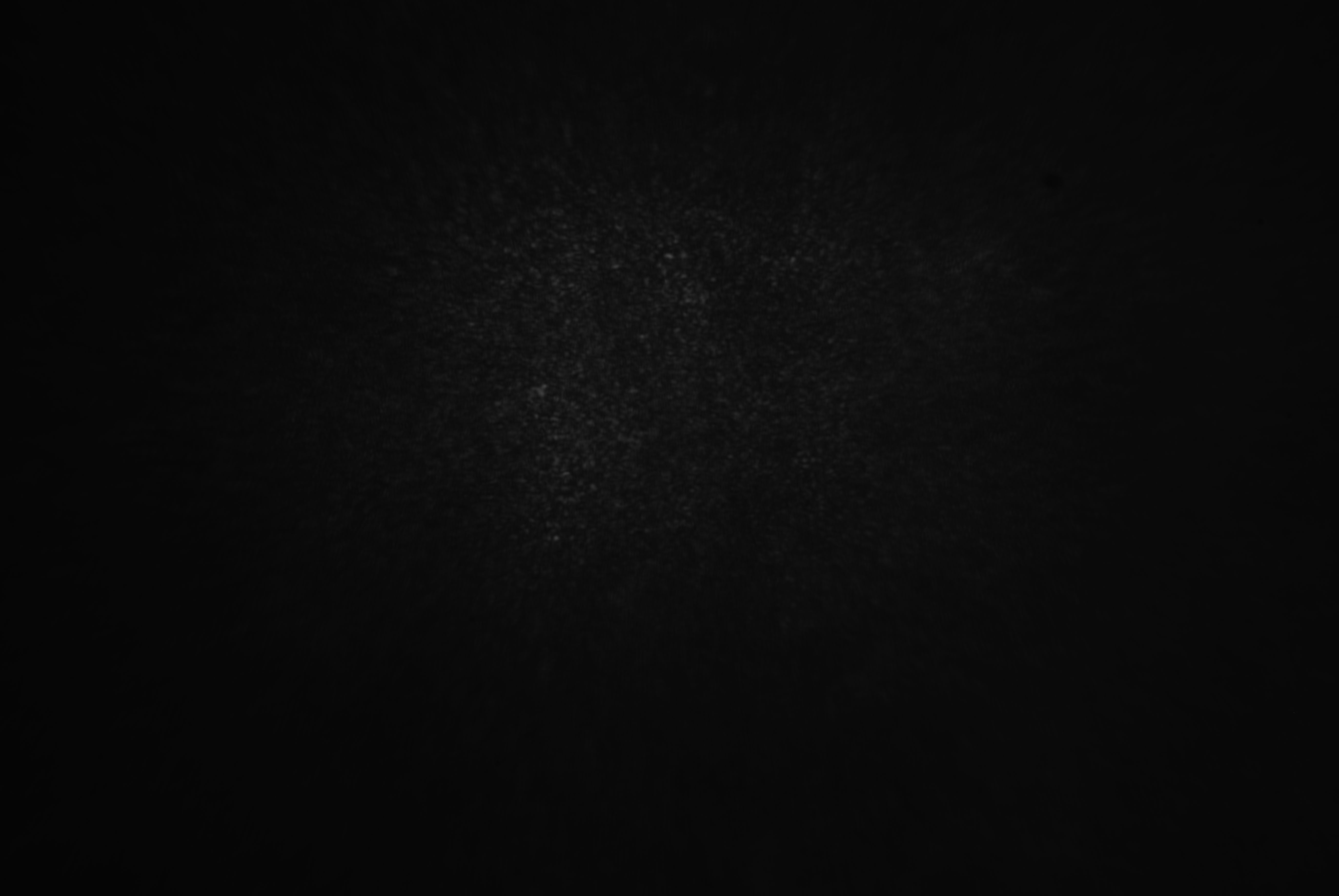

Supplement: Supplementary file 7 — Source Data [file 41467_2023_43674_MOESM7_ESM.zip › Source Data/Data 2/y (19).JPG]

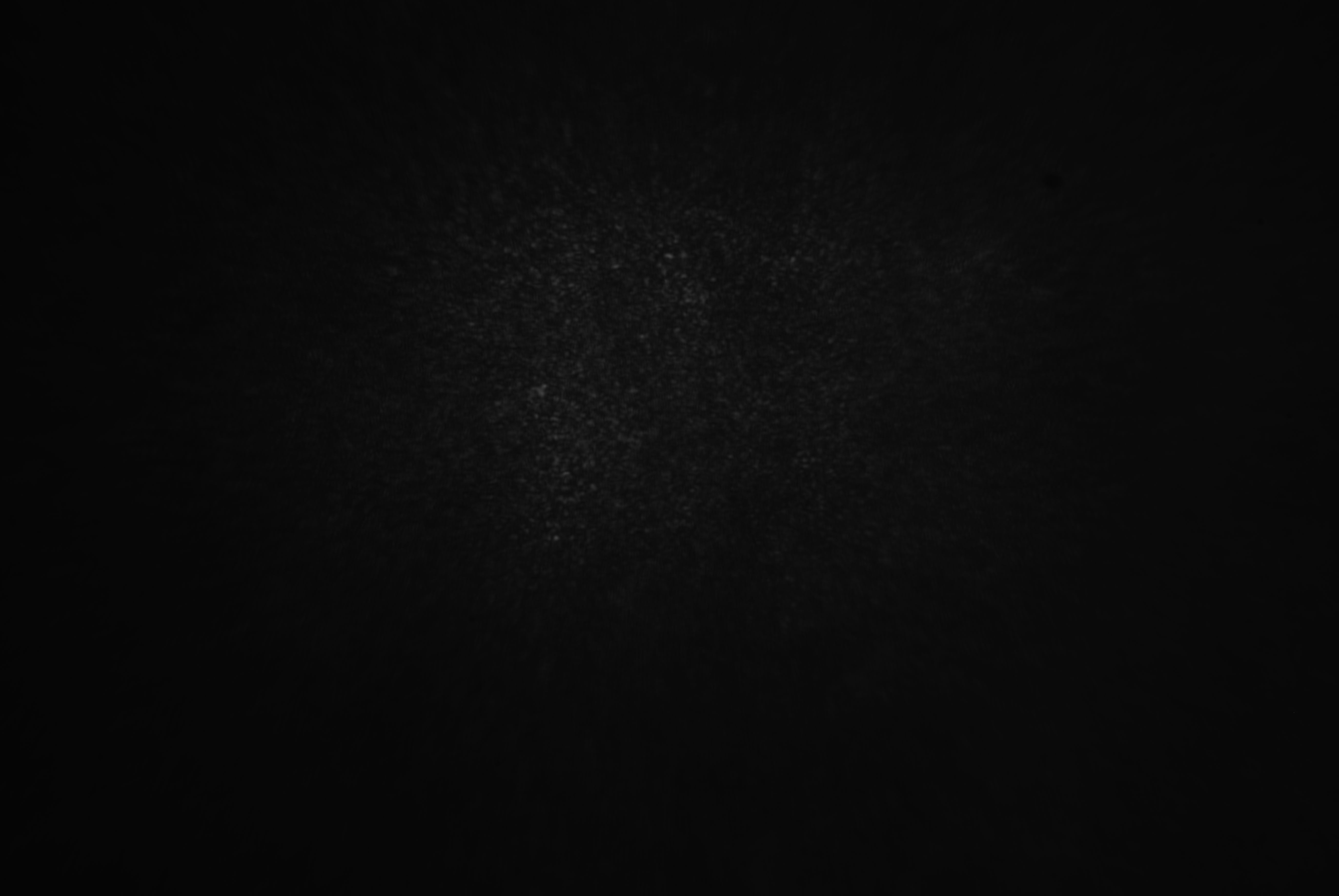

Supplement: Supplementary file 7 — Source Data [file 41467_2023_43674_MOESM7_ESM.zip › Source Data/Data 2/y (20).JPG]

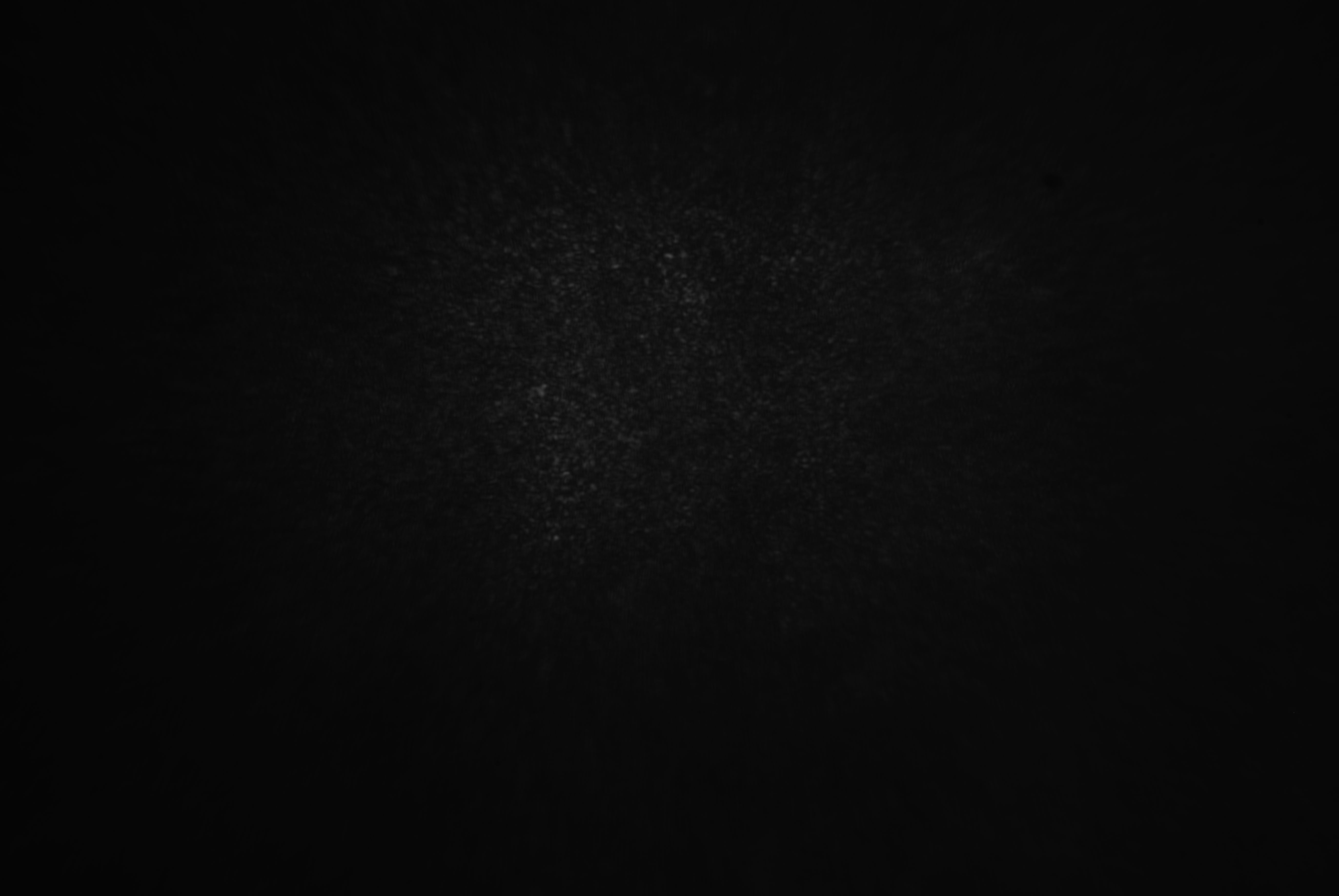

Supplement: Supplementary file 7 — Source Data [file 41467_2023_43674_MOESM7_ESM.zip › Source Data/Data 2/y (21).JPG]

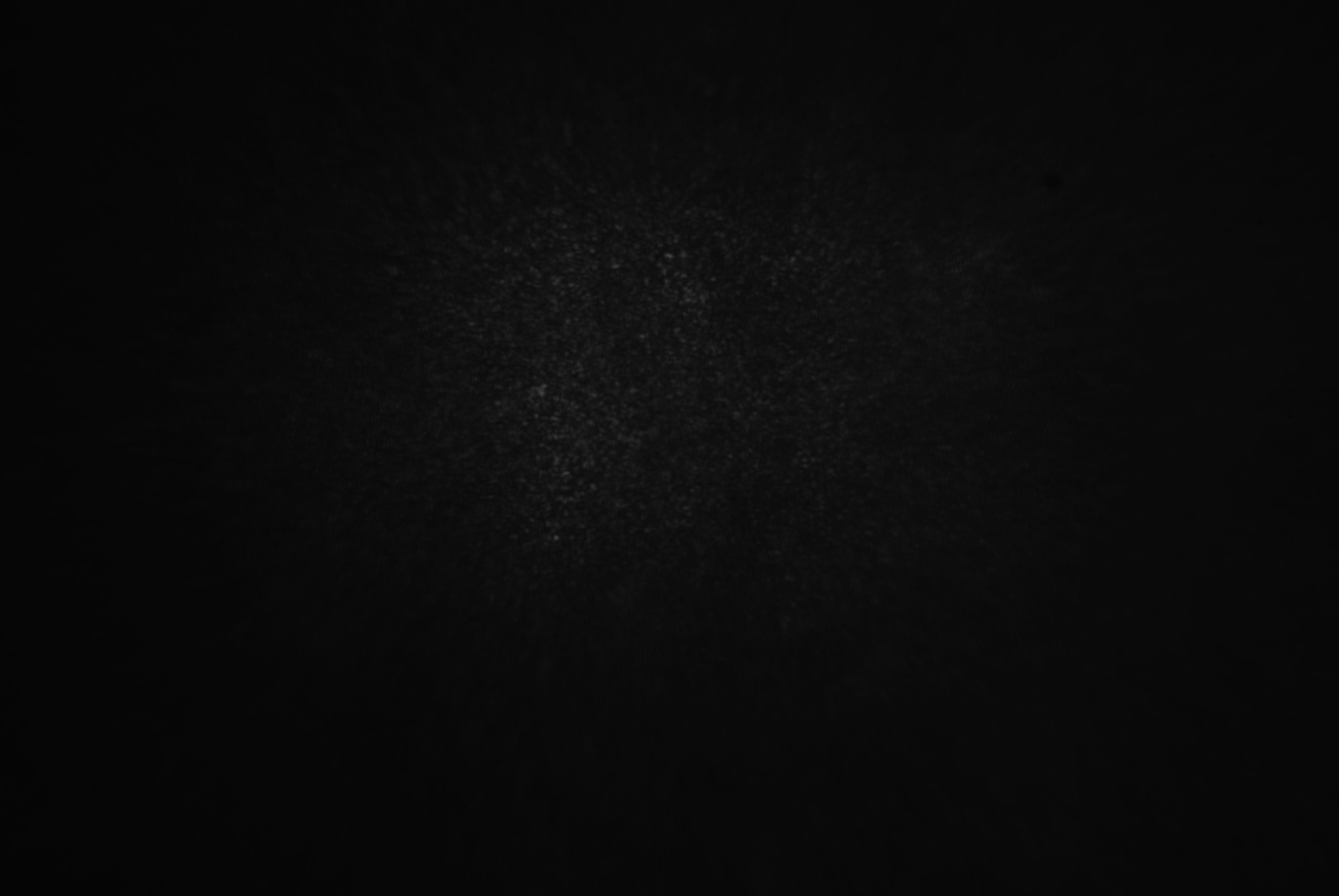

Supplement: Supplementary file 7 — Source Data [file 41467_2023_43674_MOESM7_ESM.zip › Source Data/Data 2/y (22).JPG]

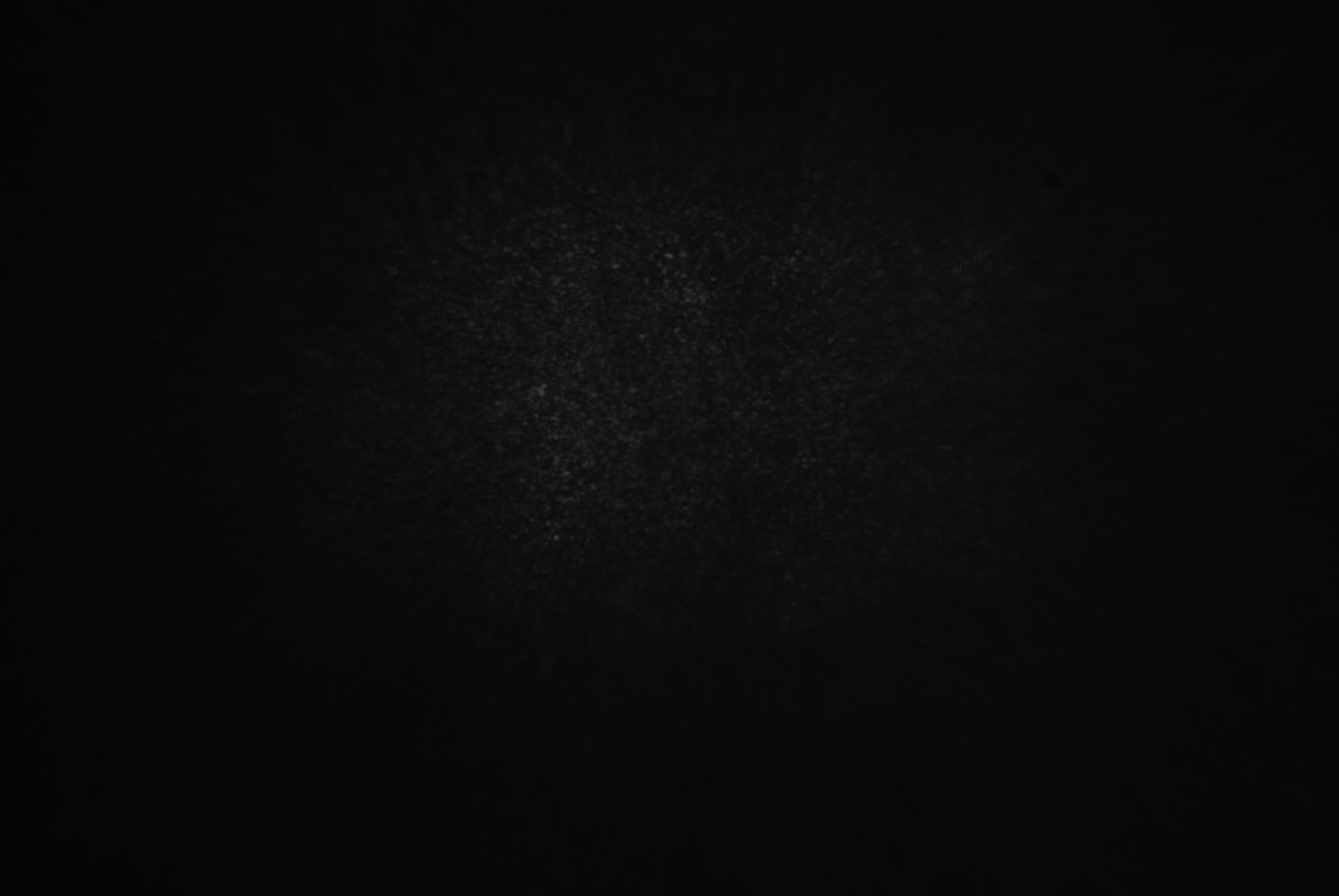

Supplement: Supplementary file 7 — Source Data [file 41467_2023_43674_MOESM7_ESM.zip › Source Data/Data 2/y (23).JPG]

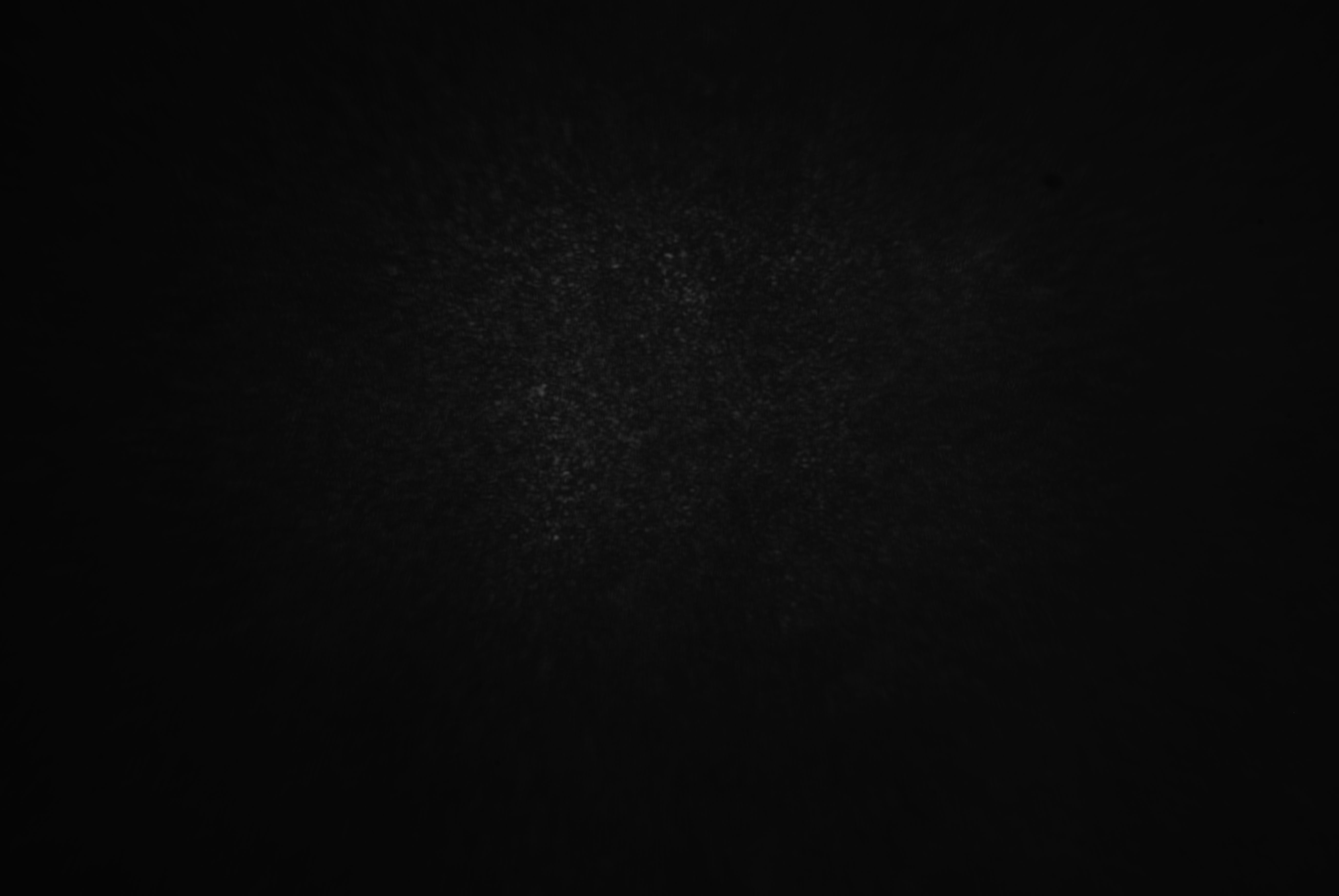

Supplement: Supplementary file 7 — Source Data [file 41467_2023_43674_MOESM7_ESM.zip › Source Data/Data 2/y (24).JPG]

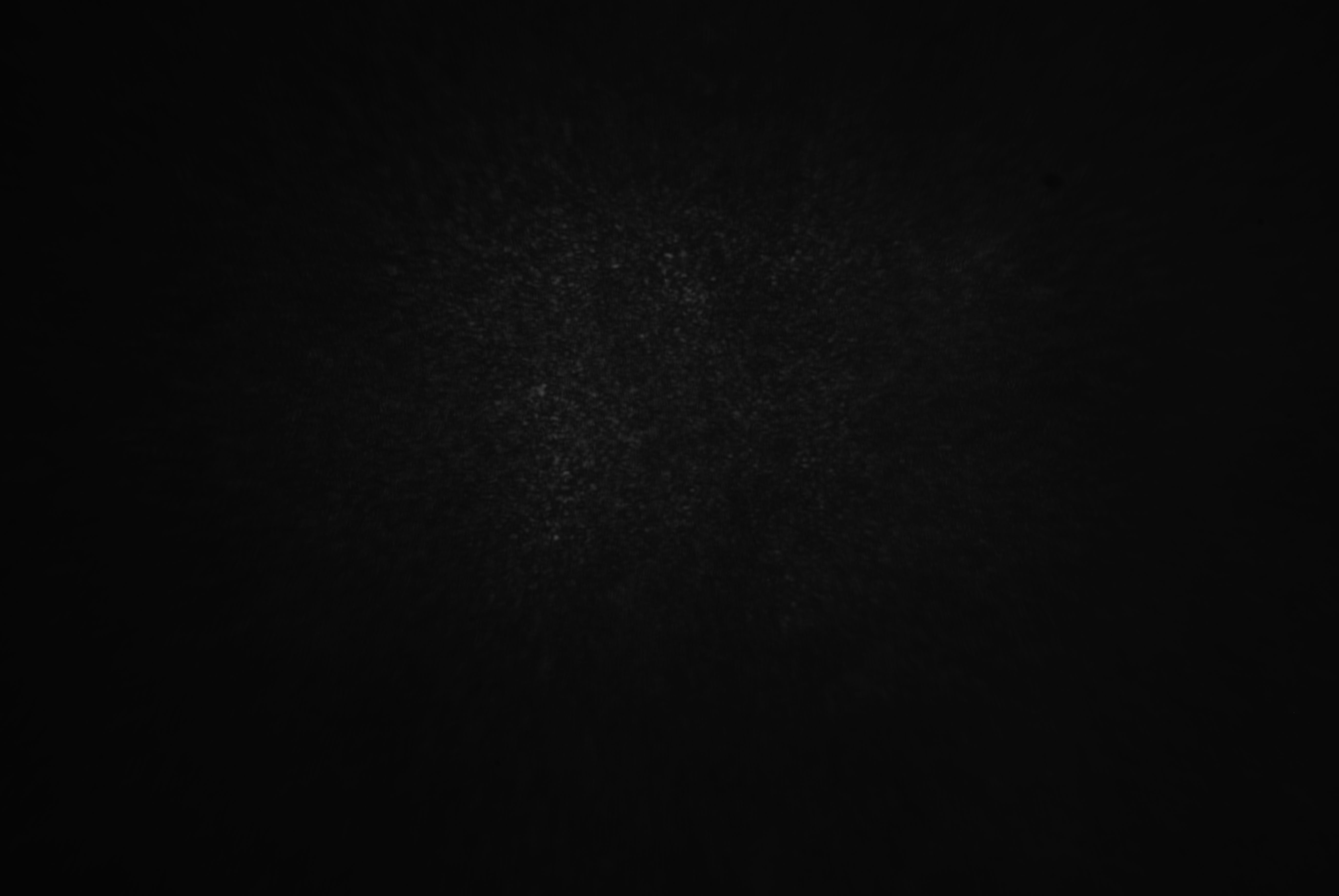

Supplement: Supplementary file 7 — Source Data [file 41467_2023_43674_MOESM7_ESM.zip › Source Data/Data 2/y (25).JPG]

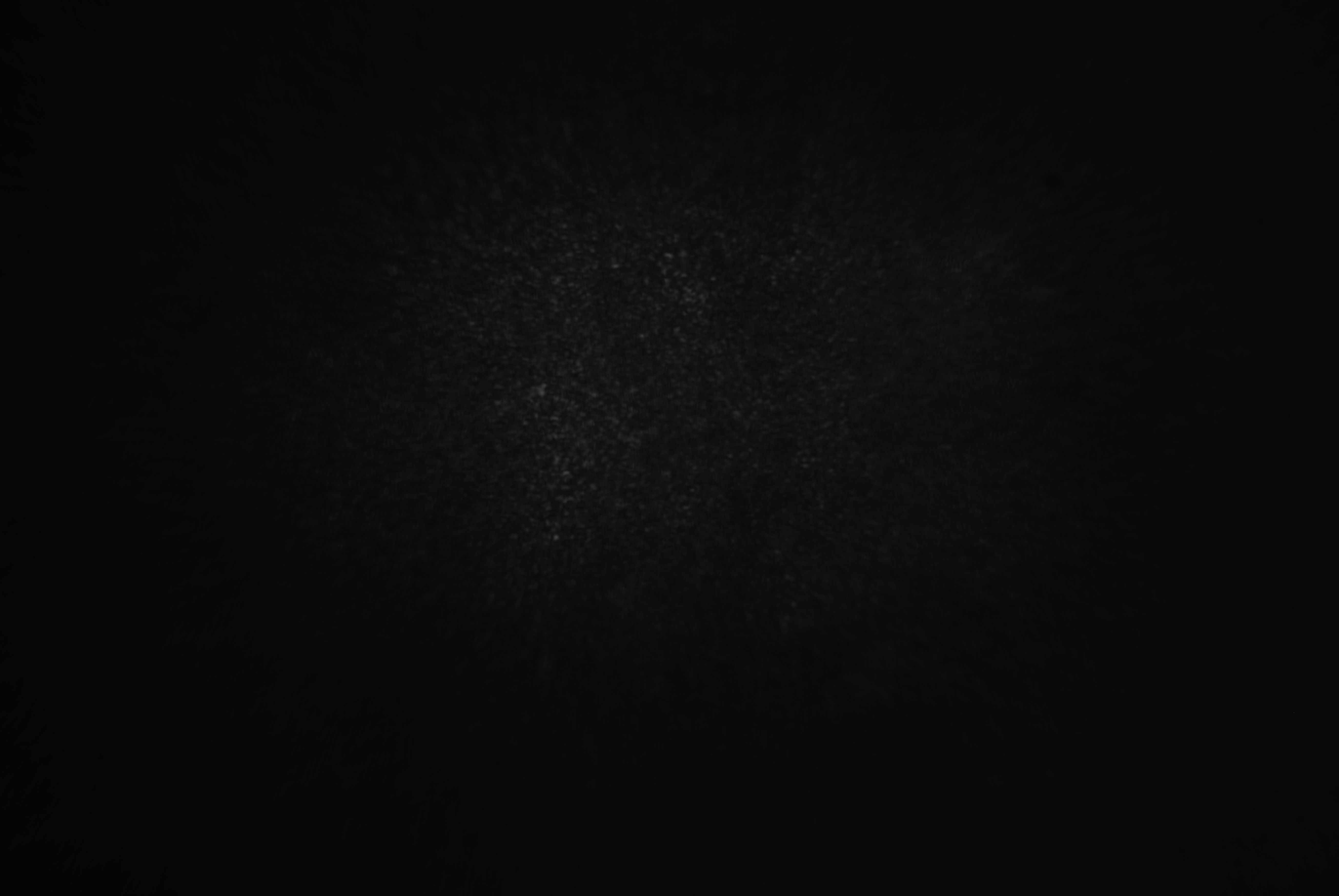

Supplement: Supplementary file 7 — Source Data [file 41467_2023_43674_MOESM7_ESM.zip › Source Data/Data 2/y (26).JPG]

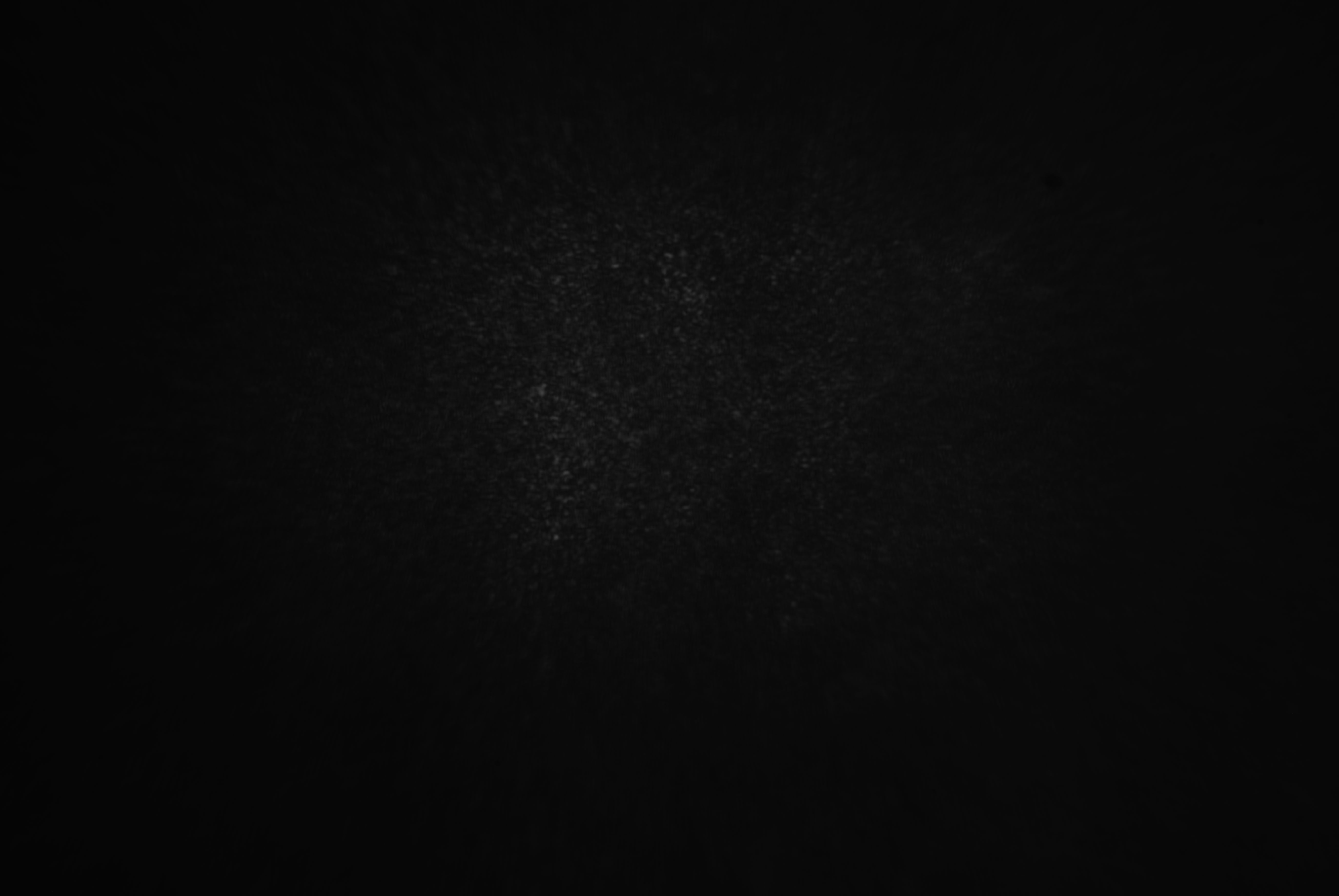

Supplement: Supplementary file 7 — Source Data [file 41467_2023_43674_MOESM7_ESM.zip › Source Data/Data 2/y (27).JPG]

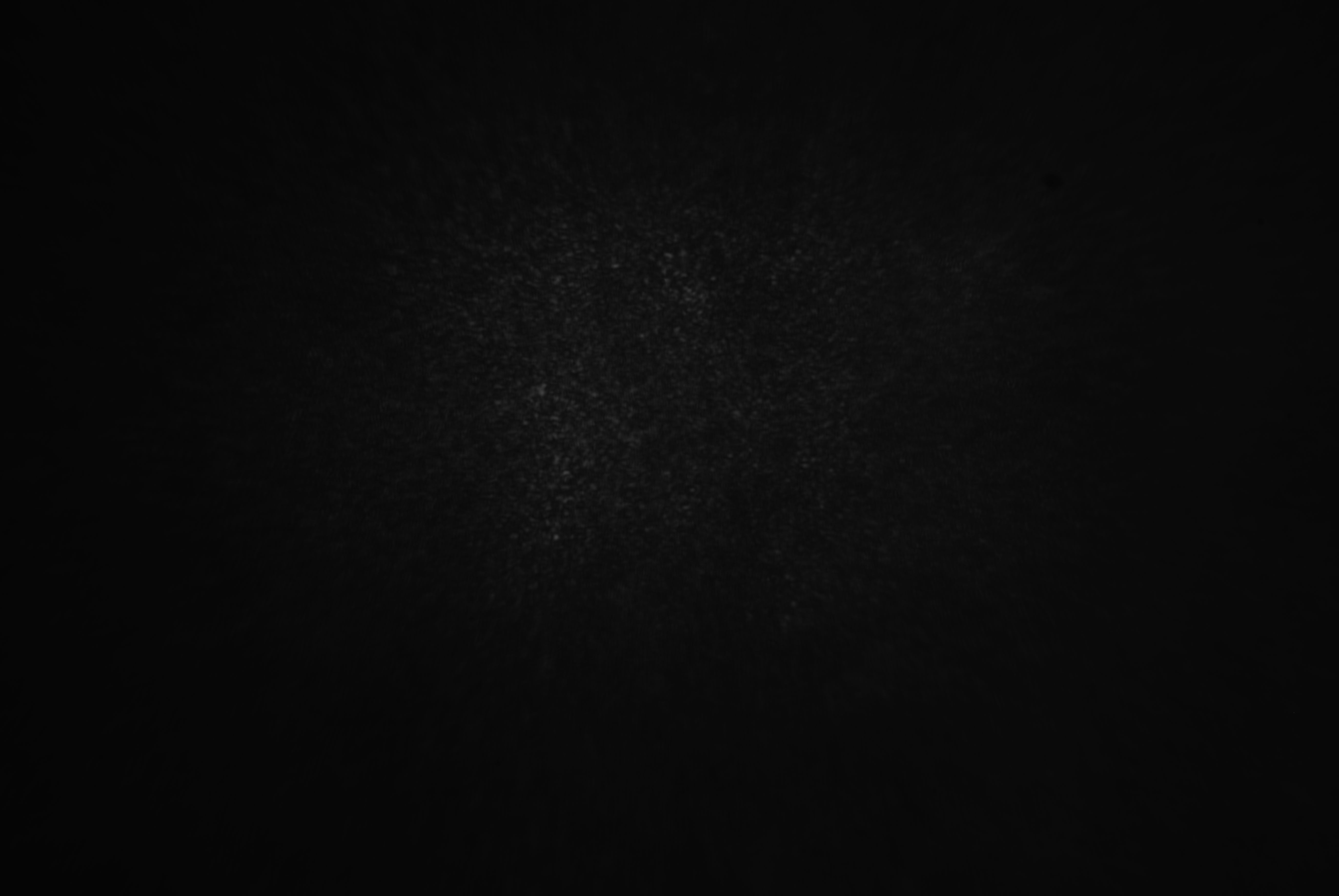

Supplement: Supplementary file 7 — Source Data [file 41467_2023_43674_MOESM7_ESM.zip › Source Data/Data 2/y (28).JPG]

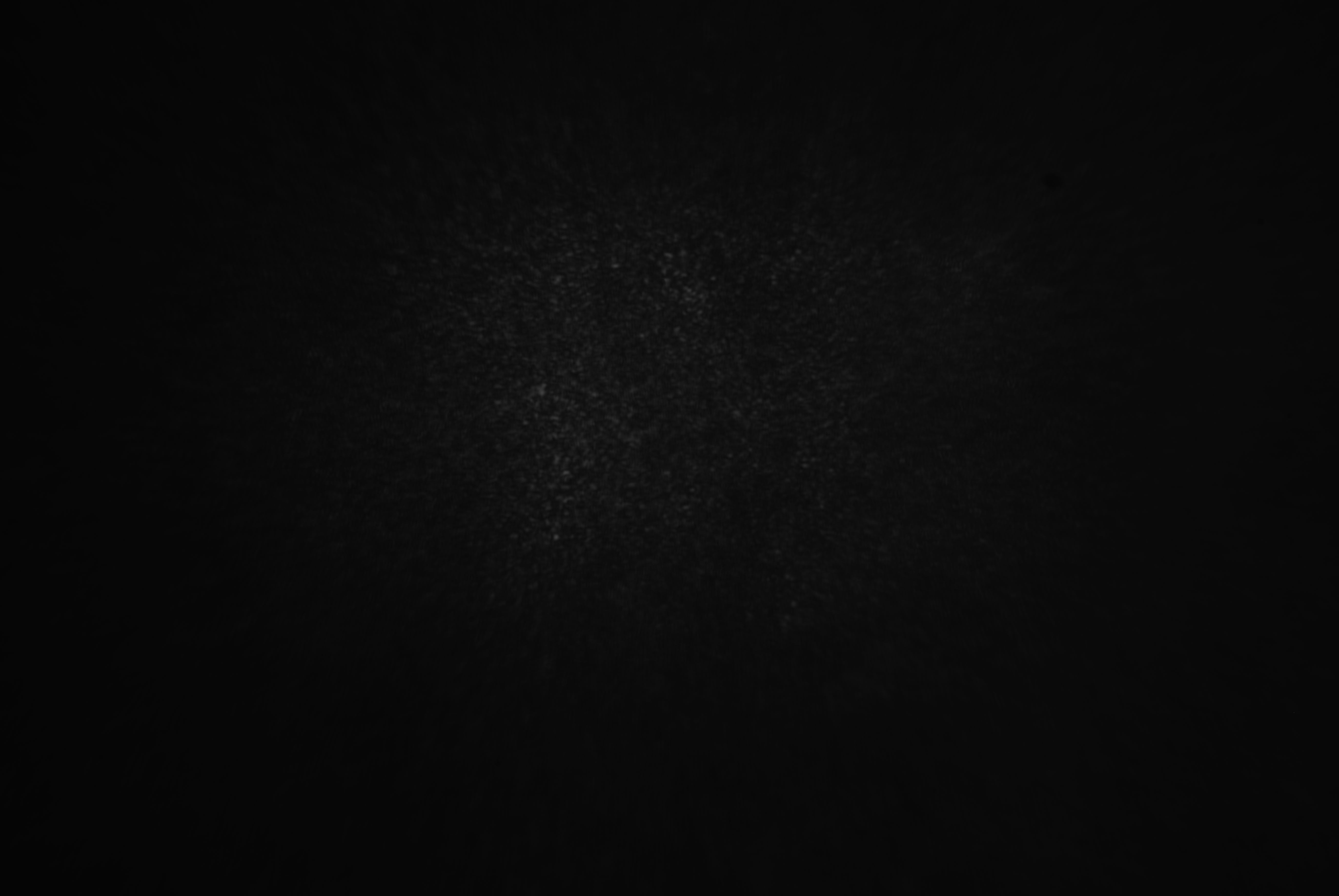

Supplement: Supplementary file 7 — Source Data [file 41467_2023_43674_MOESM7_ESM.zip › Source Data/Data 2/y (29).JPG]
